# WHO estimates of the global, regional, and national burden of 14 foodborne diarrhoeal enteric hazards, 2000–21: an updated data synthesis

**DOI:** 10.1016/j.langlo.2026.103997

**Published:** 2026-06-15

**Authors:** Shannon E Majowicz, Josh M Colston, Martyn D Kirk, Sara M Pires, Yuki Minato, Luria L Founou, Brecht Devleesschauwer, Carlotta di Bari, Teresa Estrada-Garcia, Louise Vaes, Arie H Havelaar, Charlee Roberts, Tesfaye Gobena, Ashok Kumar, Fadi Al Natour, Robin J Lake, Gabriela F Nane, Lucy J Robertson, Kim Fernandez, Elaine Scallan Walter, Margaret Kosek

**Affiliations:** School of Public Health Sciences, University of Waterloo, Waterloo, ON, Canada (Prof S E Majowicz PhD); Division of Infectious Diseases and International Health, University of Virginia School of Medicine, Charlottesville, VA, USA (J M Colston PhD, Prof M Kosek MD); National Centre for Epidemiology and Population Health, The Australian National University, Canberra, ACT, Australia (Prof M D Kirk PhD); National Food Institute, Technical University of Denmark, Kongens Lyngby, Denmark (S M Pires PhD); Department of Nutrition and Food Safety, World Health Organization, Geneva, Switzerland (Y Minato MPH, C Roberts MPhil (MAE)); Reproductive, Maternal, Newborn and Child Health (ReMARCH) Research Unit, Research Institute of the Centre of Expertise and Biological Diagnostic of Cameroon, Yaoundé, Cameroon (L L Founou PhD); Infection & Global Health Division, School of Medicine, University of St Andrews, St Andrews, UK (L L Founou PhD); Antimicrobial Research Unit, School of Health Sciences, College of Health Sciences, University of KwaZulu-Natal, Durban, South Africa (L L Founou PhD); Department of Epidemiology and Public Health, Sciensano, Brussels, Belgium (B Devleesschauwer PhD, C di Bari MSc, L Vaes MSc, K Fernandez MSc); Department of Translational Physiology, Infectiology and Public Health, Ghent University, Merelbeke, Belgium (B Devleesschauwer PhD, C di Bari MSc); Department of Molecular Biomedicine, CINVESTAV-IPN, Mexico City, Mexico (Prof T Estrada-Garcia PhD); Emerging Pathogens Institute, Global Food Systems Institute, Department of Animal Sciences, University of Florida, Gainesville, FL, USA (Prof A H Havelaar PhD); School of Environmental Health Sciences, College of Health and Medical Sciences, Haramaya University, Dire Dawa, Ethiopia (T Gobena PhD); Indian Council of Agricultural Research, Krishi Bhavan, New Delhi, India (A Kumar PhD); Abu Dhabi Agriculture and Food Safety Authority, Mohamed Bin Zayed City, Abu Dhabi, United Arab Emirates (F Al Natour DVM); Institute of Public Health and Forensic Science, Christchurch, New Zealand (R J Lake PhD); Delft Institute of Applied Mathematics, Faculty of Electrical Engineering, Mathematics and Computer Science, Delft University of Technology, Delft, Netherlands (G F Nane PhD); Parasitology Unit, Department of Paraclinical Science, Faculty of Veterinary Medicine, Norwegian University of Life Sciences, Ås, Norway (Prof L J Robertson PhD); Colorado School of Public Health, University of Colorado Anschutz Medical Campus, Aurora, CO, USA (Prof E Scallan Walter PhD)

## Abstract

**Background:**

Foodborne diseases cause significant illness and death globally. We updated WHO estimates of the burden caused by diarrhoeal hazards commonly transmitted by food: *Campylobacter jejuni*, *Campylobacter coli*, and other thermotolerant *Campylobacter* species; *Cryptosporidium* spp; *Cyclospora cayetanensis*; *Entamoeba histolytica*; enteroaggregative *Escherichia coli*; enteropathogenic *E coli*; enterotoxigenic *E coli*; *Giardia duodenalis*; norovirus; rotavirus; non-typhoidal *Salmonella enterica*; Shiga toxin-producing *E coli*; *Shigella* spp; and *Vibrio cholerae*.

**Methods:**

We estimated illnesses, deaths, and disability-adjusted life-years (DALYs) for 194 countries for the period 2000–21 using data from systematic reviews; the Global Burden of Diseases, Injuries, and Risk Factors Study 2021; a structured expert judgement study; and country consultations. We used disease-specific computational models, and a hierarchical meta-regression model with geographical clustering, a global linear time trend, and uncertainty propagation.

**Findings:**

In 2021, the 14 diarrhoeal hazards caused 666 million (95% UI 483–884) illnesses, 265 000 deaths (196 000–351 000), and 15·2 million (11·6–19·1) DALYs from foodborne transmission. *Shigella* spp, *Campylobacter*, and rotavirus caused the most DALYs from foodborne transmission. The greatest burden was in the African region (773·5 DALYs [95% UI 559·7–1033·3] per 100 000 population due to foodborne transmission). Mortality rates were 7·1 times higher and DALY rates 18·9 times higher in children younger than 5 years than in people aged 5 years or older. While the overall foodborne burden decreased between 2000 (692·3 DALYs [517·9–938·1] per 100 000 population) and 2021 (193·6 [147·2–243·0] per 100 000), this trend was not consistent for all hazards.

**Interpretation:**

Diarrhoeal hazards continue to cause a substantial foodborne disease burden, despite decreases over time. Children in low-income countries bear the greatest burden. Prevention requires concerted efforts, including expanding global diarrhoeal disease prevention efforts beyond water, sanitation, and hygiene and vaccination to include improvements in the safety of the food supply.

**Funding:**

WHO.

## Editorial note

The Lancet Group takes a neutral position with respect to territorial claims in published maps and institutional affiliations.

## Introduction

Diarrhoeal diseases are an important cause of illness and death,^1^ resulting in at least 2·21 billion illnesses and 881 000 deaths globally in 2021.^2^ Many causal hazards are transmitted by contaminated food, generating trade and economic impacts, and threatening achievement of the 2030 Sustainable Development Goals by damaging livelihoods and causing undernutrition.^1,3,4^ In 2015, WHO, with technical advice from the Foodborne Disease Burden Epidemiology Reference Group (FERG), estimated that foodborne transmission of 11 diarrhoeal enteric hazards resulted in 548 million illnesses, 200 000 deaths, and 15·8 million disability-adjusted life-years (DALYs) globally in 2010, with a disproportionately high burden in children younger than 5 years and people living in low-income and middle-income countries (LMICs).^1,3^

In 2021, following a mandate from the World Health Assembly,^5^ WHO reconvened the FERG to update global foodborne disease burden estimates and develop indicators to monitor progress in reducing foodborne diseases.^5,6^ In 2022, WHO member states adopted the first-ever foodborne disease reduction target, a 40% reduction in the incidence of foodborne diarrhoeal disease by 2030.^7^ WHO’s Global Health Strategy for 2025–28 subsequently incorporated an outcome indicator: the incidence of foodborne diarrhoeal non-typhoidal salmonellosis.^8^ Within FERG, the Enteric Diseases Task Force (EDTF) provided technical leadership on estimating foodborne enteric hazards, including the indicators, with support from WHO’s collaborator network.^6^ We report updated WHO estimates of the global, regional, and national foodborne disease burden for 194 countries for the period 2000–21, caused by 14 diarrhoeal enteric hazards: *Campylobacter jejuni*, *Campylobacter coli*, and other thermotolerant *Campylobacter* species (hereafter referred to as *Campylobacter*); *Cryptosporidium* spp; *Cyclospora cayetanensis*; *Entamoeba histolytica*; enteroaggregative *Escherichia coli*; enteropathogenic *E coli*; enterotoxigenic *E coli*; *Giardia duodenalis*; norovirus; rotavirus; non-typhoidal *Salmonella enterica*; Shiga toxin-producing *E coli*; *Shigella* spp; and *Vibrio cholerae*.

## Methods

### Overall approach

We followed a prespecified FERG protocol, detailed elsewhere,^6,9,10^ that included a computational framework and centralised analyses to estimate the foodborne burden of 14 hazards selected by the EDTF using a prioritisation matrix and process detailed elsewhere.^9,10^ The 14 hazards include the 11 previously estimated,^1^ plus *C cayetanensis*, enteroaggregative *E coli*, and rotavirus.

WHO commissioned a series of systematic reviews on the incidence, aetiology, sequelae, and mortality of these hazards that formed the basis of our estimation. We included additional data meeting review inclusion criteria, identified during internal FERG review and WHO’s country consultation process. We followed reporting guidelines for global health (GATHER)^11^ and burden of disease (STROBOD)^12^ studies (appendix 1 pp 61–70). Regional results are reported for the six WHO regions, subdivided into 17 subregions by World Bank income classifications.^6,9^ All decisions, data, review details, and assumptions are detailed and referenced in appendix 1 (pp 2–30).

### Estimating illnesses, deaths, and sequelae

We used WHO-commissioned estimates of incidence and mortality for the 14 hazards, from all routes of transmission and in two broad age strata (<5 years and ≥5 years), derived using methods reported elsewhere.^2^ Because of data availability differences, parallel approaches were used for subregion A countries and all other countries,^2,9,13,14^ as summarised in appendix 1 (pp 2–3). For subregion A countries (appendix 1 p 2) we assumed low diarrhoeal disease burden and higher surveillance capacity, and estimates were derived from national studies of population disease burden.^6,9^ For all other countries, aetiology proportions were derived from attributable fractions, and combined with draw-level diarrhoeal incidence estimates from the Global Burden of Diseases, Injuries, and Risk Factors Study (GBD) 2021, obtained directly from the Institute for Health Metrics and Evaluation (IHME), and draw-level diarrhoeal mortality estimates from WHO’s Global Health Estimates, stratified by age, sex, country, and year, for the years 2000 to 2021.

We incorporated sequelae into our disease models, including Guillain–Barré syndrome following *Campylobacter* infection, haemolytic-uraemic syndrome (HUS) following Shiga toxin-producing *E coli* infection (STEC-HUS), and end-stage renal disease following STEC-HUS. To estimate the number of Guillain–Barré syndrome illnesses following *Campylobacter* infection, we used draw-level Guillain–Barré syndrome incidence estimates from GBD 2021, obtained directly from the IHME, and stratified by age, sex, country, and year, for the years 2000 to 2021, minus the incidence due to COVID-19. We multiplied this estimate by the proportion of Guillain–Barré syndrome cases due to *Campylobacter* (appendix 1 p 4). To estimate the proportion of cases of Shiga toxin-producing *E coli* illness that developed into HUS, and the proportion of cases of STEC-HUS illness that developed into end-stage renal disease, we used results from a review and meta-analysis that estimated these proportions for Shiga toxin-producing *E coli* O157 and non-O157 (appendix 1 p 24). We also estimated death following sequelae (appendix 1 pp 6, 26).

We aimed to collect sex-disaggregated data and specified sex-specific distributions where data were available (appendix 1 pp 6–29).

### Estimating DALYs

To estimate DALYs, we calculated years lived with disability (YLDs) and years of life lost (YLLs) due to premature mortality following a standard approach detailed elsewhere.^9^ YLDs for a given health state are calculated as the product of the estimated number of total illnesses, duration of illness, and a disability weight. For each outcome, we used prevailing disability weights from GBD (appendix 1 pp 5–29). Duration of illness was based on previously published estimates and subject expert input (appendix 1 pp 5–29). YLLs were calculated based on the total deaths for each hazard and the WHO standard life expectancy at the age of death.

### Estimating the proportion of foodborne transmission

To estimate the proportion of each hazard transmitted by food, we used results of a WHO-commissioned structured expert judgement study.^15^ In this study, 94 experts provided 788 assessments for specific hazard–subregion combinations for the 14 hazards across 17 subregions. For *Shigella* spp in three subregions (Eastern Mediterranean region subregion D, South-East Asia region subregion B, Western Pacific region subregion B), attribution was determined by a global panel of experts due to insufficient numbers of subregional experts.^15^

### Data analysis

Analyses followed a standard computational framework to address data gaps and estimate illnesses, deaths, and DALYs by country-year, described in detail elsewhere.^9^ Briefly, we used a hierarchical meta-regression model with geographical clustering and a global linear time trend to smooth and impute country-year specific input data. Uncertainty in input parameters was propagated towards estimates of illnesses, deaths, and DALYs, through at least 500 Monte Carlo simulations following disease-specific computational models defined by incidence rates and transition probability parameters. Demographic data were sourced from the UN World Population Prospects 2024 Revision.

### Role of the funding source

WHO funded the reviews, review updates, and the structured expert judgement study (and helped recruit experts and elicitors) that provided the data used; the computations (Sciensano); and expert consultation (University of Florida). WHO managed the overall project and the country consultation process that identified additional data, while the EDTF determined whether data met inclusion criteria. WHO cleared the paper in terms of the methods and approach. WHO technical officers (YM and CR) participated in drafting the manuscript and the decision to submit it for publication.

## Results

Of the 2·21 billion diarrhoeal illnesses from all routes of transmission,^2^ 30·1% (95% uncertainty interval [UI] 24·7–35·4) were due to foodborne transmission, resulting in 666 million (483–884) foodborne diarrhoeal illnesses in 2021 (table). Of these illnesses, 11·0% (72·4 million [52·9–93·5]) were in children younger than 5 years. *Campylobacter* (148 million [83·5–239]), enterotoxigenic *E coli* (131 million [72·3–220]), *Shigella* spp (118 million [49·1–226]), and norovirus (54·8 million [28·0–97·1]) caused the most foodborne illnesses (table). This rank order of hazards also caused the highest rates of illness for both age groups (appendix 1 pp 33–34). Among children younger than 5 years, rotavirus was the fifth-leading cause of illnesses (6·50 million [2·66–11·5]), whereas Shiga toxin-producing *E coli* was the fifth-leading cause among people aged 5 years or older (47·0 million [18·5–103]). Children younger than 5 years had higher mean rates of foodborne illnesses than people aged 5 years or older for most hazards, except for *C cayetanensis*, *E histolytica*, *Shigella* spp, and *V cholerae* (appendix 1 pp 33–34). Data were not robust enough to generate sex-disaggregated estimates.

**Figure 1:**
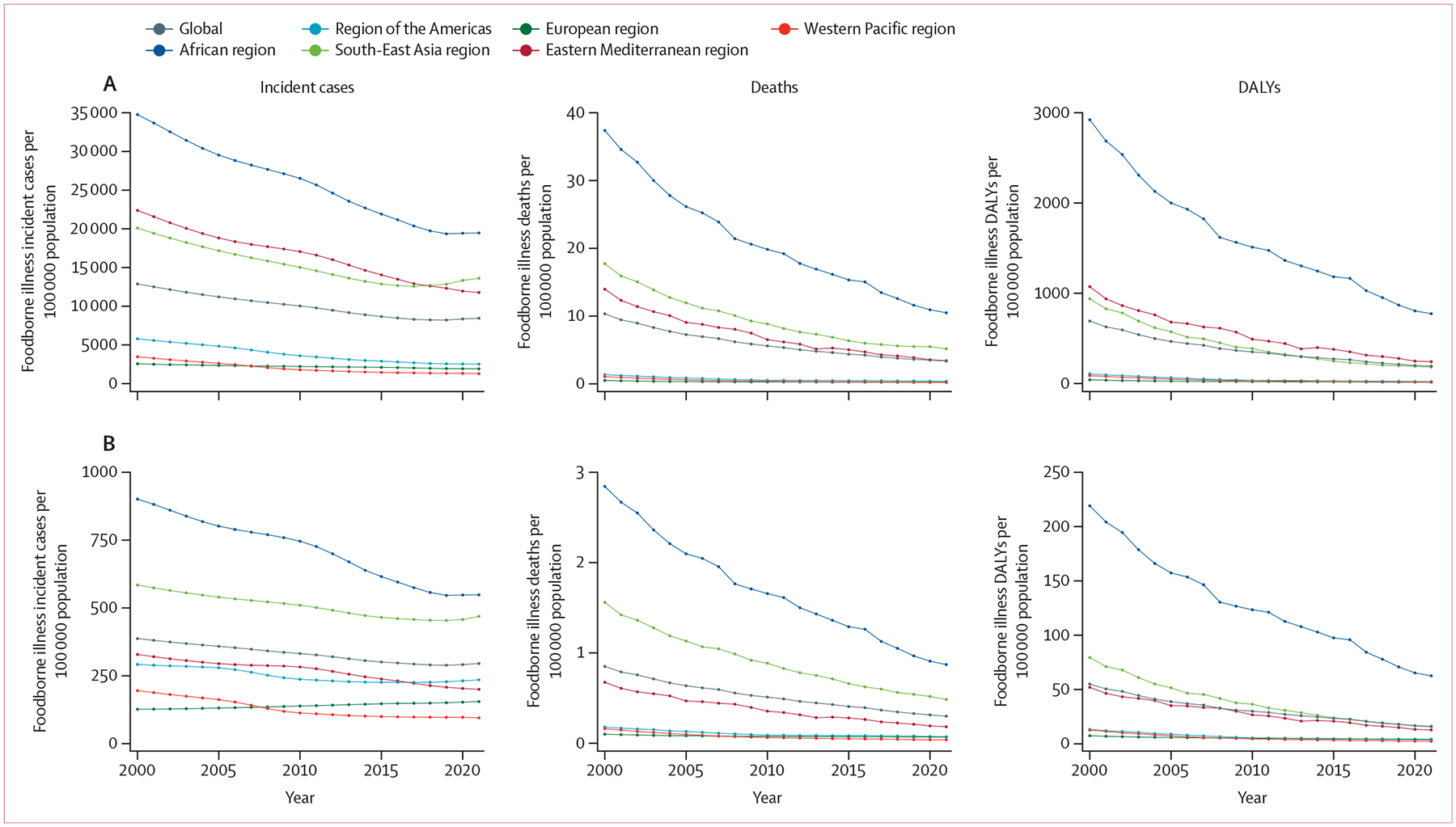
Annual foodborne diarrhoeal incidence, mortality, and DALYs per 100 000 population, for 14 diarrhoeal enteric hazards* (A) and non-typhoidal *Salmonella enterica* (B), globally and by WHO region, 2000–21 Data tables are provided in appendix 2. DALYs=disability-adjusted life-years. *Campylobacter jejuni, Campylobacter coli, and other thermotolerant *Campylobacter* species; *Cryptosporidium* spp; *Cyclospora cayetanensis*; *Entamoeba histolytica*; enteroaggregative *Escherichia coli*; enteropathogenic *E coli*; enterotoxigenic *E coli*; *Giardia duodenalis*; norovirus; rotavirus; non-typhoidal *S enterica*; Shiga toxin-producing *E coli*; *Shigella* spp; and *Vibrio cholerae*.

Our estimated incidence rates of foodborne diarrhoeal illnesses due to the 14 hazards overall, and to non-typhoidal *S enterica* specifically, were lower in 2021 (8450·1 foodborne diarrhoeal illnesses [95% UI 6132·4–11 218·3] per 100 000 population) than in 2000 (12 881·1 [9414·9–16 868·4] per 100 000) in most regions, although uncertainty intervals (appendix 2) overlapped (figure 1). Not all hazards showed this trend; for example, enteroaggregative *E coli*, enteropathogenic *E coli*, and Shiga toxin-producing *E coli* showed increases, while enterotoxigenic *E coli* was generally stable; *V cholerae* and *E histolytica* showed pronounced increases in the South-East Asia region since 2015, before which time trends were decreasing; and rotavirus showed the most pronounced decrease in the African region (appendix 1 pp 40–41).

**Figure 2:**
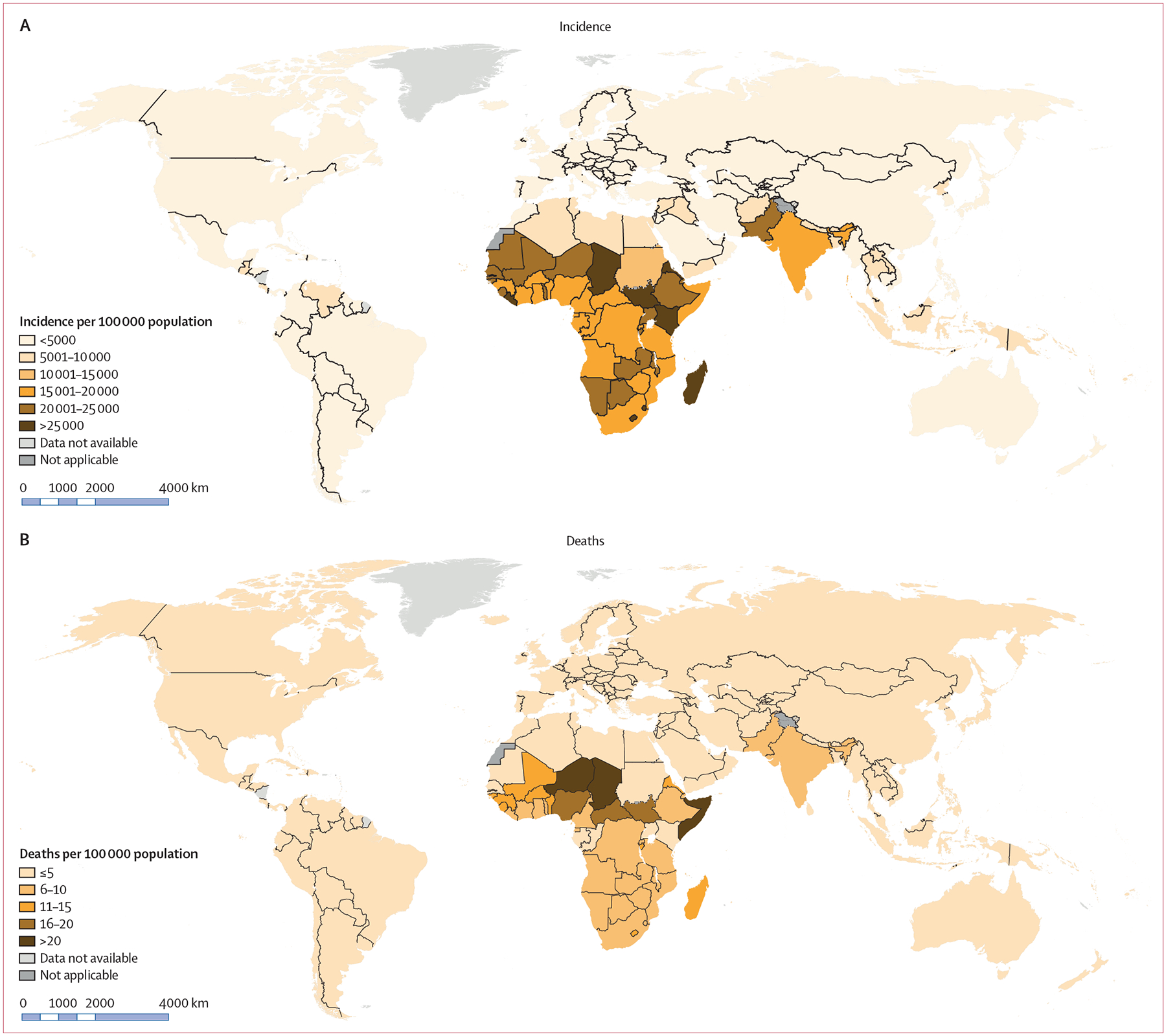

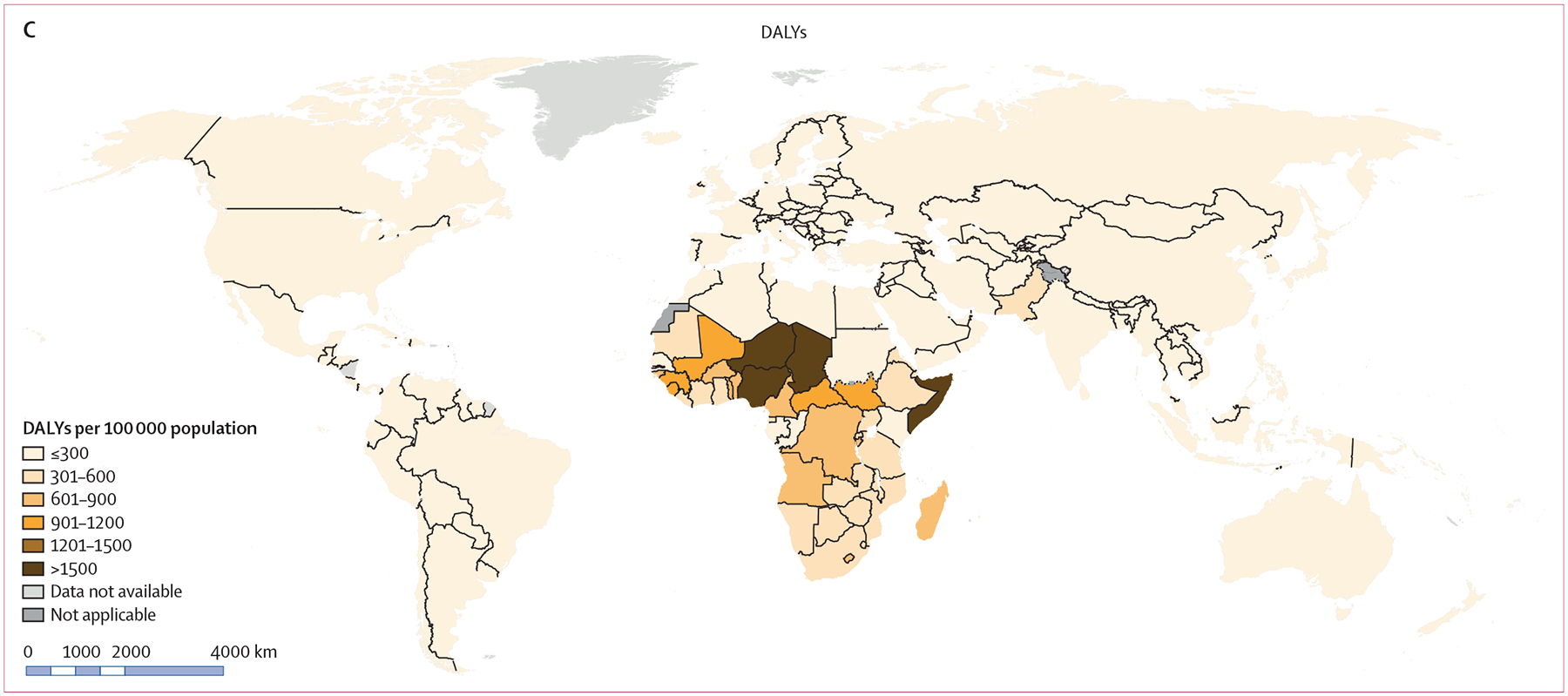
Mean national rates of foodborne illnesses (A), deaths (B), and DALYs (C) per 100 000 population due to 14 diarrhoeal enteric hazards, 2021 Data tables are provided in appendix 2. Map data © 2026 WHO. Dotted and dashed lines on maps represent approximate border lines for which there might not yet be full agreement. DALYs=disability-adjusted life-years. *Campylobacter jejuni, Campylobacter coli, and other thermotolerant *Campylobacter* species; *Cryptosporidium* spp; *Cyclospora cayetanensis*; *Entamoeba histolytica*; enteroaggregative *Escherichia coli*; enteropathogenic *E coli*; enterotoxigenic *E coli*; *Giardia duodenalis*; norovirus; rotavirus; non-typhoidal *Salmonella enterica*; Shiga toxin-producing *E coli*; *Shigella* spp; and *Vibrio cholerae*.

In 2021, the hazards causing the most foodborne diarrhoeal illness varied by WHO region (appendix 1 pp 35–39), although *Campylobacter* was one of the top three hazards causing foodborne illness in all six regions. The highest incidence rate of foodborne illness was in the African region (figure 2A; appendix 1 pp 35–39), followed by the South-East Asia and Eastern Mediterranean regions, where enterotoxigenic *E coli*, *Shigella* spp, and *Campylobacter* caused the most foodborne illnesses, although rank order varied. Further results, including national-level estimates, are available in WHO’s Global Health Observatory,^16^ with time trends for all hazards available via WHO’s interactive dashboard.^17^

In 2021, the 14 hazards resulted in 265 000 deaths (95% UI 196 000–351 000) from foodborne transmission (table). *Shigella* spp (42 500 deaths [19 100–80 400]), *Campylobacter* (35 400 [17 200–58 900]), enterotoxigenic *E coli* (28 900 [14 600–51 800]), and rotavirus (26 000 [6160–51 700]) were responsible for just over half of the foodborne diarrhoeal deaths globally. 40% (106 000 [77 600–136 000]) of all deaths due to foodborne diarrhoeal illness occurred in children younger than 5 years, in whom the hazards causing the most foodborne diarrhoeal deaths were rotavirus, *Campylobacter*, *Shigella* spp, enterotoxigenic *E coli*, and non-typhoidal *S enterica* (appendix 1 pp 33–34). Children younger than 5 years had higher mean rates of death per 100 000 population for all hazards, and overall mortality rates were 7·1 times higher in children younger than 5 years than in people aged 5 or older (appendix 1 pp 33–34). The hazards causing the most foodborne diarrhoeal deaths in people aged 5 years or older were *Shigella* spp, *Campylobacter*, and *V cholerae* (appendix 1 pp 33–34).

In 2021, the hazards causing the most deaths due to foodborne diarrhoeal illness varied by WHO region (appendix 1 pp 35–39). The highest rate of foodborne diarrhoeal deaths was in the African region (figure 2B; appendix 1 pp 35–39), followed by the South-East Asia region, and Eastern Mediterranean region; in all three regions, *Shigella* spp were one of the top three causes of death from foodborne diarrhoeal enteric hazards. *Shigella* spp caused the most foodborne diarrhoeal deaths in the African region (18 400 [95% UI 7960–31 800]), followed by rotavirus (17 400 [2260–36 300]) and enterotoxigenic *E coli* (17 000 [8020–30 300]). *Shigella* spp also caused the most foodborne diarrhoeal deaths in the South-East Asia region (19 700 [2540–48 700]), followed by *V cholerae* (15 400 [1730–48 000]) and *Campylobacter* (14 700 [5620–29 000]). In the Eastern Mediterranean region, *Campylobacter* caused the most foodborne illness deaths (5510 [2120–11 000]), followed by enterotoxigenic *E coli* and *Shigella* spp. In the region of the Americas and the European region, *Campylobacter* and non-typhoidal *S enterica* caused the most foodborne diarrhoeal deaths, whereas in the Western Pacific region it was rotavirus and non-typhoidal *S enterica* (appendix 1 pp 35–39).

Deaths due to foodborne diarrhoeal illnesses generally followed the same time trends as incidence, but in some regions the decline was more pronounced for deaths than for cases (figure 1). The global rate of foodborne diarrhoeal deaths per 100 000 population decreased from 10·3 (95% UI 7·6–14·4) in 2000, to 5·6 (4·4–7·1) in 2010, to 3·4 (2·5–4·5) in 2021.

**Figure 3:**
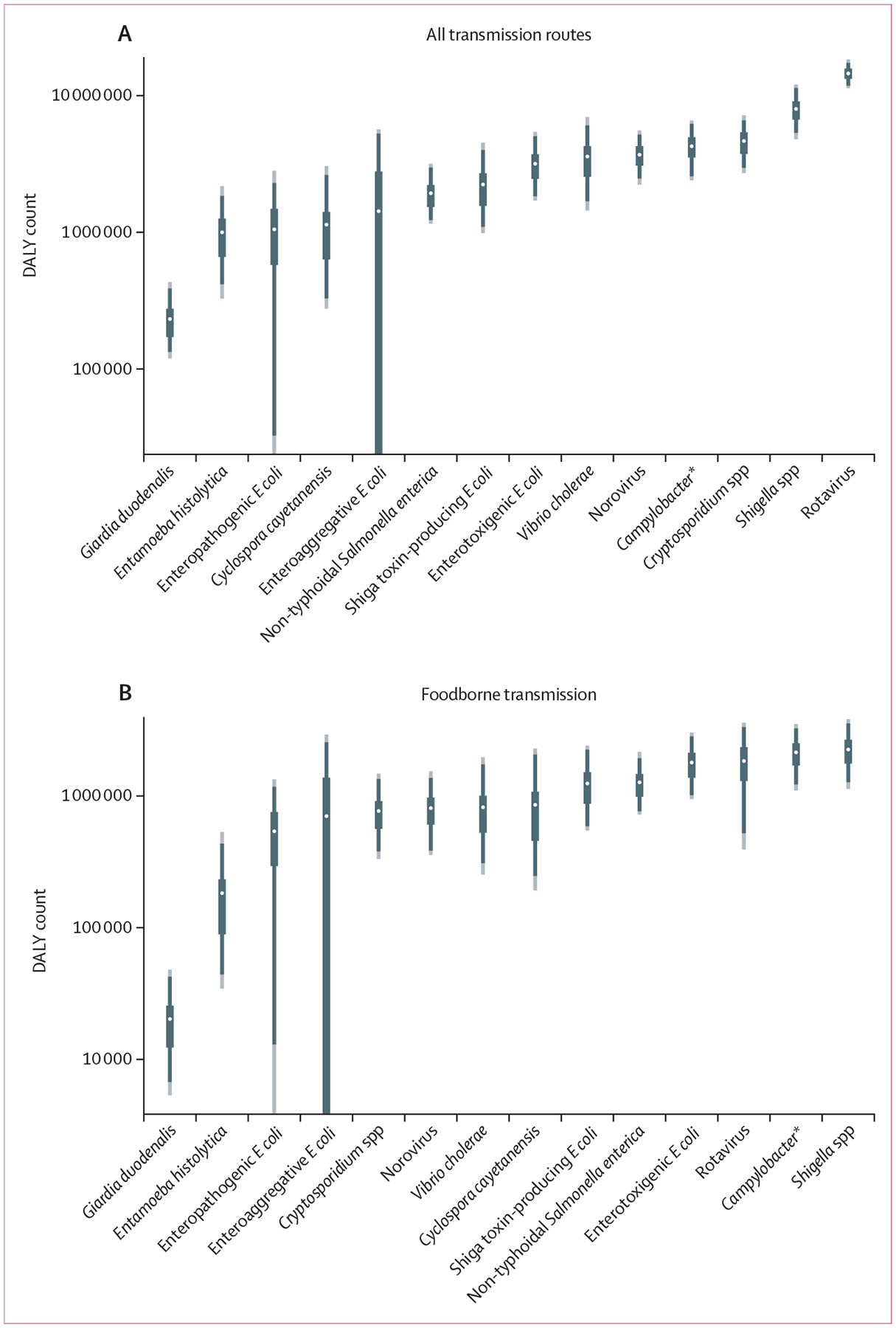
Mean DALYs globally for 14 diarrhoeal enteric hazards, from all routes of transmission (A) and foodborne transmission (B), ranked from lowest to highest, 2021 DALY estimates are presented as mean (dots), 50% UI (IQR, thick black line), 90% UI (thin black line), and 95% UI (thin grey line), on a logarithmic scale. Data tables are provided in appendix 2. DALYs=disability-adjusted life-years. UI=uncertainty interval. *Campylobacter jejuni, Campylobacter coli, and other thermotolerant *Campylobacter* species.

In 2021, the 14 hazards resulted in 50·9 million (95% UI 42·5–60·1) DALYs from all routes of transmission, with rotavirus, *Shigella* spp, and *Cryptosporidium* spp resulting in the most DALYs (table; figure 3A). In both 2000 and 2021, rotavirus and *Shigella* spp were the first and second ranked pathogens resulting in the most DALYs from all routes of transmission, followed by *Cryptosporidium* spp and *Campylobacter*, although the rank order of these two changed (appendix 1 pp 43–44).

In 2021, the 14 hazards resulted in 15·2 million (95% UI 11·6–19·1) DALYs through foodborne transmission (table), of which 64·0% (9·75 million [7·18–12·6]) occurred in children younger than 5 years, and 59% (9·00 million [6·52–12·0]) and 24% (3·73 million [2·29–5·60]) occurred in the African and South-East Asia regions, respectively. Most notably, 44% of the total burden (6·78 million [4·73–9·41]) was concentrated in children younger than 5 years in the African region. *Shigella* spp (2·25 million [1·13–3·82]), *Campylobacter* (2·15 million [1·10–3·51]), and rotavirus (1·84 million [0·392–3·60]) caused the most foodborne DALYs. In children younger than 5 years, the hazards causing the most DALYs through foodborne transmission were the same as those causing foodborne diarrhoeal deaths (rotavirus, *Campylobacter*, *Shigella* spp, enterotoxigenic *E coli*, and non-typhoidal *S enterica*; appendix 1 pp 33–34). Rates of DALYs due to foodborne transmission were higher in children younger than 5 years than in people aged 5 years or older for all hazards, with DALY rates 18·9 times higher in children younger than 5 years than in people aged 5 years or older (appendix 1 pp 33–34).

**Figure 4:**
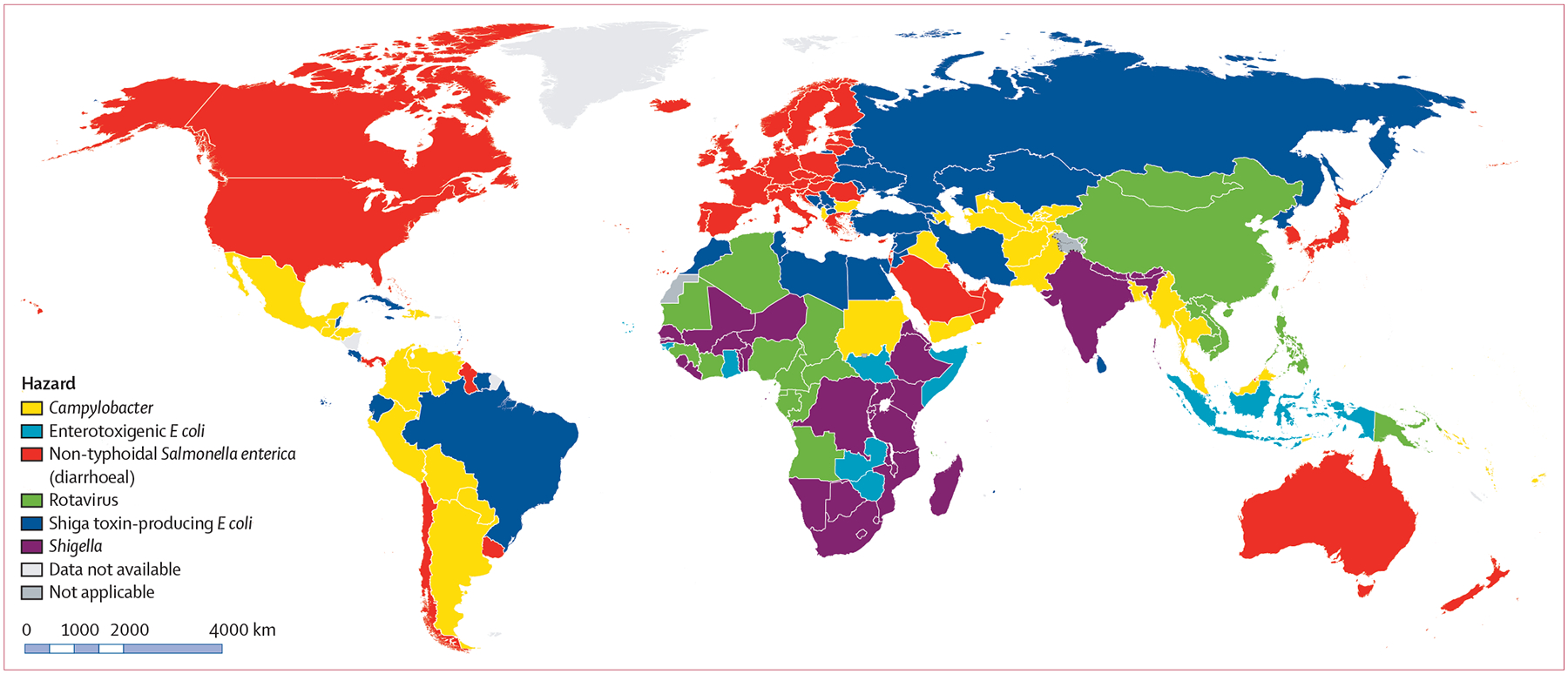
Diarrhoeal enteric hazards causing the most DALYs through foodborne transmission in 2021, by country Data tables are provided in appendix 2. Map data © 2026 WHO. Dotted and dashed lines on maps represent approximate border lines for which there might not yet be full agreement. DALYs=disability-adjusted life-years.

The highest burden of DALYs due to foodborne transmission in 2021 was in the African region (figure 2C; appendix 1 pp 35–39), with 773·5 DALYs (95% UI 559·7–1033·3) per 100 000 population, followed by Eastern Mediterranean region with 242·1 (159·9–355·6) per 100 000. *Shigella* spp, *Campylobacter*, and rotavirus resulted in the most DALYs due to foodborne diarrhoeal illness (table; figure 3B), although there was variation by region (appendix 1 pp 39–39, 45), and country (figure 4; appendix 1 pp 47–60). For all countries, the top hazard causing the most DALYs due to foodborne transmission was either *Campylobacter*, enterotoxigenic *E coli*, non-typhoidal *S enterica*, rotavirus, *Shigella* spp, or Shiga toxin-producing *E coli*.

In both 2000 and 2021, *Shigella* spp and *Campylobacter* resulted in the most DALYs due to foodborne transmission, and enteroaggregative *E coli*, enteropathogenic *E coli*, *E histolytica*, and *G duodenalis* resulted in the fewest, although the rank order changed within these highest and lowest groups across the two timepoints (appendix 1 pp 43–44). DALYs due to foodborne transmission generally followed the time trends of deaths (figure 1); the global rate of foodborne diarrhoeal DALYs per 100 000 population decreased from 692·3 (95% UI 517·9–938·1) in 2000, to 350·6 (278·5–440·1) in 2010, to 193·6 (147·2–243·0) in 2021.

## Discussion

Our findings confirm that, despite a reduced burden in 2021 compared with 2000, consistent with general declines in incidence and mortality of diarrhoea globally,^18^ diarrhoeal hazards transmitted through contaminated food continue to cause a substantial disease burden, resulting in 15·2 million DALYs in 2021. This burden was greatest in young children, who accounted for 40% of foodborne diarrhoeal deaths despite comprising just 9% of the global population, and in people living in LMICs. *Campylobacter*, enterotoxigenic *E coli*, and *Shigella* spp were the leading causes of foodborne illnesses and deaths. After these three hazards, norovirus and Shiga toxin-producing *E coli* were leading causes of foodborne illnesses, whereas rotavirus and non-typhoidal *S enterica* were leading causes of foodborne diarrhoeal deaths. *Shigella* spp, *Campylobacter*, rotavirus, enterotoxigenic *E coli*, and non-typhoidal *S enterica* were the leading causes of foodborne DALYs.

These updated WHO estimates are higher than previous WHO estimates of foodborne diarrhoeal disease from 2010,^1,3^ although direct comparisons are difficult due to methodological differences and an expanded list of hazards. We addressed this by generating annual estimates for 2000–21, including providing new estimates for 2010 that include rotavirus, enteroaggregative *E coli*, and *C cayetanensis*. Hazard rankings differ from the estimates from 2010, where norovirus was the leading cause of foodborne diarrhoeal illnesses and deaths.^1,3^ Other notable differences were our higher estimated number of foodborne illnesses due to Shiga toxin-producing *E coli*, a lower estimated number of foodborne diarrhoeal deaths due to enteropathogenic *E coli*, and lower estimated numbers of foodborne illnesses and deaths due to non-typhoidal *S enterica*. The African region continued to have the highest burden of foodborne diarrhoeal disease, despite the reduction in non-typhoidal *S enterica*, a leading contributor to foodborne burden in 2010. Rotavirus was a leading cause of foodborne diarrhoeal deaths in the African region and *V cholerae* remained an important cause of death in the South-East Asia region, despite the low percentage of total cases estimated to be foodborne.

Rotavirus, newly added to this round of estimates, was an important cause of foodborne diarrhoeal illness and the leading cause of foodborne diarrhoeal deaths among children younger than 5 years, despite being in rapid decline. This prominence is notable given the introduction and widespread adoption of the rotavirus vaccine in recent decades that has resulted in important declines in rotavirus deaths across the globe.^19^ The aetiology proportions we used accounted for country-level rotavirus vaccine introduction status.^2^ The contribution of rotavirus to the burden of foodborne diarrhoeal diseases in children, particularly in the African region, highlights the need to increase rotavirus vaccine implementation and coverage in high-mortality settings to further improve the impact of this important vaccine.

While we used broadly similar methods to those used previously,^1,3^ improved data availability, diagnostics, and statistical methods likely contributed to the differences between estimates noted above. There has been a proliferation of high-quality studies assessing diarrhoeal disease incidence, mortality, and aetiology in the past decade,^14^ allowing our estimates to draw on 37 national population-level incidence or mortality studies and 287 health-facility-based and community-based diarrhoea aetiology studies. Moreover, since 2010, diagnostics have shifted from culture and microscopy to nucleic-acid-based methods. These methods are generally more sensitive and likely to detect bacteria such as *Campylobacter* that are difficult to consistently isolate in culture,^20^ and are often run in large panels, meaning a single test informs on a wider breadth of pathogens, thereby providing a more balanced source of information on which to base estimates. The higher relative rank of *Campylobacter* is an expected outcome of this diagnostic shift.

In addition to previous WHO estimates,^1,3^ other relevant diarrhoeal disease global burden studies exist. In 2021, GBD estimated that 59·0 million DALYs were attributable to diarrhoeal diseases.^21^ This study considered neonatal factors (eg, low birthweight) and access to improved water, sanitation, and hygiene (WASH), and estimated disease burden avoidable by policies to improve WASH and vaccine access. Our findings extend this work by estimating the burden avoidable by reducing foodborne transmission. Diarrhoeal deaths have also recently been estimated by Black and colleagues in children younger than 5 years.^22^ Our study differs from Black and colleagues’ study most notably by our focus on foodborne transmission, estimates of incidence and DALYs, and our inclusion of all ages. There are certain predictable differences in the relative burdens of certain pathogens, including *V cholerae* and *Campylobacter*, which cause an important disease burden in non-paediatric populations. However, in both studies, rotavirus and *Shigella* spp were the top two causes of death (in this study, from all routes of transmission), showing consistency of findings. Our study extends previous estimates by highlighting the importance of foodborne transmission in addition to other important risk factors.

Efforts to prevent foodborne diarrhoeal diseases should prioritise reducing disease burden in general, and in young children and people living in LMICs in particular. Measures focused on reducing overall burden, such as improvements in health care and treatment (eg, antibiotics administered in all cases of dysentery and cholera; and oral rehydration administered in all cases of diarrhoea) continue to be important. Additionally, interventions along the food production chain from source to primary processing practices, distribution, preparation, and storage of foods are fundamental for a safe and secure food supply.^23^ Improvements in animal husbandry, food chains, and household hygiene all have the capacity to reduce transmission, although multiple interventions are required to maintain food safety.^24,25^ Efforts to reduce the burden in children include improving the safety of complementary food, and widespread implementation of existing rotavirus and *V cholerae* vaccines and others as they come to market. The national burden estimates presented in this Article, together with source attribution estimates, provide useful information for countries to rank locally important hazards, prioritise programmes and interventions, and ultimately reduce foodborne diseases using interventions adapted to regional and local food environments.

The estimates reported here also offer a baseline against which to measure such progress, including via WHO’s two new food safety indicators. Values for these indicators at the global, regional, and national levels can be used to monitor progress towards target reductions. While our results generally show global and regional declines in these incidence indicators from 2000 to 2021, the substantial burden from foodborne diarrhoeal diseases in 2021 suggests more progress is needed.

There are several limitations, most notably that the findings likely underestimate the true burden of foodborne diarrhoeal diseases, particularly in LMICs. The 14 hazards we included represent major foodborne pathogens, but do not capture all those that are important at the subregional and national levels. For some excluded hazards, we did not have sufficient data, whereas for others the proportion that were foodborne was assumed to be low (eg, adenovirus and sapovirus). Additionally, important hazards continue to be recognised, including enteroaggregative *E coli* in high-income settings such as western Europe,^26^ and foodborne astrovirus in a growing number of LMIC settings.^27^

The scarcity of reliable data is a recurring challenge. Although availability has improved, data remain heterogeneous across LMICs; for example, research centres that provide large amounts of high-quality local data might not broadly represent national or regional populations. We addressed this in part by requesting additional data during country consultation, but limited additional datapoints emerged. Moreover, while we restricted input data to the highest quality evidence available, remaining data gaps meant that some decisions relied on expert judgement. Due to data gaps, we were only able to include selected sequelae, also meaning that our findings are likely underestimates, as the burden from sequelae such as linear growth faltering can be substantial at the population level.^28^ Data limitations also restricted our ability to include hazard-specific variations in some model inputs (eg, differences in diarrhoea duration, severity, and sequelae outcomes across hazards, species, and settings),^29,30^ and examine other important drivers of burden, including antimicrobial resistance and comorbidities.

Finally, estimating the proportion of illness acquired from food poses inherent challenges,^15^ and we were unable to estimate differences in foodborne proportions by age, although such differences are likely to be important. Structured expert judgement studies can result in highly uncertain proportions of transmission attributed to food, reflecting uncertainty and variability in expert assessments and experts’ depth of knowledge. Despite WHO’s extensive recruitment effort, ensuring sufficient subregional coverage for the 221 hazard–subregion combinations remained challenging.

We showed that diarrhoeal enteric hazards transmitted by contaminated food remain an important burden globally, even with notable declines from 2000 to 2021. Policy makers and others committed to the universal availability of safe food can use this information alongside national estimates available from WHO for risk ranking in order to advocate for interventions to prevent and control foodborne diseases. In particular, continued attention is needed to reduce the high burden of foodborne diarrhoeal illness among young children and in low-income settings. By continuing to strengthen foodborne disease surveillance at a national level, countries can better inform further burden estimation efforts, evaluate impacts of disease interventions, track progress towards WHO food safety indicators, and ultimately increase the availability of safe food to all.

## Supplementary Material

Majowicz et al. supplementary files

## Figures and Tables

**Table:** Mean numbers of illnesses, deaths, and DALYs globally, from all routes of transmission and from foodborne transmission, by enteric disease hazard, 2021

	Illnesses*	Deaths*	DALYs	Proportion of illnesses that are foodborne	Foodborne illnesses	Foodborne deaths	Foodborne DALYs
*Campylobacter* †	291 000 000 (169 000 000 to 459 000 000)	71 200 (37 200 to 118 000)	4 260 000 (2 400 000 to 6 590 000)	0·510 (0·406 to 0·614)	148 000 000 (83 500 000 to 239 000 000)	35 400 (17 200 to 58 900)	2 150 000 (1 100 000 to 3 510 000)
*Cryptosporidium* spp	106 000 000 (57 400 000 to 169 000 000)	71 000 (40 700 to 112 000)	4 660 000 (2 710 000 to 7 190 000)	0·153 (0·082 to 0·236)	16 200 000 (6 800 000 to 28 300 000)	11 400 (4820 to 21 400)	769 000 (332 000 to 1 480 000)
*Cyclospora cayetanensis*	13 400 000 (−1 060 000 to 42 300 000)	19 300 (4420 to 58 300)	1 140 000 (276 000 to 3 050 000)	0·769 (0·572 to 0·919)	10 200 000 (−841 000 to 31 800 000)	14 600 (3290 to 44 000)	857 000 (192 000 to 2 290 000)
*Entamoeba histolytica*	23 400 000 (4 810 000 to 55 400 000)	25 100 (7260 to 58 300)	1 000 000 (327 000 to 2 180 000)	0·201 (0·054 to 0·454)	4 660 000 (618 000 to 15 400 000)	5020 (740 to 15 600)	183 000 (34 400 to 534 000)
Enteroaggregative *E coli*	16 600 000 (−175 000 000 to 191 000 000)	25 500 (−39 700 to 104 000)	1 430 000 (−2 230 000 to 5 670 000)	0·528 (0·411 to 0·633)	9 100 000 (−89 300 000 to 99 200 000)	13 000 (−21 500 to 53 200)	702 000 (−1 160 000 to 2 920 000)
Enteropathogenic *E coli*	49 300 000 (1 320 000 to 110 000 000)	17 800 (−2950 to 49 500)	1 050 000 (−135 000 to 2 820 000)	0·446 (0·340 to 0·573)	21 800 000 (592 000 to 49 300 000)	8260 (−1390 to 20 800)	540 000 (−74 600 to 1 340 000)
Enterotoxigenic *E coli*	260 000 000 (152 000 000 to 423 000 000)	55 300 (27 500 to 102 000)	3 170 000 (1 710 000 to 5 440 000)	0·505 (0·409 to 0·598)	131 000 000 (72 300 000 to 220 000 000)	28 900 (14 600 to 51 800)	1 790 000 (945 000 to 3 030 000)
*Giardia duodenalis*	321 000 000 (181 000 000 to 582 000 000)	0 (0 to 0)	232 000 (119 000 to 433 000)	0·086 (0·033 to 0·170)	28 000 000 (8 290 000 to 64 000 000)	0 (0 to 0)	20 300 (5330 to 48 000)
Norovirus	233 000 000 (155 000 000 to 332 000 000)	62 300 (36 700 to 95 400)	3 690 000 (2 240 000 to 5 570 000)	0·236 (0·127 to 0·368)	54 800 000 (28 000 000 to 97 100 000)	14 100 (5330 to 28 600)	809 000 (356 000 to 1 540 000)
Rotavirus	239 000 000 (169 000 000 to 339 000 000)	215 000 (162 000 to 275 000)	14 500 000 (11 300 000 to 18 400 000)	0·107 (0·037 to 0·211)	25 400 000 (7 990 000 to 50 500 000)	26 000 (6160 to 51700)	1 840 000 (392 000 to 3 600 000)
*Salmonella enterica*, non-typhoidal, diarrhoeal	35 000 000 (17 000 000 to 63 600 000)	35 800 (21 100 to 60 400)	1 930 000 (1 160 000 to 3 170 000)	0·665 (0·570 to 0·746)	23 300 000 (11 300 000 to 42 300 000)	23 600 (13 100 to 39 400)	1 270 000 (721 000 to 2 170 000)
Shiga toxin-producing *E coli*	92 400 000 (36 500 000 to 205 000 000)	36 900 (15 400 to 75 300)	2 240 000 (985 000 to 4 530 000)	0·562 (0·440 to 0·676)	51 900 000 (20 700 000 to 113 000 000)	20 500 (8630 to 42 000)	1 240 000 (546 000 to 2 410 000)
*Shigella* spp	426 000 000 (234 000 000 to 711 000 000)	152 000 (85 300 to 242 000)	7 990 000 (4 790 000 to 12 000 000)	0·278 (0·139 to 0·436)	118 000 000 (49 100 000 to 226 000 000)	42 500 (19 100 to 80 400)	2 250 000 (1 130 000 to 3 820 000)
*Vibrio cholerae*	99 800 000 (30 000 000 to 228 000 000)	94 100 (34 200 to 186 000)	3 580 000 (1 440 000 to 6 980 000)	0·228 (0·089 to 0·401)	23 000 000 (5 390 000 to 67 000 000)	21 400 (5800 to 57 900)	821 000 (252 000 to 1 970 000)
Total	2 210 000 000 (1 740 000 000 to 2 750 000 000)	881 000 (717 000 to 108 0000)	50 900 000 (42 500 000 to 60 100 000)	0·301 (0·247 to 0·354)	666 000 000 (483 000 000 to 884 000 000)	265 000 (196 000 to 351 000)	15 200 000 (11 600 000 to 19 100 000)

Values are mean (95% uncertainty interval), presented to three significant figures. Negative lower bounds in these estimates indicate considerable uncertainty around the attributable fractions used to derive these estimates, resulting from some diarrhoeal pathogens for which asymptomatic carriage by individuals is common or data are sparse; these negative lower bounds are shown to be transparent about this uncertainty. For hazards with negative point estimates for the population attributable fraction, indicating very weak confidence about the hazard causing disease (atypical enteropathogenic *Escherichia coli* [illnesses and deaths] and heat-labile toxin-producing enterotoxigenic *E coli* [deaths]), we opted not to include the associated burden.

* Incidence and mortality values for each hazard and overall, from all routes of transmission, are reproduced from Colston et al.^2^

† *Campylobacter jejuni, Campylobacter coli*, and other thermotolerant *Campylobacter* species.

## Data Availability

Data collected by WHO for this study are publicly available at https://www.who.int/teams/nutrition-and-food-safety/monitoring-nutritional-status-and-food-safety-and-events/foodborne-disease-estimates/2026-edition). The overall protocol and statistical analysis plan have been previously published.^9^ The analysis code is available via GitHub (https://github.com/fbdburden).

## Research in context

### Evidence before this study

In 2015, WHO published the first global burden of foodborne diseases estimates that showed that in 2010, diarrhoeal hazards transmitted via contaminated food resulted in 548 million illnesses, 200 000 deaths, and 15·8 million disability-adjusted life-years (DALYs) globally, with disproportionately high burden in low-income and middle-income countries (LMICs) and among children younger than 5 years. Global studies since then have estimated diarrhoeal disease burden from all routes of transmission with a focus on the burden avoidable through improved water, sanitation, and hygiene (WASH) and vaccination, or have estimated mortality in children younger than 5 years. In 2020, the 73rd World Health Assembly called for updated global burden of foodborne disease estimates to support countries in improving food safety systems, and in 2021 the WHO reconvened the Foodborne Disease Burden Epidemiology Reference Group to provide technical leadership on this task. From Sept 1, 2021 to Dec 15, 2022, we searched MEDLINE, PROSPERO, and healthdata.org to identify globally relevant estimates of the incidence, mortality, or burden caused by 14 diarrhoeal enteric disease hazards commonly transmitted by food (*Campylobacter jejuni, Campylobacter coli*, and other thermotolerant *Campylobacter* species; *Cryptosporidium* spp; *Cyclospora cayetanensis*; *Entamoeba histolytica*; enteroaggregative *Escherichia coli*; enteropathogenic *E coli*; enterotoxigenic *E coli*; *Giardia duodenalis*; norovirus; rotavirus; non-typhoidal *Salmonella enterica*; Shiga toxin-producing *E coli*; *Shigella* spp; and *Vibrio cholerae*). We searched by pathogen name, disease name (eg, campylobacteriosis), sequelae (eg, haemolytic-uraemic syndrome), and metric keywords (eg, incidence, burden, DALYs) and considered studies in all languages. Although many existing studies estimated the burden of individual foodborne diarrhoeal hazards as a cause of illness or death, we found no global estimates that integrated foodborne transmission and pathogen attribution in both high-income country and LMIC settings at a global scale and that included more than a single pathogen of a pathogen class. Thus, WHO commissioned a series of systematic reviews on the incidence, aetiology, sequelae, and case-fatality or mortality, to which we added data meeting review inclusion criteria identified during WHO country consultation with member states, as inputs for disease burden.

### Added value of this study

This data synthesis integrated systematic reviews, expert opinion, and available national data from WHO member states in a standardised manner over time, to provide trends by which to benchmark and monitor disease burden for these 14 foodborne hazards, to guide food safety interventions and to improve human health across the lifespan. These findings provide updated global and regional estimates, and the first national estimates for 194 countries, of the foodborne burden of diarrhoeal enteric hazards for the period 2000–21. In 2021, transmission of these 14 hazards by contaminated food resulted in 15·2 million DALYs. Globally, 64% of the foodborne DALY burden (9·75 million DALYs) was in children younger than 5 years, and 59% and 24% in the African and South-East Asia regions, respectively, while the highest DALY rates (per 100 000 population) were in the African and Eastern Mediterranean regions. Notably, 44% (6·78 million DALYs) of the global foodborne burden was concentrated in children younger than 5 years in the African region. *Campylobacter*, enterotoxigenic *E coli*, and *Shigella* spp were the leading causes of foodborne illnesses and deaths globally. Norovirus and Shiga toxin-producing *E coli* were also leading causes of foodborne illnesses, whereas rotavirus and non-typhoidal *S enterica* were leading causes of foodborne deaths. *Shigella* spp, *Campylobacter*, rotavirus, enterotoxigenic *E coli*, and non-typhoidal *S enterica* were the leading causes of foodborne DALYs globally. Despite declines over time, the current burden from foodborne transmission remains substantial.

### Implications of all the available evidence

The global burden of diarrhoeal diseases has decreased in the past 21 years, largely due to interventions that avert mortality. However, there remains a large burden of preventable and treatable disease borne largely by children younger than 5 years in LMICs. Transmission of diarrhoeal disease hazards via contaminated food is a major contributor to this burden. It is sufficient to warrant expanding global diarrhoeal disease prevention efforts beyond health care, treatments, WASH, and vaccination to also include improvements in the safety of the food supply, from food sources to processing, preparation, storage, and consumption.

## References

[1] Kirk MD, Pires SM, Black RE, World Health Organization estimates of the global and regional disease burden of 22 foodborne bacterial, protozoal, and viral diseases, 2010: a data synthesis. PLoS Med 2015; 12: e1001921. 26633831 10.1371/journal.pmed.1001921 PMC4668831

[2] Colston JM, Devleesschauwer B, Flynn T, Updated estimates of the global, regional and national burden, and etiology of diarrheal diseases transmissible via food: a systematic review and meta-analytical modelling study for the World Health Organization. medRxiv 2026; published online Jan 27. 10.64898/2026.01.26.26344508 (preprint).

[3] WHO. WHO estimates of the global burden of foodborne diseases: foodborne disease burden epidemiology reference group 2007–2015. Geneva: WHO, 2015. https://iris.who.int/server/api/core/bitstreams/2cd58abf-fa80-40ef-b98a-9d663f0a9c79/content (accessed Dec 28, 2025).

[4] Jaffee S, Henson S, Unnevehr L, Grace D, Cassou E. The safe food imperative: accelerating progress in low- and middle-income countries. International Bank for Reconstruction and Development, and The World Bank. 2019. 10.1596/978-1-4648-1345-0. (accessed Dec 28, 2025).

[5] UN. Seventy-third World Health Assembly: agenda item 15.3: strengthening efforts on food safety. Aug 3, 2020. https://apps.who.int/gb/ebwha/pdf_files/WHA73/A73_R5-en.pdf (accessed Dec 28, 2025).

[6] Lake RJ, Devleesschauwer B, Majowicz SE, WHO estimates of the global, regional, and national burden of 42 foodborne infectious and chemical hazards, 2000–21: an updated data synthesis. Lancet Glob Health 2026; published online June 3. 10.1016/j.langlo.2026.103994. PMC13433341 42235567

[7] WHO. WHO global strategy for food safety 2022–2030: towards stronger food safety systems and global cooperation. Geneva: WHO, 2022. https://iris.who.int/server/api/core/bitstreams/d7d3a517-f794-4d25-9ebf-02a7e6a01f42/content (accessed Dec 28, 2025).

[8] WHO. A global health strategy for 2025–2028: advancing equity and resilience in a turbulent world. Geneva: WHO, 2025. https://www.who.int/publications/i/item/9789240101012 (accessed Dec 19, 2025).

[9] Devleesschauwer D, Vaes L, Fernandez K, Computational framework for the World Health Organization estimates of the global, regional and national burden of foodborne diseases 2026 edition. medRxiv 2026; published online May 17. 10.64898/2026.05.13.26353030 (preprint).

[10] Majowicz SE, Scallan Walter E, Pires SM, WHO estimates of the global, regional, and national burden of eight foodborne non-diarrhoeal enteric disease hazards, 2000–21: an updated data synthesis, Lancet Glob Health 2026, published online June 15. 10.1016/j.langlo.2026.103981. 42296980

[11] Stevens GA, Alkema L, Black RE, , and The GATHER Working Group. Guidelines for Accurate and Transparent Health Estimates Reporting: the GATHER statement. Lancet 2016; 388: e19–23. 27371184 10.1016/S0140-6736(16)30388-9

[12] Devleesschauwer B, Charalampous P, Gorasso V, Standardised reporting of burden of disease studies: the STROBOD statement. Popul Health Metr 2024; 22: 28. 39375690 10.1186/s12963-024-00347-9 PMC11459887

[13] Scallan Walter EJ, Griffin PM, Bruce BB, Hoekstra RM. Estimating the number of illnesses caused by agents transmitted commonly through food: a scoping review. Foodborne Pathog Dis 2021; 18: 841–58. 34529512 10.1089/fpd.2021.0038

[14] Colston JM, Flynn TG, Denton AH, Updating global estimates of pathogen-attributable diarrhoeal disease burden: a methodology and integrated protocol for a broad-scope systematic review of a syndrome with diverse infectious aetiologies. BMJ Open 2025; 15: e093018. 10.1136/bmjopen-2024-093018 PMC11969593 40180367

[15] Pires S, Mughini-Gras L, Hoffmann S, World Health Organization attribution of burden of foodborne diseases to transmission pathways and specific foods. Research Square 2026; published online April 22. 10.21203/rs.3.rs-9449162/v1 (preprint). 42705735

[16] WHO. The Global Health Observatory: foodborne disease estimates. Geneva: WHO, 2026. https://www.who.int/data/gho/data/themes/topics/foodborne-diseases-estimates (accessed Dec 19, 2025).

[17] WHO. WHO Foodborne Disease Estimates 2026 Edition, 2026, WHO; Geneva. https://www.who.int/teams/nutrition-and-food-safety/monitoring-nutritional-status-and-food-safety-and-events/foodborne-disease-estimates/2026-edition (accessed May 26, 2026).

[18] GBD 2021 Diseases and Injuries Collaborators. Global incidence, prevalence, years lived with disability (YLDs), disability-adjusted life-years (DALYs), and healthy life expectancy (HALE) for 371 diseases and injuries in 204 countries and territories and 811 subnational locations, 1990–2021: a systematic analysis for the Global Burden of Disease Study 2021. Lancet 2024; 403: 2133–61. 38642570 10.1016/S0140-6736(24)00757-8 PMC11122111

[19] Aliabadi N, Antoni S, Mwenda JM, Global impact of rotavirus vaccine introduction on rotavirus hospitalisations among children under 5 years of age, 2008–16: findings from the Global Rotavirus Surveillance Network. Lancet Glob Health 2019; 7: e893–903. 31200889 10.1016/S2214-109X(19)30207-4 PMC7336990

[20] Scallan Walter EJ, Cui Z, Tierney R, Foodborne illness acquired in the United States—major pathogens, 2019. Emerg Infect Dis 2025; 31: 669–77. 40133035 10.3201/eid3104.240913 PMC11950263

[21] GBD 2021 Diarrhoeal Diseases Collaborators. Global, regional, and national age-sex-specific burden of diarrhoeal diseases, their risk factors, and aetiologies, 1990–2021, for 204 countries and territories: a systematic analysis for the Global Burden of Disease Study 2021. Lancet Infect Dis 2025; 25: 519–36. 39708822 10.1016/S1473-3099(24)00691-1 PMC12018300

[22] Black RE, Perin J, Yeung D, Estimated global and regional causes of deaths from diarrhoea in children younger than 5 years during 2000–21: a systematic review and Bayesian multinomial analysis. Lancet Glob Health 2024; 12: e919–28. 38648812 10.1016/S2214-109X(24)00078-0 PMC11099298

[23] Sears A, Baker MG, Wilson N, Marked campylobacteriosis decline after interventions aimed at poultry, New Zealand. Emerg Infect Dis 2011; 17: 1007–15. 21749761 10.3201/eid1706.101272 PMC3358198

[24] Gonzales BL, Ho-Palma AC, Andrade DA, *Campylobacter* spp. in chicken meat from traditional markets in Peru and its impact measured through a quantitative microbiological risk assessment. Food Res Int 2025; 200: 115424. 39779164 10.1016/j.foodres.2024.115424 PMC12371221

[25] van der Vossen-Wijmenga WP, den Besten HMW, Hazeleger WC, Zwietering MH. *Campylobacter* in the domestic kitchen: linking human and microbiological behaviour. Int J Food Microbiol 2025; 441: 111270. 40561858 10.1016/j.ijfoodmicro.2025.111270

[26] Poh CJ, Rodwell EV, Greig DR, Nair S, Chattaway MA, Jenkins C. Microbiology and epidemiology of enteroaggregative *Escherichia coli* isolated from UK residents in England, 2016–2023: what are the risks to public health? J Med Microbiol 2025; 74: 002097. 41231719 10.1099/jmm.0.002097 PMC12614372

[27] Farahmand M, Khales P, Salavatiha Z, Worldwide prevalence and genotype distribution of human astrovirus in gastroenteritis patients: a systematic review and meta-analysis. Microb Pathog 2023; 181: 106209. 37385570 10.1016/j.micpath.2023.106209

[28] Troeger C, Colombara DV, Rao PC, Global disability-adjusted life-year estimates of long-term health burden and undernutrition attributable to diarrhoeal diseases in children younger than 5 years. Lancet Glob Health 2018; 6: e255–69. 29433665 10.1016/S2214-109X(18)30045-7 PMC5861379

[29] Cama VA, Bern C, Roberts J, *Cryptosporidium* species and subtypes and clinical manifestations in children, Peru. Emerg Infect Dis 2008; 14: 1567–74. 18826821 10.3201/eid1410.071273 PMC2609889

[30] Ishaque T, Islam MB, Ara G, High mortality from Guillain-Barré syndrome in Bangladesh. J Peripher Nerv Syst 2017; 22: 121–26. 28447405 10.1111/jns.12215

