# Supplementary material for: WHO estimates of the global, regional, and national burden of 14 foodborne diarrhoeal enteric hazards, 2000–21: an updated data synthesis
Source: Lancet Glob Health. Author manuscript; Available in PMC 2026 Jul 14. (PMC13358865; doi:10.1016/j.langlo.2026.103997)
Supplement: Majowicz et al. supplementary files [file NIHMS2190867-supplement-Majowicz_et_al__supplementary_files.zip › 1-s2.0-S2214109X26001592-mmc1.pdf]

THE LANCET
Global Health

Supplementary appendix 1

This appendix formed part of the original submission and has been peer reviewed. We post it as supplied by the authors.

Supplement to: Majowicz SE, Colston JM, Kirk MD, et al. WHO estimates of the global, regional, and national burden of 14 foodborne diarrhoeal enteric hazards, 2000–21: an updated data synthesis. *Lancet Glob Health* 2026. https://doi.org/10.1016/j.langlo.2026.103997

Table of Contents

ADDITIONAL METHODS **2**

Incidence and Mortality Estimation **2**
Subregion A Countries 2
Other Countries 2

Data Cleaning **3**

DATA AND ASSUMPTIONS **4**

Diarrhoeal disease hazards **4**
***Campylobacter* spp.** 4
***Cryptosporidium* spp.** 6
Cyclospora cayetanensis 8
Entamoeba histolytica 10
Enteroaggregative *E. coli* 11
Enteropathogenic *E. coli* 13
Enterotoxigenic *E. coli* 14
Giardia duodenalis 16
Norovirus 17
Rotavirus 20
***Salmonella* spp. (non-typhoidal)** 21
Shiga toxin-producing *E. coli* 24
***Shigella* spp.** 26
Vibrio cholerae 28

REFERENCES **30**

SUPPLEMENTARY TABLES AND FIGURES **33**

GATHER AND STROBOD CHECKLISTS **61**

ADDITIONAL METHODS

Incidence and Mortality Estimation

Subregion A Countries

For countries in subregion A* we used national-level data from a World Health Organization (WHO)-commissioned update of a scoping review of studies published during January 1, 1995, to December 31, 2023, that estimated the population-level incidence or mortality of ≥1 of the 14 diarrhoeal disease hazards for any country in this grouping.[1,2,3] Additional eligible data meeting the scoping review's inclusion criteria, identified during internal review and country consultation, were added (detailed below). In total, 37 studies were included: 35 for illnesses representing 42 sets of estimates from 15 countries; and 23 for deaths representing 24 sets of estimates from 10 countries.

A full description of the meta-analysis modelling methods used to derive incidence and mortality estimates, including specific model details by hazard, are provided elsewhere.[3,4]

***Subregion A countries:**

Region of the Americas (AMR) A: Antigua and Barbuda, Bahamas, Barbados, Canada, Chile, Guyana, Panama, Saint Kitts and Nevis, Trinidad and Tobago, United States of America, Uruguay

European Region (EUR) A: Andorra, Austria, Belgium, Croatia, Cyprus, Czechia, Denmark, Estonia, Finland, France, Germany, Greece, Hungary, Iceland, Ireland, Israel, Italy, Latvia, Lithuania, Luxembourg, Malta, Monaco, Netherlands (Kingdom of the), Norway, Poland, Portugal, Romania, San Marino, Slovakia, Slovenia, Spain, Sweden, Switzerland, United Kingdom of Great Britain and Northern Ireland

Eastern Mediterranean Region (EMR) A: Bahrain, Kuwait, Oman, Qatar, Saudi Arabia, United Arab Emirates

Western Pacific Region (WPR) A: Australia, Brunei Darussalam, Cook Islands, Japan, Nauru, New Zealand, Niue, Republic of Korea, Singapore

Other Countries

For all other countries, we used data from a WHO-commissioned systematic review (PROSPERO: CRD42023427998) and meta-analysis of the incidence and mortality of diarrhoea due to the 14 diarrhoeal disease hazards.[2,3]

Hazard-specific incidence and mortality were estimated by first modeling aetiology-specific attributable fractions, to estimate the proportion of diarrhoea cases and deaths attributable to each hazard.[3] Models were fitted to data sourced and extracted from publications identified by the systematic review according to pre-specified inclusion and exclusion criteria and methods reported elsewhere.[2,3,4] Briefly, eligible sources were publications reporting prevalence of ≥2 of the

14 hazards diagnosed in subjects enrolled in health facility- and community-based diarrhoea aetiology studies, carried out in these countries.[2,3]

All hazard-specific aetiology proportion models adjusted for rotavirus vaccination status by country, used molecular detection methods as the referent group, and adjusted for asymptomatic carriage as follows.[3] For the majority of diarrhoeal pathogens, aetiology proportions were estimated as attributable fractions (AFs) adjusting for asymptomatic carriage. For *Campylobacter* spp., the aetiology proportion was derived by averaging the AF with the fraction among symptomatic individuals. For *Giardia duodenalis* and Shiga toxin-producing *E. coli* (STEC), values adjusted for asymptomatic carriage were negative, and aetiology fractions were derived by averaging the fraction estimated from symptomatic individuals with zero.

We then applied these model-derived AFs to draw-level diarrhoeal incidence estimates from the Global Burden of Disease Study (GBD) 2021 obtained directly from the Institute for Health Metrics and Evaluation (IHME; see Acknowledgements), and draw-level diarrhoeal mortality estimates from WHO's Global Health Estimates (see Acknowledgements), stratified by age, sex, country, and year, for the years 2000 to 2021. Incidence estimates were calculated using AFs in community-detected and outpatient attended diarrhoea cases, whereas mortality estimates used AFs in inpatient (i.e., hospitalized) cases.[3]

Data Cleaning

General data cleaning was done for each hazard.[4] In a first check, incomplete data points were flagged and send back to the systematic reviewer teams to address if possible. After review, incomplete data points were excluded. Additional exclusions were done for overseas and disputed territories (i.e., locations other than the 194 WHO Member States), data before 1990, and data that were missing WHO region, sample size, or model parameters. Where possible, missing sample sizes were imputed with the national population. For extensive systematic reviews, only the most aggregated data point of a study was kept. For this we looked at age, sex, and sub-national regions and subpopulations. Additional ad hoc decisions regarding outliers or limited data regions were also undertaken.

DATA AND ASSUMPTIONS

Diarrhoeal disease hazards

Campylobacter spp.

HAZARD	***Campylobacter jejuni, coli,*** **and other thermotolerant species**
**INCIDENCE**	Subregion A countries:<br>We used 27 data points from 21 studies estimating the national, population-level incidence of *Campylobacter* diarrhoea from a WHO-commissioned update of a scoping review,[1,3] from the following 14 countries (data point by year of study publication, see reference 3 for references): Australia (2005, 2014, 2023), Barbados (2013), Canada (2006, 2013), Denmark (2013, 2020, 2022), France (2015, 2017), Germany (2013), Italy (2013), Japan (2008, 2011), Netherlands (Kingdom of the) (2012, 2013), New Zealand (2000), Poland (2013), Sweden (2013, 2018), United Kingdom of Great Britain and Northern Ireland (1999, 2012, 2013), United States of America (2009, 2011, 2011). To this, we added 7 additional data points from 4 studies identified during the internal FERG review and WHO country consultation period that met the scoping review's inclusion criteria in the following 4 countries: Netherlands (Kingdom of the) (2017, 2024), New Zealand (2011, 2014 [2 data points from 2011 and 2013]), Switzerland (2024) and United States of America (2025) (for a total of 34 data points from 25 studies in 15 countries). Data points included the total estimated number of illnesses (median, mean) or the incidence per population, with some measure of uncertainty (credible interval, confidence interval), when available.<br><br>All other countries:<br>We used 204 data points from 75 out-patient and community-based studies on *Campylobacter* prevalence among diarrhoeal cases, from 40 countries published between 1993 to 2023 (see reference 3 for countries and references).<br><br>No additional data meeting the inclusion criteria were identified during the internal FERG review and WHO country consultation period.
**CLINICAL OUTCOMES**	Clinical outcomes were acute *Campylobacter* diarrhoea (severe), acute *Campylobacter* diarrhoea (moderate), acute *Campylobacter* diarrhoea (mild), Guillain-Barré Syndrome (GBS) due to *Campylobacter* infection, and death following diarrhoea and GBS.<br><br>We assumed that 2% of *Campylobacter* diarrhoeal cases result in severe diarrhoea, 25% of *Campylobacter* diarrhoeal cases result in moderate diarrhoea, and 73% of *Campylobacter* diarrhoeal cases result in mild diarrhoea.[5]<br><br>To estimate the number of GBS cases following *Campylobacter*, we used GBS incidence estimates from GBD 2021, stratified by age, sex, country, and year, minus the incidence due to COVID, obtained directly from IHME. We multiplied GBS incidence by a global estimate of the proportion of GBS following *Campylobacter* from a prior systematic review,[6] that estimated 31% (minimum 28% - maximum 45%) of GBS cases were due to Campylobacter infection, consistent with new evidence.[7,8]

**DURATION**	In children 0-4 years of age, we assumed the duration of severe diarrhoea was 8.4 days, moderate diarrhoea was 6.4 days, and mild diarrhoea was 4.3 days.[5] In persons ≥5 years of age, we assumed the duration of *Campylobacter* diarrhoea was 2.8 days.[5]<br><br>We assumed the duration of GBS following *Campylobacter* infection is life-long.[5]
**DISABILITY WEIGHT**	We used GBD 2019 disability weights, from Salomon et al,[9] as follows.<br><br>For acute *Campylobacter* diarrhoea (severe), we used the GBD disability weight for the "diarrhoea, severe" ("has diarrhoea three or more times a day with severe belly cramps. The person is very thirsty and feels nauseous and tired"). Disability weight: 0.247 (Uncertainty interval [UI]: 0.164 to 0.348).<br><br>For acute *Campylobacter* diarrhoea (moderate), we used the GBD disability weight for "diarrhoea, moderate" ("has diarrhoea three or more times a day, with painful cramps in the belly and feeling thirsty"). Disability weight: 0.188 (UI: 0.125 to 0.264).<br><br>For acute *Campylobacter* diarrhoea (mild), we used the GBD disability weight for "diarrhoea, mild" ("has diarrhoea three or more times a day with occasional discomfort in the belly"). Disability weight: 0.074 (UI: 0.049 to 0.104).<br><br>For GBS following *Campylobacter spp.*, we used the GBD disability weight for "spinal cord lesion below neck level (treated)" ("is paralyzed from the waist down, cannot feel or move the legs and has difficulties with urine and bowel control. The person uses a wheelchair to move around"), which is the health state the GBD study uses for GBS due to diarrhoeal diseases. Disability weight: 0.296 (UI: 0.198 to 0.414).
**MORTALITY**	<u>Subregion A countries:</u><br>We used 12 data points from 12 studies estimating the national, population-level mortality due to *Campylobacter* diarrhoea from a WHO-commissioned update of a scoping review,[1,3] from the following 9 countries (data point by year of study publication, see reference 3 for references): Australia (2014, 2023), Canada (2015), Denmark (2020, 2022), France (2017), Netherlands (Kingdom of the) (2012), Sweden (2018), New Zealand (2000), United Kingdom of Great Britain and Northern Ireland (2020), United States of America (1999, 2011). To this, we added 7 additional data points from 6 studies identified during the internal FERG review and WHO country consultation period that met the scoping review's inclusion from the following 4 countries: Netherlands (Kingdom of the) (2017, 2024), New Zealand (2011, 2014 [two data points from 2011 and 2013]), Switzerland (2024), and United States of America (2025) (for a total of 19 data points from 18 studies in 10 countries). Data points included the total estimated number of deaths (median, mean) or the incidence per population, with some measure of uncertainty (credible interval, confidence interval), when available.<br><br><u>All other countries:</u><br>We used 124 data points from 32 in-patient studies on *Campylobacter* prevalence among diarrhoeal cases, from 28 countries published between 2006-2023 (see reference 3 for countries and references).<br><br>No additional data meeting the inclusion criteria were identified during the internal FERG review and WHO country consultation period.

	<u>All countries:</u><br>We assumed that the case fatality ratio for GBS following *Campylobacter* infection was 4.1% (minimum 2.4% - maximum 6%).[5]
**AGE DISTRIBUTION**	For diarrhoeal cases, in subregion A countries we used age distributions for *Campylobacter* cases from the US Centers for Disease Control and Prevention (CDC) FoodNet surveillance reports for the years 2000, 2010, and 2021.[10-12] We assumed the same age distribution for cases and deaths.<br><br>For all other countries, we used the country- and year-specific age distribution of cases and of deaths from GBD 2021 diarrhoea estimates, for *Campylobacter* cases and deaths, respectively.<br><br>We assumed the age distribution of *Campylobacter*-associated GBS cases and deaths were the same as *Campylobacter* diarrhoea cases and deaths.
**SEX DISTRIBUTION**	For subregion A countries, we assumed 50% of *Campylobacter* cases, GBS cases, and deaths were male.[5]<br><br>For all other countries, we used the country- and year-specific sex distribution of diarrhoeal cases and of deaths from GBD 2021 diarrhoea estimates, for *Campylobacter* and GBS cases and deaths, respectively.
**GEOGRAPHIC DISTRIBUTION**	Present worldwide.

Cryptosporidium spp.

**HAZARD**	***Cryptosporidium* spp.**
**INCIDENCE**	<u>Subregion A countries:</u><br>We used 12 data points from 10 studies estimating the national, population-level incidence of *Cryptosporidium* diarrhoea from a WHO-commissioned update of a scoping review,[1,3] from the following 6 countries (data point by year of study publication, see reference 3 for references): Australia (2005, 2014), Canada (2013), Germany (2013), Netherlands (Kingdom of the) (2012, 2013), United Kingdom of Great Britain and Northern Ireland (1999, 2012, 2013) and United States of America (1999, 2011, 2011). To this, we added 3 additional data points from 3 studies identified during the internal FERG review and WHO country consultation period that met the scoping review's inclusion criteria in the following 2 countries: Netherlands (Kingdom of the) (2017, 2024) and New Zealand (2011) (for a total of 15 data points from 13 studies in 7 countries). Data points included the total estimated number of illnesses (median, mean) or the incidence per population, with some measure of uncertainty (credible interval, confidence interval), when available.<br><br><u>All other countries:</u><br>We used 191 data points from 62 out-patient and community-based studies on *Cryptosporidium* prevalence among diarrhoeal cases, from 36 countries published between 1993 to 2023 (see reference 3 for countries and references).

	No additional data meeting the inclusion criteria were identified during the internal FERG review and WHO country consultation period.
**CLINICAL OUTCOMES**	Clinical outcomes were acute *Cryptosporidium* diarrhoea (severe), acute *Cryptosporidium* diarrhoea (moderate), acute *Cryptosporidium* diarrhoea (mild), and death following diarrhoea.<br><br>We assumed that 0.5% of *Cryptosporidium* diarrhoeal cases result in severe diarrhoea, 8.5% of *Cryptosporidium* diarrhoeal cases result in moderate diarrhoea, and 91% of *Cryptosporidium* diarrhoeal cases result in mild diarrhoea.[5]
**DURATION**	In children 0-4 years of age, we assumed the duration of severe diarrhoea was 8.4 days, moderate diarrhoea was 6.4 days, and mild diarrhoea was 4.3 days.[5] In persons ≥5 years of age, we assumed the duration of *Cryptosporidium* diarrhoea was 2.8 days.[5]
**DISABILITY WEIGHT**	We used GBD 2019 disability weights, from Salomon et al,[9] as follows.<br><br>For acute *Cryptosporidium* diarrhoea (severe), we used the GBD disability weight for the "diarrhoea, severe" ("has diarrhoea three or more times a day with severe belly cramps. The person is very thirsty and feels nauseous and tired"). Disability weight: 0.247 (UI: 0.164 to 0.348).<br><br>For acute *Cryptosporidium* diarrhoea (moderate), we used the GBD disability weight for "diarrhoea, moderate" ("has diarrhoea three or more times a day, with painful cramps in the belly and feeling thirsty"). Disability weight: 0.188 (UI: 0.125 to 0.264).<br><br>For acute *Cryptosporidium* diarrhoea (mild), we used the GBD disability weight for "diarrhoea, mild" ("has diarrhoea three or more times a day with occasional discomfort in the belly"). Disability weight: 0.074 (UI: 0.049 to 0.104).
**MORTALITY**	<u>Subregion A countries:</u><br>We used 6 data points from 6 studies estimating the national, population-level mortality due to *Cryptosporidium* diarrhoea from a WHO-commissioned update of a scoping review,[1,3[mansucript]] from the following 5 countries (data point by year of study publication, see reference 3 for references): Australia (2014), Canada (2015), Netherlands (Kingdom of the) (2012), United Kingdom of Great Britain and Northern Ireland (2020), United States of America (1999, 2011). To this, we added 3 additional data points from 3 studies identified during the internal FERG review and WHO country consultation period that met the scoping review's inclusion criteria in the following 2 countries: Netherlands (Kingdom of the) (2017, 2024) and New Zealand (2011) (for a total of 9 data points from 9 studies in 6 countries). Data points included the total estimated number of deaths (median, mean) or the incidence per population, with some measure of uncertainty (credible interval, confidence interval), when available.<br><br><u>All other countries:</u><br>We used 104 data points from 20 in-patient studies on *Cryptosporidium* prevalence among diarrhoeal cases from 17 countries published between 2002 to 2023 (see reference 3 for countries and references).<br><br>No additional data meeting the inclusion criteria were identified during the internal FERG review and WHO country consultation period.

**AGE DISTRIBUTION**	For subregion A countries, we used age distributions for *Cryptosporidium* cases from the US Centers for Disease Control and Prevention (CDC) FoodNet surveillance reports for the years 2000, 2010, and 2021,[10-12] We assumed the same age distribution for cases and deaths.<br><br>For all other countries, we used the country- and year-specific age distribution of cases and of deaths from GBD 2021 diarrhoea estimates, for C*ryptosporidium* cases and deaths, respectively.
**SEX DISTRIBUTION**	For subregion A countries, we assumed 50% of *Cryptosporidium* cases and deaths were male.[5]<br><br>For all other countries, we used the country- and year-specific sex distribution of diarrhoeal cases and of deaths from GBD 2021 diarrhoea estimates, for *Cryptosporidium* cases and deaths, respectively.
**GEOGRAPHIC DISTRIBUTION**	Present worldwide.

Cyclospora cayetanensis

**HAZARD**	***Cyclospora cayetanensis***
**INCIDENCE**	<u>Subregion A countries:</u><br>We used 3 data points from 3 studies estimating the national, population-level incidence of *Cyclospora* diarrhoea from a WHO-commissioned update of a scoping review,[1,3] from the following 2 countries (data point by year of study publication, see reference 3 for references): Canada (2013) and United States of America (1999, 2011). No additional data meeting the inclusion criteria were identified during the FERG internal review and WHO country consultation period. Data points included the total estimated number of illnesses (median, mean) or the incidence per population, with some measure of uncertainty (credible interval, confidence interval), when available.<br><br><u>All other countries:</u><br>We used 75 data points from 13 out-patient and community-based studies on *Cyclospora* prevalence among diarrhoeal cases, from 18 countries published between 1999 to 2023 (see reference 3 for countries and references).<br><br>No additional data meeting the inclusion criteria were identified during the internal FERG review and WHO country consultation period.
**CLINICAL OUTCOMES**	Clinical outcomes were acute *Cyclospora* diarrhoea (severe), acute *Cyclospora* diarrhoea (moderate), acute *Cyclospora* diarrhoea (mild), and death following diarrhoea.<br><br>We assumed that 0.5% of *Cyclospora* diarrhoeal cases result in severe diarrhoea, 8.5% of *Cyclospora* diarrhoeal cases result in moderate diarrhoea, and 91% of *Cyclospora* diarrhoeal cases result in mild diarrhoea.[5]

**DURATION**	In children 0-4 years of age, we assumed the duration of severe diarrhoea was 8.4 days, moderate diarrhoea was 6.4 days, and mild diarrhoea was 4.3 days.[5] In persons ≥5 years of age, we assumed the duration of *Cyclospora* diarrhoea was 2.8 days.[5]
**DISABILITY WEIGHT**	We used GBD 2019 disability weights, from Salomon et al,[9] as follows.<br><br>For acute *Cyclospora* diarrhoea (severe), we used the GBD disability weight for the "diarrhoea, severe" ("has diarrhoea three or more times a day with severe belly cramps. The person is very thirsty and feels nauseous and tired"). Disability weight: 0.247 (UI: 0.164 to 0.348).<br><br>For acute *Cyclospora* diarrhoea (moderate), we used the GBD disability weight for "diarrhoea, moderate" ("has diarrhoea three or more times a day, with painful cramps in the belly and feeling thirsty"). Disability weight: 0.188 (UI: 0.125 to 0.264).<br><br>For acute *Cyclospora* diarrhoea (mild), we used the GBD disability weight for "diarrhoea, mild" ("has diarrhoea three or more times a day with occasional discomfort in the belly"). Disability weight: 0.074 (UI: 0.049 to 0.104).
**MORTALITY**	Subregion A countries:<br>We estimated zero mortality using 3 estimates from 3 national, population-level studies of the mortality due to *Cyclospora* from the following 2 countries: Canada (2015) and United States of America (1999 and 2011) (data point by year of study publication, see reference 3 for references), identified from a WHO-commissioned update of a scoping review.[1,3] No additional data meeting the inclusion criteria were identified during the FERG internal review and WHO country consultation period. Data points included the total estimated number of deaths (median, mean) or the incidence per population, with some measure of uncertainty (credible interval, confidence interval), when available.<br><br>All other countries:<br>We used 39 data points from 5 in-patient studies on *Cyclospora* prevalence among diarrhoeal cases, from 10 countries published between 2016 to 2023 (see reference 3 for countries and references).<br><br>No additional data meeting the inclusion criteria were identified during the internal FERG review and WHO country consultation period.
**AGE DISTRIBUTION**	For subregion A countries, we used age distributions for *Cyclospora* cases from the US Centers for Disease Control and Prevention (CDC) FoodNet surveillance reports for the years 2000, 2010, and 2021.[10-12] We assumed the same age distribution for cases and deaths.<br><br>For all other countries, we used the country- and year-specific age distribution of cases and of deaths from GBD 2021 diarrhoea estimates, for *Cyclospora* cases and deaths, respectively.
**SEX DISTRIBUTION**	For subregion A countries, we assumed 50% of *Cyclospora* cases and deaths were male.[5]

	For all other countries, we used the country- and year-specific sex distribution of diarrhoeal cases and of deaths from GBD 2021 diarrhoea estimates, for *Cyclospora* cases and deaths, respectively.
**GEOGRAPHIC DISTRIBUTION**	Present worldwide.

Entamoeba histolytica

**HAZARD**	***Entamoeba histolytica***
**INCIDENCE**	Subregion A countries:<br>We used 1 data point from 1 study estimating the national, population-level incidence of *Entamoeba* diarrhoea from a WHO-commissioned update of a scoping review,[1,3] from the Netherlands (Kingdom of the) (2001; see reference 3 for reference). An estimate of 1 episode of illness per 2,340 person-years was used to estimate the symptomatic incidence rate. No additional data meeting the inclusion criteria were identified during the FERG internal review and WHO country consultation period.<br><br>All other countries:<br>We used 160 data points from 51 out-patient and community-based studies on Entamoeba prevalence among diarrhoeal cases from 34 countries published between 1996 to 2023 (see reference 3 for countries and references).<br><br>No additional data meeting the inclusion criteria were identified during the internal FERG review and WHO country consultation period.
**CLINICAL OUTCOMES**	Clinical outcomes were acute *Entamoeba* diarrhoea (severe), acute *Entamoeba* diarrhoea (moderate), acute *Entamoeba* diarrhoea (mild), and death following diarrhoea. Due to lack of robust data, we were unable to include the outcome amoebic liver abscess.<br><br>We assumed that 0.5% of *Entamoeba* diarrhoeal cases result in severe diarrhoea, 8.5% of *Entamoeba* diarrhoeal cases result in moderate diarrhoea, and 91% of *Entamoeba* diarrhoeal cases result in mild diarrhoea.[5]
**DURATION**	In children 0-4 years of age, we assumed the duration of severe diarrhoea was 8.4 days, moderate diarrhoea was 6.4 days, and mild diarrhoea was 4.3 days.[5] In persons ≥5 years of age, we assumed the duration of *Entamoeba* diarrhoea was 2.8 days.[5]
**DISABILITY WEIGHT**	We used GBD 2019 disability weights, from Salomon et al,[9] as follows.<br><br>For acute *Entamoeba* diarrhoea (severe), we used the GBD disability weight for the "diarrhoea, severe" ("has diarrhoea three or more times a day with severe belly cramps. The person is very thirsty and feels nauseous and tired"). Disability weight: 0.247 (UI: 0.164 to 0.348).<br><br>For acute *Entamoeba* diarrhoea (moderate), we used the GBD disability weight for "diarrhoea, moderate" ("has diarrhoea three or more times a day, with painful cramps in the belly and feeling thirsty"). Disability weight: 0.188 (UI: 0.125 to 0.264).

	For acute *Entamoeba* diarrhoea (mild), we used the GBD disability weight for "diarrhoea, mild" ("has diarrhoea three or more times a day with occasional discomfort in the belly"). Disability weight: 0.074 (UI: 0.049 to 0.104).
**MORTALITY**	Subregion A countries:<br>We were unable to identify national, population-level studies of the mortality due to *Entamoeba*, including via the WHO-commissioned update of a scoping review,[1,3] Thus, we assumed zero mortality.<br><br>All other countries:<br>We used 86 data points from 15 in-patient studies on *Entamoeba* prevalence among diarrhoeal cases, from 16 countries published between 2005 to 2022 (see reference 3 for countries and references).<br><br>No additional data meeting the inclusion criteria were identified during the internal FERG review and WHO country consultation period.
**AGE DISTRIBUTION**	For subregion A countries, we used age distributions for *Entamoeba* cases from national surveillance from Taiwan for the years 2011 and 2020.[13] We assumed the same age distribution for cases and deaths.<br><br>For all other countries, we used the country- and year-specific age distribution of cases and of deaths from GBD 2021 diarrhoea estimates, for *Entamoeba* cases and deaths, respectively.
**SEX DISTRIBUTION**	For subregion A countries, we assumed 50% of *Entamoeba* cases and deaths were male.[5]<br><br>For all other countries, we used the country- and year-specific sex distribution of diarrhoeal cases and of deaths from GBD 2021 diarrhoea estimates, for *Entamoeba* cases and deaths, respectively.
**GEOGRAPHIC DISTRIBUTION**	Present worldwide.

Enteroaggregative *E. coli*

**HAZARD**	**Enteroaggregative *Escherichia coli* (EAEC)**
**INCIDENCE**	Subregion A countries:<br>We initially intended to use estimates from national, population-level studies of the incidence of EAEC diarrhoea identified via the scoping review,[1,3] However, given there were only two older estimates from one country (United Kingdom of Great Britain and Northern Ireland, in 1999 and 2012),[14,15] and given feedback to WHO during their country consultation process that these estimates were orders of magnitude too large for current circumstances, we determined there were not enough data to generate reliable estimates of EAEC incidence, mortality, and burden in subregion A countries.<br><br>All other countries:

	We used 179 data points from 60 out-patient and community-based studies on EAEC prevalence among diarrhoeal cases from 33 countries published between 1999 to 2023 (see reference 3 for countries and references).<br><br>No additional data meeting the inclusion criteria were identified during the internal FERG review and WHO country consultation period.
**CLINICAL OUTCOMES**	Clinical outcomes were acute EAEC diarrhoea (severe), acute EAEC diarrhoea (moderate), acute EAEC diarrhoea (mild), and death following diarrhoea.<br><br>We assumed that 0.5% of EAEC diarrhoeal cases result in severe diarrhoea, 8.5% of EAEC diarrhoeal cases result in moderate diarrhoea, and 91% of EAEC diarrhoeal cases result in mild diarrhoea.[5]
**DURATION**	In children 0-4 years of age, we assumed the duration of severe diarrhoea was 8.4 days, moderate diarrhoea was 6.4 days, and mild diarrhoea was 4.3 days.[5] In persons ≥5 years of age, we assumed the duration of EAEC diarrhoea was 2.8 days.[5]
**DISABILITY WEIGHT**	We used GBD 2019 disability weights, from Salomon et al,[9] as follows.<br><br>For acute EAEC diarrhoea (severe), we used the GBD disability weight for the "diarrhoea, severe" ("has diarrhoea three or more times a day with severe belly cramps. The person is very thirsty and feels nauseous and tired"). Disability weight: 0.247 (UI: 0.164 to 0.348).<br><br>For acute EAEC diarrhoea (moderate), we used the GBD disability weight for "diarrhoea, moderate" ("has diarrhoea three or more times a day, with painful cramps in the belly and feeling thirsty"). Disability weight: 0.188 (UI: 0.125 to 0.264).<br><br>For acute EAEC diarrhoea (mild), we used the GBD disability weight for "diarrhoea, mild" ("has diarrhoea three or more times a day with occasional discomfort in the belly"). Disability weight: 0.074 (UI: 0.049 to 0.104).
**MORTALITY**	Subregion A countries:<br>We did not estimate mortality, as described above for incidence.<br><br>All other countries:<br>We used 97 data points from 25 in-patient studies on EAEC prevalence among diarrhoeal cases from 18 countries published between 2002 to 2023 (see reference 3 for countries and references).<br><br>No additional data meeting the inclusion criteria were identified during the internal FERG review and WHO country consultation period.
**AGE DISTRIBUTION**	For all other countries, we used the country- and year-specific age distribution of diarrhoeal cases and of deaths from GBD 2021 diarrhoea estimates, for EAEC cases and deaths, respectively.
**SEX DISTRIBUTION**	For all other countries, we used the country- and year-specific sex distribution of diarrhoeal cases and of deaths from GBD 2021 diarrhoea estimates, for EAEC cases and deaths, respectively.

**GEOGRAPHIC DISTRIBUTION**	While EAEC is present worldwide, we did not generate estimates for subregion A countries due to lack of data.

Enteropathogenic *E. coli*

**HAZARD**	**Typical enteropathogenic *Escherichia coli* (EPEC)**
**INCIDENCE**	<u>Subregion A countries:</u><br>We initially intended to use estimates from national, population-level studies of the incidence of EPEC diarrhoea identified via the scoping review,[1,3] that identified 6 data points from 5 studies estimating the national, population-level incidence of EPEC diarrhoea, from the following 4 countries (data point by year of study publication, see reference 3 for references): Australia (2000, 2010, 2019), Denmark (2001), Germany (2013) and the United Kingdom of Great Britain and Northern Ireland (1999). However, given feedback to WHO during their country consultation process that the resulting estimates was orders of magnitude too large for current circumstances, we determined there were not enough data to generate reliable estimates of EPEC incidence, mortality, and burden in subregion A countries.<br><br><u>All other countries:</u><br>We used 191 data points from 73 out-patient and community-based studies on EPEC prevalence among diarrhoeal cases from 35 countries published between 1996 to 2023 (see reference 3 for countries and references).<br><br>No additional data meeting the inclusion criteria were identified during the internal FERG review and WHO country consultation period.<br><br>In the meta-analysis,[3] estimates for the proportion of diarrhoea due to atypical EPEC were negative, so we only included typical EPEC estimates in our analyses here.
**CLINICAL OUTCOMES**	Clinical outcomes were acute EPEC diarrhoea (severe), acute EPEC diarrhoea (moderate), acute EPEC diarrhoea (mild), and death following diarrhoea.<br><br>We assumed that 0.5% of EPEC diarrhoeal cases result in severe diarrhoea, 8.5% of EPEC diarrhoeal cases result in moderate diarrhoea, and 91% of EPEC diarrhoeal cases result in mild diarrhoea.[5]
**DURATION**	In children 0-4 years of age, we assumed the duration of severe diarrhoea was 8.4 days, moderate diarrhoea was 6.4 days, and mild diarrhoea was 4.3 days.[5] In persons ≥5 years of age, we assumed the duration of EPEC diarrhoea was 2.8 days.[5]
**DISABILITY WEIGHT**	We used GBD 2019 disability weights, from Salomon et al,[9] as follows.<br><br>For acute EPEC diarrhoea (severe), we used the GBD disability weight for the “diarrhoea, severe” (“has diarrhoea three or more times a day with severe belly cramps. The person is very thirsty and feels nauseous and tired”). Disability weight: 0.247 (UI: 0.164 to 0.348).

	For acute EPEC diarrhoea (moderate), we used the GBD disability weight for “diarrhoea, moderate” (“has diarrhoea three or more times a day, with painful cramps in the belly and feeling thirsty”). Disability weight: 0.188 (UI: 0.125 to 0.264).<br><br>For acute EPEC diarrhoea (mild), we used the GBD disability weight for “diarrhoea, mild” (“has diarrhoea three or more times a day with occasional discomfort in the belly”). Disability weight: 0.074 (UI: 0.049 to 0.104).
**MORTALITY**	Subregion A countries:<br>We did not estimate mortality, as described above for incidence.<br><br>All other countries:<br>We used 113 data points from 33 in-patient studies on EPEC prevalence among diarrhoeal cases from 23 countries published between 2007 to 2023 (see reference 3 for countries and references).<br><br>No additional data meeting the inclusion criteria were identified during the internal FERG review and WHO country consultation period.<br><br>In the meta-analysis,[3] estimates for the proportion of diarrhoea due to atypical EPEC were negative, so we only included typical EPEC estimates in our analyses here.
**AGE DISTRIBUTION**	For all other countries, we used the country- and year-specific age distribution of diarrhoeal cases and of deaths from GBD 2021 diarrhoea estimates, for EPEC cases and deaths, respectively.
**SEX DISTRIBUTION**	For all other countries, we used the country- and year-specific sex distribution of diarrhoeal cases and of deaths from GBD 2021 diarrhoea estimates, for EPEC cases and deaths, respectively.
**GEOGRAPHIC DISTRIBUTION**	While EPEC is present world-wide, we did not generate estimates for subregion A countries due to lack of suitable data.

Enterotoxigenic *E. coli*

**HAZARD**	**Enterotoxigenic *E. coli* (ETEC)**
**INCIDENCE**	Subregion A countries:<br>We used 4 data points from 4 studies estimating the national, population-level incidence of ETEC diarrhoea from a WHO-commissioned update of a scoping review,[1,3] from the following 3 countries (data point by year of study publication, see reference 3 for references): Canada (2013), United Kingdom of Great Britain and Northern Ireland (1999), and United States of America (1999, 2011). No additional data meeting the inclusion criteria were identified during the FERG internal review and WHO country consultation period. Data points included the total estimated number of illnesses (median, mean) or the incidence per population, with some measure of uncertainty (credible interval, confidence interval), when available.<br><br>All other countries:

	We used 423 data points from 84 out-patient and community-based studies on ETEC prevalence among diarrhoeal cases from 38 countries published between 1996 to 2023 (see reference 3 for countries and references).<br><br>No additional data meeting the inclusion criteria were identified during the internal FERG review and WHO country consultation period.<br><br>In the meta-analysis,[3] the incidence due to heat-labile enterotoxin ETEC (LT-ETEC) and heat-stable enterotoxin ETEC (ST-ETEC) were estimated separately then combined into overall ETEC incidence.
**CLINICAL OUTCOMES**	Clinical outcomes were acute ETEC diarrhoea (severe), acute ETEC diarrhoea (moderate), acute ETEC diarrhoea (mild), and death following diarrhoea.<br><br>We assumed that 0.5% of ETEC diarrhoeal cases result in severe diarrhoea, 8.5% of ETEC diarrhoeal cases result in moderate diarrhoea, and 91% of ETEC diarrhoeal cases result in mild diarrhoea.[5]
**DURATION**	In children 0-4 years of age, we assumed the duration of severe diarrhoea was 8.4 days, moderate diarrhoea was 6.4 days, and mild diarrhoea was 4.3 days.[5] In persons ≥5 years of age, we assumed the duration of ETEC diarrhoea was 2.8 days.[5]
**DISABILITY WEIGHT**	We used GBD 2019 disability weights, from Salomon et al,[9] as follows.<br><br>For acute ETEC diarrhoea (severe), we used the GBD disability weight for the “diarrhoea, severe” (“has diarrhoea three or more times a day with severe belly cramps. The person is very thirsty and feels nauseous and tired”). Disability weight: 0.247 (UI: 0.164 to 0.348).<br><br>For acute ETEC diarrhoea (moderate), we used the GBD disability weight for “diarrhoea, moderate” (“has diarrhoea three or more times a day, with painful cramps in the belly and feeling thirsty”). Disability weight: 0.188 (UI: 0.125 to 0.264).<br><br>For acute ETEC diarrhoea (mild), we used the GBD disability weight for “diarrhoea, mild” (“has diarrhoea three or more times a day with occasional discomfort in the belly”). Disability weight: 0.074 (UI: 0.049 to 0.104).
**MORTALITY**	<u>Subregion A countries:</u><br>We estimated zero mortality using 3 estimates from 3 national, population-level studies of the mortality due to ETEC in the following 2 countries: Canada (2015) and United States of America (1999 and 2011) (data point by year of study publication, see reference 3 for references), identified from a WHO-commissioned update of a scoping review.[1,3] No additional data meeting the inclusion criteria were identified during the FERG internal review and WHO country consultation period. Data points included the total estimated number of deaths (median, mean) or the incidence per population, with some measure of uncertainty (credible interval, confidence interval), when available.<br><br><u>All other countries:</u><br>We used 210 data points from 31 in-patient studies on ETEC prevalence among diarrhoeal cases, from X countries published between 20007 to 2023 (see reference 3 for countries and references).

	No additional data meeting the inclusion criteria were identified during the internal FERG review and WHO country consultation period.<br><br>In the meta-analysis,[3] mortality due to LT-ETEC and ST-ETEC were estimated separately and the estimate for LT-ETEC mortality was negative; thus, we only included ST-ETEC mortality estimates in our analyses here.
**AGE DISTRIBUTION**	For subregion A countries, population-level age distributions for ETEC were not available. Thus, we used age distributions for *Campylobacter* cases from the US Centers for Disease Control and Prevention (CDC) FoodNet surveillance reports for the years 2000, 2010, and 2021.[10-12] We assumed the same age distribution for cases and deaths.<br><br>For all other countries, we used the country- and year-specific age distribution of cases and of deaths from GBD 2021 diarrhoea estimates, for ETEC cases and deaths, respectively.
**SEX DISTRIBUTION**	For subregion A countries, we assumed 50% of ETEC cases and deaths were male.[5]<br><br>For all other countries, we used the country- and year-specific sex distribution of diarrhoeal cases and of deaths from GBD 2021 diarrhoea estimates, for ETEC cases and deaths, respectively.
**GEOGRAPHIC DISTRIBUTION**	Present worldwide.

Giardia duodenalis

**HAZARD**	***Giardia duodenalis***
**INCIDENCE**	<u>Subregion A countries:</u><br>We used 9 data points from 9 studies estimating the national, population-level incidence of *Giardia* diarrhoea from a WHO-commissioned update of a scoping review,[1,3] from the following 5 countries (data point by year of study publication, see reference 3 for references): Australia (2005, 2014), Canada (2013), Netherlands (Kingdom of the) (2012), United Kingdom of Great Britain and Northern Ireland (1999, 2012), and United States of America (1999, 2011, 2011). To this, we added 3 additional data points from 3 studies identified during the internal FERG review and WHO country consultation period that met the scoping review's inclusion criteria in the following 2 countries: Netherlands (Kingdom of the) (2017, 2024) and New Zealand (2011) (for a total of 12 data points from 12 studies in 6 countries). Data points included the total estimated number of illnesses (median, mean) or the incidence per population, with some measure of uncertainty (credible interval, confidence interval), when available.<br><br><u>All other countries:</u><br>We used 195 data points from 71 out-patient and community-based studies on *Giardia* prevalence among diarrhoeal cases from 40 countries published between 1993 to 2023 (see reference 3 for countries and references).

	No additional data meeting the inclusion criteria were identified during the internal FERG review and WHO country consultation period.
**CLINICAL OUTCOMES**	Clinical outcomes were acute *Giardia* diarrhoea (severe), acute *Giardia* diarrhoea (moderate), acute *Giardia* diarrhoea (mild), and death following diarrhoea.<br><br>We assumed that 0.5% of *Giardia* diarrhoeal cases result in severe diarrhoea, 8.5% of *Giardia* diarrhoeal cases result in moderate diarrhoea, and 91% of *Giardia* diarrhoeal cases result in mild diarrhoea.[5]
**DURATION**	In children 0-4 years of age, we assumed the duration of severe diarrhoea was 8.4 days, moderate diarrhoea was 6.4 days, and mild diarrhoea was 4.3 days.[5] In persons ≥5 years of age, we assumed the duration of *Giardia* diarrhoea was 2.8 days.[5]
**DISABILITY WEIGHT**	We used GBD 2019 disability weights, from Salomon et al,[9] as follows.<br><br>For acute *Giardia* diarrhoea (severe), we used the GBD disability weight for the "diarrhoea, severe" ("has diarrhoea three or more times a day with severe belly cramps. The person is very thirsty and feels nauseous and tired"). Disability weight: 0.247 (UI: 0.164 to 0.348).<br><br>For acute *Giardia* diarrhoea (moderate), we used the GBD disability weight for "diarrhoea, moderate" ("has diarrhoea three or more times a day, with painful cramps in the belly and feeling thirsty"). Disability weight: 0.188 (UI: 0.125 to 0.264).<br><br>For acute *Giardia* diarrhoea (mild), we used the GBD disability weight for "diarrhoea, mild" ("has diarrhoea three or more times a day with occasional discomfort in the belly"). Disability weight: 0.074 (UI: 0.049 to 0.104).
**MORTALITY**	We assumed zero mortality from *Giardia* in all countries.[5]
**AGE DISTRIBUTION**	For subregion A countries, we used age distributions for *Giardia* cases from national surveillance in the United States of America for the years 2002, 2010 and 2021.[16-18] We assumed the same age distribution for cases and deaths.<br><br>For all other countries, we used the country- and year-specific age distribution of cases and of deaths from GBD 2021 diarrhoea estimates, for *Giardia* cases and deaths, respectively.
**SEX DISTRIBUTION**	For subregion A countries, we assumed 50% of *Giardia* cases and deaths were male.[5]<br><br>For all other countries, we used the country- and year-specific sex distribution of diarrhoeal cases and of deaths from GBD 2021 diarrhoea estimates, for *Giardia* cases and deaths, respectively.
**GEOGRAPHIC DISTRIBUTION**	Present worldwide.

Norovirus

HAZARD	Norovirus
INCIDENCE	Subregion A countries:<br>We used 15 data points from 15 studies estimating the national, population-level incidence of norovirus diarrhoea from a WHO-commissioned update of a scoping review,[1,3] from the following 8 countries (data point by year of study publication, see reference 3 for references): Australia (2005, 2014, 2023), Barbados (2013), Canada (2013), Denmark (2020, 2022), France (2017), Netherlands (Kingdom of the) (2012, 2013), United Kingdom of Great Britain and Northern Ireland (2012), and United States of America (1999, 2011, 2011, 2016). To this, we added 5 additional data points from 4 studies identified during the internal FERG review and WHO country consultation period that met the scoping review's inclusion criteria in the following 3 countries: Netherlands (Kingdom of the) (2017, 2024), New Zealand (2014 [2 data points from 2011 and 2013]), and United States of America (2025) (for a total of 20 data points from 19 studies in 9 countries). Data points included the total estimated number of illnesses (median, mean) or the incidence per population, with some measure of uncertainty (credible interval, confidence interval), when available.<br><br>All other countries:<br>We used 219 data points from 78 out-patient and community-based studies on norovirus prevalence among diarrhoeal cases from 37 countries published between 1993to 2023 (see reference 3 for countries and references).<br><br>No additional data meeting the inclusion criteria were identified during the internal FERG review and WHO country consultation period.<br><br>For all countries, we estimated the additional incidence of vomiting-only norovirus by multiplying the incidence of diarrhoeal norovirus by 19% (minimum 14% - maximum 23%).[5]
CLINICAL OUTCOMES	Clinical outcomes were acute norovirus diarrhoea (severe), acute norovirus diarrhoea (moderate), acute norovirus diarrhoea (mild), norovirus vomiting, and death following diarrhoea.<br><br>We assumed that 0.5% of norovirus diarrhoeal cases result in severe diarrhoea, 8.5% of norovirus diarrhoeal cases result in moderate diarrhoea, and 91% of norovirus diarrhoeal cases result in mild diarrhoea.[5]
DURATION	In children 0-4 years of age, we assumed the duration of severe norovirus diarrhoea was 8.4 days, moderate diarrhoea was 5.05 days, and mild diarrhoea was 4.3 days. In persons ≥5 years of age, we assumed the duration of norovirus diarrhoea was 2.8 days. The same durations were used for norovirus vomiting.
DISABILITY WEIGHT	We used GBD 2019 disability weights, from Salomon et al,[9] as follows.<br><br>For acute norovirus diarrhoea (severe), we used the GBD disability weight for the "diarrhoea, severe" ("has diarrhoea three or more times a day with severe belly cramps. The person is very thirsty and feels nauseous and tired"). Disability weight: 0.247 (UI: 0.164 to 0.348).

	For acute norovirus diarrhoea (moderate), we used the GBD disability weight for "diarrhoea, moderate" ("has diarrhoea three or more times a day, with painful cramps in the belly and feeling thirsty"). Disability weight: 0.188 (UI: 0.125 to 0.264).<br><br>For acute norovirus diarrhoea (mild), we used the GBD disability weight for "diarrhoea, mild" ("has diarrhoea three or more times a day with occasional discomfort in the belly"). Disability weight: 0.074 (UI: 0.049 to 0.104).<br><br>For norovirus vomiting, we used the GBD disability weight for "diarrhoea, mild" ("has diarrhoea three or more times a day with occasional discomfort in the belly"). Disability weight: 0.074 (Uncertainty interval [UI]: 0.049 to 0.104).
**MORTALITY**	<u>Subregion A countries:</u><br>We used 11 data points from 11 national, population-level studies of the mortality due to norovirus diarrhoea from a WHO-commissioned update of a scoping review,[1,3] from the following 7 countries (data point by year of study publication, see reference 3 for references): Australia (2014, 2023), Canada (2015), Denmark (2020, 2022), France (2017), Netherlands (Kingdom of the) (2012, 2013), United Kingdom of Great Britain and Northern Ireland (2020), and United States of America (1999, 2011). To this, we added 6 additional data points from 5 studies identified during the internal FERG review and WHO country consultation period that met the scoping review's inclusion criteria in the following 3 countries: Netherlands (Kingdom of the) (2017, 2024), New Zealand (2011, 2014 [2 data points from 2011 and 2013]), and United States of America (2025) (for a total of 17 data points from 16 studies in 8 countries). Data points included the total estimated number of illnesses (median, mean) or the incidence per population, with some measure of uncertainty (credible interval, confidence interval), when available.<br><br><u>All other countries:</u><br>We used 159 data points from 48 in-patient studies on norovirus prevalence among diarrhoeal cases, from 28 countries published between 2004 to 2023 (see reference 3 for countries and references).<br><br>No additional data meeting the inclusion criteria were identified during the internal FERG review and WHO country consultation period.
**AGE DISTRIBUTION**	For subregion A countries, we used age distributions for norovirus cases from the 2nd Infectious Intestinal Disease (IID2) Study in the United Kingdom of Great Britain and Northern Ireland,[19] applied to all years. We assumed the same age distribution for cases and deaths.<br><br>For all other countries, we used the country- and year-specific age distribution of cases and of deaths from GBD 2021 diarrhoea estimates, for norovirus cases and deaths, respectively.
**SEX DISTRIBUTION**	For subregion A countries, we assumed 50% of norovirus cases and deaths were male.[5]<br><br>For all other countries, we used the country- and year-specific sex distribution of diarrhoeal cases and of deaths from GBD 2021 diarrhoea estimates, for norovirus cases and deaths, respectively.

**GEOGRAPHIC DISTRIBUTION**	Present worldwide.

Rotavirus

**HAZARD**	**Rotavirus**
**INCIDENCE**	Subregion A countries:<br>We used 10 data points from 10 studies estimating the national, population-level incidence of rotavirus diarrhoea from a WHO-commissioned update of a scoping review,[1,3] from the following 5 countries (data point by year of study publication, see reference 3 for references): Australia (2005, 2014), Canada (2013), Netherlands (Kingdom of the) (2012), United Kingdom of Great Britain and Northern Ireland (1999, 2012), and United States of America (1999, 2011, 2011, 2016). To this, we added 2 additional data points from 2 studies identified during the internal FERG review and WHO country consultation period that met the scoping review's inclusion criteria in the following country: Netherlands (Kingdom of the) (2017, 2024) (for a total of 12 data points from 12 studies in 5 countries). Data points included the total estimated number of illnesses (median, mean) or the incidence per population, with some measure of uncertainty (credible interval, confidence interval), when available.<br><br>All other countries:<br>We used 257 data points from 104 out-patient and community-based studies on rotavirus prevalence among diarrhoeal cases from 45 countries published between 1993 to 2023 (see reference 3 for countries and references).<br><br>No additional data meeting the inclusion criteria were identified during the internal FERG review and WHO country consultation period.
**CLINICAL OUTCOMES**	Clinical outcomes were acute rotavirus diarrhoea (severe), acute rotavirus diarrhoea (moderate), acute rotavirus diarrhoea (mild), and death following diarrhoea.<br><br>We assumed that 0.5% of rotavirus diarrhoeal cases result in severe diarrhoea, 8.5% of rotavirus diarrhoeal cases result in moderate diarrhoea, and 91% of rotavirus diarrhoeal cases result in mild diarrhoea.[5]
**DURATION**	In children 0-4 years of age, we assumed the duration of severe rotavirus diarrhoea was 8.4 days, moderate diarrhoea was 5.05 days, and mild diarrhoea was 4.3 days. In persons ≥5 years of age, we assumed the duration of rotavirus diarrhoea was 2.8 days.
**DISABILITY WEIGHT**	We used GBD 2019 disability weights, from Salomon et al,[9] as follows.<br><br>For acute rotavirus diarrhoea (severe), we used the GBD disability weight for the "diarrhoea, severe" ("has diarrhoea three or more times a day with severe belly cramps. The person is very thirsty and feels nauseous and tired"). Disability weight: 0.247 (UI: 0.164 to 0.348).<br><br>For acute rotavirus diarrhoea (moderate), we used the GBD disability weight for "diarrhoea, moderate" ("has diarrhoea three or more times a day, with painful cramps in the belly and feeling thirsty"). Disability weight: 0.188 (UI: 0.125 to 0.264).

	For acute rotavirus diarrhoea (mild), we used the GBD disability weight for "diarrhoea, mild" ("has diarrhoea three or more times a day with occasional discomfort in the belly"). Disability weight: 0.074 (UI: 0.049 to 0.104).
**MORTALITY**	<u>Subregion A countries:</u><br>We used 5 estimates from 5 national, population-level studies of the mortality due to rotavirus diarrhoea from a WHO-commissioned update of a scoping review,[1,3] from the following 4 countries (data point by year of study publication, see reference 3 for references): Australia (2014), Netherlands (Kingdom of the) (2012), United Kingdom of Great Britain and Northern Ireland (2020), and United States of America (1999, 2011). To this, we added 3 additional data points from 3 studies identified during the internal FERG review and WHO country consultation period that met the scoping review's inclusion criteria in the following 2 countries: Netherlands (Kingdom of the) (2017, 2024) and New Zealand (2011) (for a total of 8 data points from 8 studies in 5 countries). Data points included the total estimated number of illnesses (median, mean) or the incidence per population, with some measure of uncertainty (credible interval, confidence interval), when available.<br><br><u>All other countries:</u><br>We used 195 data points from 67 in-patient studies on rotavirus prevalence among diarrhoeal cases, from 35 countries published between 1999 to 2023 (see reference 3 for countries and references).<br><br>No additional data meeting the inclusion criteria were identified during the internal FERG review and WHO country consultation period.
**AGE DISTRIBUTION**	For subregion A countries, we used age distributions for rotavirus cases Australia's national surveillance system for the years 2010 and 2021.[20] We assumed the same age distribution for cases and deaths.<br><br>For all other countries, we used the country- and year-specific age distribution of cases and of deaths from GBD 2021 diarrhoea estimates, for rotavirus cases and deaths, respectively.
**SEX DISTRIBUTION**	For subregion A countries, we assumed 50% of rotavirus cases and deaths were male.[5]<br><br>For all other countries, we used the country- and year-specific sex distribution of diarrhoeal cases and of deaths from GBD 2021 diarrhoea estimates, for rotavirus cases and deaths, respectively.
**GEOGRAPHIC DISTRIBUTION**	Present worldwide.

Salmonella spp. (non-typhoidal)

**HAZARD**	**Non-typhoidal *Salmonella enterica* (NTS)**
**INCIDENCE**	<u>Subregion A countries:</u>

	We used 30 data points from 24 studies estimating the national, population-level incidence of NTS diarrhoea from a WHO-commissioned update of a scoping review,[1,3] from the following 14 countries (data point by year of study publication, see reference 3 for references): Australia (2005, 2014, 2019, 2023), Barbados (2013), Canada (2006, 2013, 2022), Denmark (2013, 2020, 2022), France (2015, 2017), Germany (2013), Italy (2013), Japan (2008, 2011), Netherlands (Kingdom of the) (2012, 2013), New Zealand (2000), Poland (2013), Sweden (2013, 2018), United Kingdom of Great Britain and Northern Ireland (1999, 2012, 2013), and United States of America (1999, 2004, 2011, 2011). To this, we added 6 additional data points from 5 studies identified during the internal FERG review and WHO country consultation period that met the scoping review's inclusion criteria in the following 3 countries: Netherlands (Kingdom of the) (2017, 2024), New Zealand (2011, 2014 [2 data points from 2011 and 2013]) and United States of America (2025) (for a total of 36 data points from 29 studies in 14 countries). Data points included the total estimated number of illnesses (median, mean) or the incidence per population, with some measure of uncertainty (credible interval, confidence interval), when available.<br><br><u>All other countries:</u><br>We used 238 data points from 97 out-patient and community-based studies on NTS prevalence among diarrhoeal cases, from 41 countries published between 1993 to 2023 (see reference 3 for countries and references).<br><br>No additional data meeting the inclusion criteria were identified during the internal FERG review and WHO country consultation period.
**CLINICAL OUTCOMES**	Clinical outcomes were acute NTS diarrhoea (severe), acute NTS diarrhoea (moderate), acute NTS diarrhoea (mild), and death following diarrhoea.<br><br>The additional outcome of invasive NTS infection is described below, and was estimated separately from diarrhoeal NTS.<br><br>We assumed that 2% of NTS diarrhoeal cases result in severe diarrhoea, 25% of NTS diarrhoeal cases result in moderate diarrhoea, and 73% of NTS diarrhoeal cases result in mild diarrhoea.[5]
**DURATION**	In children 0-4 years of age, we assumed the duration of severe diarrhoea was 8.4 days, moderate diarrhoea was 6.4 days, and mild diarrhoea was 4.3 days.[5] In persons ≥5 years of age, we assumed the duration of NTS diarrhoea was 2.8 days.[5]
**DISABILITY WEIGHT**	We used GBD 2019 disability weights, from Salomon et al,[9] as follows.<br><br>For acute NTS diarrhoea (severe), we used the GBD disability weight for the "diarrhoea, severe" ("has diarrhoea three or more times a day with severe belly cramps. The person is very thirsty and feels nauseous and tired"). Disability weight: 0.247 (UI: 0.164 to 0.348).<br><br>For acute NTS diarrhoea (moderate), we used the GBD disability weight for "diarrhoea, moderate" ("has diarrhoea three or more times a day, with painful cramps in the belly and feeling thirsty"). Disability weight: 0.188 (UI: 0.125 to 0.264).

	For acute NTS diarrhoea (mild), we used the GBD disability weight for "diarrhoea, mild" ("has diarrhoea three or more times a day with occasional discomfort in the belly"). Disability weight: 0.074 (UI: 0.049 to 0.104).
**MORTALITY**	<u>Subregion A countries:</u><br>We used 15 data points from 15 studies estimating the national, population-level mortality due to NTS diarrhoea from a WHO-commissioned update of a scoping review,[1,3] from the following 9 countries (data point by year of study publication, see reference 3 for references): Australia (2014, 2019, 2023), Canada (2015, 2022), Denmark (2020, 2022), France (2017), Netherlands (Kingdom of the) (2012), New Zealand (2000), United Kingdom of Great Britain and Northern Ireland (2020), Sweden (2018), United States of America (1999, 2004, 2011). To this, we added 6 additional data points from 5 studies identified during the internal FERG review and WHO country consultation period that met the scoping review's inclusion criteria in the following 3 countries: Netherlands (Kingdom of the) (2017, 2024), New Zealand (2011, 2014 [two data points from 2011 and 2013]) and United States of America (2025) (for a total of 21 data points from 20 studies in 9 countries). Data points included the total estimated number of deaths (median, mean) or the incidence per population, with some measure of uncertainty (credible interval, confidence interval), when available.<br><br><u>All other countries:</u><br>We used 138 data points from 39 in-patient studies on NTS prevalence among diarrhoeal cases, from 30 countries published between 2002 to 2023 (see reference 3 for countries and references).<br><br>No additional data meeting the inclusion criteria were identified during the internal FERG review and WHO country consultation period.
**AGE DISTRIBUTION**	For subregion A countries, we used age distributions for NTS cases from the US Centers for Disease Control and Prevention (CDC) FoodNet surveillance reports for the years 2000, 2010, and 2021.[10-12] We assumed the same age distribution for cases and deaths.<br><br>For all other countries, we used the country- and year-specific age distribution of cases and of deaths from GBD 2021 diarrhoea estimates, for NTS cases and deaths, respectively.
**SEX DISTRIBUTION**	For subregion A countries, we assumed 50% of diarrhoeal NTS cases and deaths were male.[5]<br><br>For all other countries, we used the country- and year-specific sex distribution of diarrhoeal cases and of deaths from GBD 2021 diarrhoea estimates, for diarrhoeal NTS cases and deaths, respectively.
**GEOGRAPHIC DISTRIBUTION**	Present worldwide.

Shiga toxin-producing *E. coli*

HAZARD	**Shiga toxin-producing *Escherichia coli* (STEC)**
**INCIDENCE**	Subregion A countries:<br>We used 19 data points from 16 studies estimating the national, population-level incidence of STEC diarrhoea from a WHO-commissioned update of a scoping review,[1,3] from the following 10 countries (data point by year of study publication, see reference 3 for references): Australia (2005, 2014, 2023), Canada (2006, 2013), Denmark (2013, 2020, 2022), France (2017), Germany (2013, 2016), Netherlands (Kingdom of the) (2013), New Zealand (2000), United Kingdom of Great Britain and Northern Ireland (1999, 2013), Sweden (2013, 2018), and United States of America (1999, 2011). To this, we added 3 additional data points from 2 studies identified during the internal FERG review and WHO country consultation period that met the scoping review's inclusion criteria in the following 2 countries: New Zealand (2014 [2 data points from 2011 and 2013]) and United States of America (2025) (for a total of 22 data points from 18 studies in 10 countries). Data points included the total estimated number of illnesses (median, mean) or the incidence per population, with some measure of uncertainty (credible interval, confidence interval), when available.<br><br>All other countries:<br>We used 161 data points from 48 out-patient and community-based studies on STEC prevalence among diarrhoeal cases from 32 countries published between 1999 to 2023 (see reference 3 for countries and references).<br><br>No additional data meeting the inclusion criteria were identified during the internal FERG review and WHO country consultation period.
**CLINICAL OUTCOMES**	Clinical outcomes were acute STEC diarrhoea (severe), acute STEC diarrhoea (moderate), acute STEC diarrhoea (mild), haemolytic-uremic syndrome (HUS) following STEC infection (STEC-HUS), end-stage renal disease (ESRD) following STEC-HUS, and death following all of them.<br><br>We assumed that 2% of STEC cases result in severe diarrhoea, 18% of STEC cases result in moderate diarrhoea, and 80% of STEC cases result in mild diarrhoea.[5]<br><br>To estimate the proportion of STEC cases that develop HUS, we used results from a WHO-commissioned systematic review (PROSPERO: CRD42023485066) and meta-analysis that estimated the proportion of O157 STEC and non-O157 STEC cases that develop HUS, by age group (0-19 years, ≥20 years), adjusted for under-reporting. We used 729 data points from 273 studies to estimate the proportion of STEC cases that develop HUS (see data table in reference 4). To this, we added 1 additional data point from 1 study identified during the internal FERG review and WHO country consultation period that met the review's inclusion criteria (Norway). We used 29 estimates from 25 studies to estimate the proportion of STEC-HUS cases that develop ESRD (see data table in reference 4). No additional data meeting the inclusion criteria were identified during the internal FERG review and WHO country consultation period.<br><br>We stratified our STEC incidence estimates into O157 and non-O157 fractions, as follows, and applied the relevant proportion developing HUS. In AMR A, AMR B, EUR, and WPR A, we used published, national population-level incidence estimates identified via the updated scoping review,[1,3] to estimate 31.1% of STEC cases were

	O157 in these subregions. In AMR D, the African (AFR) and South-East Asia (SEAR) Regions, and EMR, we assumed 10% of STEC cases were O157.[5,21]<br><br>To estimate the proportion of STEC-HUS cases that develop ESRD, we used results from the above systematic review to estimate this proportion.
**DURATION**	In persons of all ages, we assumed the duration of STEC diarrhoea was 7 days (minimum 5 days - maximum 10 days), the duration of STEC-HUS was 28 days (minimum 14 days - maximum 42 days), and the duration of ESRD following STEC-HUS was lifelong.[5]
**DISABILITY WEIGHT**	We used GBD 2019 disability weights, from Salomon et al,[9] as follows.<br><br>For acute STEC diarrhoea (severe), we used the GBD disability weight for the "diarrhoea, severe" ("has diarrhoea three or more times a day with severe belly cramps. The person is very thirsty and feels nauseous and tired"). Disability weight: 0.247 (UI: 0.164 to 0.348).<br><br>For acute STEC diarrhoea (moderate), we used the GBD disability weight for "diarrhoea, moderate" ("has diarrhoea three or more times a day, with painful cramps in the belly and feeling thirsty"). Disability weight: 0.188 (UI: 0.125 to 0.264).<br><br>For acute STEC diarrhoea (mild), we used the GBD disability weight for "diarrhoea, mild" ("has diarrhoea three or more times a day with occasional discomfort in the belly"). Disability weight: 0.074 (UI: 0.049 to 0.104).<br><br>For STEC-HUS, we used as a proxy the GBD disability weight for the "diarrhoea, severe" ("has diarrhoea three or more times a day with severe belly cramps. The person is very thirsty and feels nauseous and tired"). Disability weight: 0.247 (UI:164 to 0.348).<br><br>For ESRD following STEC-HUS, we used the GBD disability weight "end-stage renal disease, on dialysis" ("is tired and has itching, cramps, headache, joint pains and shortness of breath. The person needs intensive medical care every other day lasting about half a day"). Disability weight: 0.571(UI: 0.398 to 0.725).
**MORTALITY**	Subregion A countries:<br>We used 10 data points from 10 studies estimating the national, population-level mortality due to STEC diarrhoea from a WHO-commissioned update of a scoping review,[1,3] from the following 7 countries (data point by year of study publication, see reference 3 for references): Australia (2014, 2023), Canada (2016), Denmark (2020, 2022), France (2017), New Zealand (2000), Sweden (2018), and United States of America (1999, 2011). To this, we added 4 additional data points from 3 studies identified during the internal FERG review and WHO country consultation period that met the scoping review's inclusion criteria in the following 2 countries New Zealand (2011, 2014 [two data points from 2011 and 2013]) and United States of America (2025) (for a total of 14 data points form 13 studies in 7 countries). Data points included the total estimated number of deaths (median, mean) or the incidence per population, with some measure of uncertainty (credible interval, confidence interval), when available.<br><br>All other countries:

	We used 76 data points from 16 in-patient studies on STEC prevalence among diarrhoeal cases, from 17 countries published between 2010 to 2022 (see reference 3 for countries and references).<br><br>No additional data meeting the inclusion criteria were identified during the internal FERG review and WHO country consultation period.<br><br><u>HUS and ESRD:</u><br>For mortality following STEC-HUS, in subregion A countries we assumed the case-fatality ratio in children 0-17 years was 2% (minimum 0% - maximum 3%), and for persons ≥18 years, we assumed a case fatality ratio of 4%.[21-28] In all other countries, we assumed a case fatality ratio of 4% for persons of all ages.<br><br>For mortality following ESRD, we used as our case fatality ratio the country-specific difference between the proportion of patients needing renal replacement therapy (RRT), and the proportion receiving RRT, calculated from a prior systematic review of RRT use worldwide.[29] We used the conservative estimates from this review, and for countries where differences were implausible or data were missing, we assigned the average value from the WHO subregion (data in appendix 2).
**AGE DISTRIBUTION**	For diarrhoeal cases, for subregion A countries, we used age distributions for STEC cases from the US Centers for Disease Control and Prevention (CDC) FoodNet surveillance reports for the years 2000, 2010, and 2021.[10-12] We assumed the same age distribution for cases and deaths.<br><br>For all other countries, we used the country- and year-specific age distribution of cases and of deaths from GBD 2021 diarrhoea estimates, for STEC cases and deaths, respectively.<br><br>For STEC-HUS cases, we assumed 40% were 0-4 years, 19% of cases were 5-14 years, 26% of cases were 15-54 years, and 10% of cases were ≥55 years old.[5]<br><br>For ESRD following STEC, we assumed cases followed the same age distribution as STEC-HUS cases, due to lack of age-specific data.
**SEX DISTRIBUTION**	For subregion A countries, we assumed 50% of STEC cases, STEC-HUS cases, ESRD following STEC, and deaths were male.[5]<br><br>For all other countries, we used the country- and year-specific sex distribution of diarrhoeal cases and of deaths from GBD 2021 diarrhoea estimates, for STEC/HUS/ESRD cases and deaths, respectively.
**GEOGRAPHIC DISTRIBUTION**	Present worldwide.

Shigella spp.

**HAZARD**	***Shigella* spp.**
**INCIDENCE**	<u>Subregion A countries:</u>

	We used 17 data points from 12 studies estimating the national, population-level incidence of *Shigella* diarrhoea from a WHO-commissioned update of a scoping review,[1,3] from the following 11 countries (data point by year of study publication, see reference 3 for references): Australia (2005, 2014, 2023), Canada (2013), Denmark (2013), France (2017), Germany (2013), Netherlands (Kingdom of the) (2013), New Zealand (2000), Poland (2013), Sweden (2013, 2018), United Kingdom of Great Britain and Northern Ireland (1999, 2013), and United States of America (1999, 2011, 2011). To this, we added 1 additional data from one study identified during the internal FERG review and WHO country consultation period that met the scoping review's inclusion criteria in the following country: Netherlands (Kingdom of the) (2016) (for a total of 18 data points from 13 studies in 11 countries). Data points included the total estimated number of illnesses (median, mean) or the incidence per population, with some measure of uncertainty (credible interval, confidence interval), when available.<br><br><u>All other countries:</u><br>We used 242 data points from 104 out-patient and community-based studies on *Shigella* prevalence among diarrhoeal cases, from 42 countries published between 1993 to 2023 (see reference 3 for countries and references).<br><br>No additional data meeting the inclusion criteria were identified during the internal FERG review and WHO country consultation period.
**CLINICAL OUTCOMES**	Clinical outcomes were acute *Shigella* diarrhoea (severe), acute *Shigella* diarrhoea (moderate), acute *Shigella* diarrhoea (mild), and death following diarrhoea.<br><br>We assumed that 2% of *Shigella* cases result in severe diarrhoea, 25% of *Shigella* cases result in moderate diarrhoea, and 73% of *Shigella* cases result in mild diarrhoea.[5]
**DURATION**	In children 0-4 years of age, we assumed the duration of severe diarrhoea was 8.4 days, moderate diarrhoea was 6.4 days, and mild diarrhoea was 4.3 days.[5] In persons ≥5 years of age, we assumed the duration of *Shigella* diarrhoea was 2.8 days.[5]
**DISABILITY WEIGHT**	We used GBD 2019 disability weights, from Salomon et al,[9] as follows.<br><br>For acute *Shigella* diarrhoea (severe), we used the GBD disability weight for the "diarrhoea, severe" ("has diarrhoea three or more times a day with severe belly cramps. The person is very thirsty and feels nauseous and tired"). Disability weight: 0.247 (UI: 0.164 to 0.348).<br><br>For acute *Shigella* diarrhoea (moderate), we used the GBD disability weight for "diarrhoea, moderate" ("has diarrhoea three or more times a day, with painful cramps in the belly and feeling thirsty"). Disability weight: 0.188 (UI: 0.125 to 0.264).<br><br>For acute *Shigella* diarrhoea (mild), we used the GBD disability weight for "diarrhoea, mild" ("has diarrhoea three or more times a day with occasional discomfort in the belly"). Disability weight: 0.074 (UI: 0.049 to 0.104).
**MORTALITY**	<u>Subregion A countries:</u><br>We used 9 data points from 9 studies estimating the national, population-level mortality due to Campylobacter diarrhoea from a WHO-commissioned update of a scoping review,[1,3] from the following 7 countries (data point by year of study

	publication, see reference 3 for references): Australia (2014, 2023), Canada (2016), France (2017), New Zealand (2000), Sweden (2018), United Kingdom of Great Britain and Northern Ireland (2020), and United States of America (1999, 2011). To this, we added 2 additional data points from 2 studies identified during the internal FERG review and WHO country consultation period that met the scoping review's inclusion criteria in the following 2 countries: New Zealand (2011) and Netherlands (Kingdom of the) (2016) (for a total of 11 data points from 11 studies in 8 countries). Data points included the total estimated number of deaths (median, mean) or the incidence per population, with some measure of uncertainty (credible interval, confidence interval), when available.<br><br><u>All other countries:</u><br>We used 145 data points from 44 in-patient studies on *Shigella* prevalence among diarrhoeal cases, from 32 countries published between 2002 to 2023 (see reference 3 for countries and references).<br><br>No additional data meeting the inclusion criteria were identified during the internal FERG review and WHO country consultation period.
**AGE DISTRIBUTION**	For subregion A countries, we used age distributions for *Shigella* cases from the US Centers for Disease Control and Prevention (CDC) FoodNet surveillance reports for the years 2000, 2010, and 2021.[10-12] We assumed the same age distribution for cases and deaths.<br><br>For all other countries, we used the country- and year-specific age distribution of cases and of deaths from GBD 2021 diarrhoea estimates, for *Shigella* cases and deaths, respectively.
**SEX DISTRIBUTION**	For subregion A countries, we assumed 50% of *Shigella* cases and deaths were male.[5]<br><br>For all other countries, we used the country- and year-specific sex distribution of diarrhoeal cases and of deaths from GBD 2021 diarrhoea estimates, for *Shigella* cases and deaths, respectively.
**GEOGRAPHIC DISTRIBUTION**	Present worldwide.

Vibrio cholerae

**HAZARD**	***Vibrio cholerae***
**INCIDENCE**	For countries with cholera transmission (see below), we used 160 data points from 49 out-patient and community-based studies on *Vibrio cholerae* prevalence among diarrhoeal cases, from 23 countries published between 1996 to 2023 (see reference 3 for countries and references).<br><br>No additional data meeting the inclusion criteria were identified during the internal FERG review and WHO country consultation period.

**CLINICAL OUTCOMES**	Clinical outcomes were acute cholera diarrhoea (severe), acute cholera diarrhoea (moderate), acute cholera diarrhoea (mild), and death following diarrhoea.<br><br>We assumed that 35% of cholera cases result in severe diarrhoea, 40% of cholera cases result in moderate diarrhoea, and 25% of cholera cases result in mild diarrhoea.[5]
**DURATION**	In all persons, we assumed the duration of cholera was 7 days (minimum 3 days - maximum duration 10 days).[5]
**DISABILITY WEIGHT**	We used GBD 2019 disability weights, from Salomon et al,[9] as follows.<br><br>For acute cholera diarrhoea (severe), we used the GBD disability weight for the "diarrhoea, severe" ("has diarrhoea three or more times a day with severe belly cramps. The person is very thirsty and feels nauseous and tired"). Disability weight: 0.247 (UI: 0.164 to 0.348).<br><br>For acute cholera diarrhoea (moderate), we used the GBD disability weight for "diarrhoea, moderate" ("has diarrhoea three or more times a day, with painful cramps in the belly and feeling thirsty"). Disability weight: 0.188 (UI: 0.125 to 0.264).<br><br>For acute cholera diarrhoea (mild), we used the GBD disability weight for "diarrhoea, mild" ("has diarrhoea three or more times a day with occasional discomfort in the belly"). Disability weight: 0.074 (UI: 0.049 to 0.104).
**MORTALITY**	For countries with cholera transmission (see below), we used 85 data points from 17 in-patient studies on *Vibrio cholerae* prevalence among diarrhoeal cases, from 13 countries published between 2002 to 2022(see reference 3 for countries and references).<br><br>No additional data meeting the inclusion criteria were identified during the internal FERG review and WHO country consultation period.
**AGE DISTRIBUTION**	For countries with cholera transmission, we used the country- and year-specific age distribution of cases and of deaths from GBD 2021 diarrhoea estimates, for cholera cases and deaths, respectively.
**SEX DISTRIBUTION**	For countries with cholera transmission, we used the country- and year-specific sex distribution of diarrhoeal cases and of deaths from GBD 2021 diarrhoea estimates, for cholera cases and deaths, respectively.
**GEOGRAPHIC DISTRIBUTION**	We assumed countries with no reports of cholera transmission in the prior 10 years were cholera-free.[3] This list included all countries in the WHO AMR region except Haiti, all countries in the EUR region, and all countries in the WPR region except the Philippines.<br><br>The 59 countries considered to have cholera transmission were:<br>AMR: Haiti<br>EMR: Afghanistan, Djibouti, Iraq, Pakistan, Somalia, South Sudan, Sudan, Syrian Arab Republic, Yemen<br>AFR: Angola, Benin, Botswana, Burkina Faso, Burundi, Cabo Verde, Cameroon, Central African Republic, Chad, Comoros, Congo, Cote d'Ivoire, Democratic Republic

	of the Congo, Equatorial Guinea, Eritrea, Eswatini, Ethiopia, Gabon, Gambia, Ghana, Guinea, Guinea-Bissau, Kenya, Lesotho, Liberia, Madagascar, Malawi, Mali, Mauritania, Mauritius, Mozambique, Namibia, Niger, Nigeria, Rwanda, Sao Tome and Principe, Senegal, Seychelles, Sierra Leone, Togo, Uganda, United Republic of Tanzania, Zambia, Zimbabwe<br>SEAR: Bangladesh, India, Myanmar, Nepal<br>WPR: Philippines

REFERENCES

1. Scallan Walter EJ, Griffin PM, Bruce BB, Hoekstra RM. Estimating the Number of Illnesses Caused by Agents Transmitted Commonly Through Food: A Scoping Review. Foodborne Pathog Dis. 2021 Dec;18(12):841-858. doi: 10.1089/fpd.2021.0038.
2. Colston JM, Flynn TG, Denton AH, et al. Updating global estimates of pathogen-attributable diarrhoeal disease burden: a methodology and integrated protocol for a broad-scope systematic review of a syndrome with diverse infectious aetiologies. BMJ Open. 2025 Apr 3;15(4):e093018. doi: 10.1136/bmjopen-2024-093018.
3. Colston J, Devleesschauwer BD, Flynn T, et al. Updated estimates of the global, regional and national burden, and etiology of diarrheal diseases transmissible via food: A systematic review and meta-analytical modelling study for the World Health Organization. medRxiv 2026.01.26.26344508; doi:https://doi.org/10.64898/2026.01.26.26344508
4. Devleesschauwer D, Vaes L, Fernandez K, et al. Computational framework for the World Health Organization estimates of the global, regional and national burden of foodborne diseases 2026 edition. medRxiv 2026.05.13.26353030; doi: https://doi.org/10.64898/2026.05.13.26353030
5. Kirk MD, Pires SM, Black RE, et al. World Health Organization Estimates of the Global and Regional Disease Burden of 22 Foodborne Bacterial, Protozoal, and Viral Diseases, 2010: A Data Synthesis. PLoS Med. 2015 Dec 3;12(12):e1001921. doi: 10.1371/journal.pmed.1001921. Erratum in: PLoS Med. 2015 Dec 23;12(12):e1001940. doi: 10.1371/journal.pmed.1001940.
6. Poropatich KO, Walker CL, Black RE. Quantifying the association between Campylobacter infection and Guillain-Barré syndrome: a systematic review. J Health Popul Nutr. 2010 Dec;28(6):545-52. doi: 10.3329/jhpn.v28i6.6602.
7. Leonhard SE, van der Eijk AA, Andersen H, et al; IGOS Consortium. An International Perspective on Preceding Infections in Guillain-Barré Syndrome: The IGOS-1000 Cohort. Neurology. 2022 Sep 20;99(12):e1299-e1313. doi: 10.1212/WNL.0000000000200885. Epub 2022 Aug 18. PMID: 35981895.
8. Dutta D, Debnath M, Nagappa M, et al. Antecedent infections in Guillain-Barré syndrome patients from south India. J Peripher Nerv Syst. 2021 Sep;26(3):298-306. doi: 10.1111/jns.12459.
9. Salomon JA, Haagsma JA, Davis A, de Noordhout CM, Polinder S, Havelaar AH, Cassini A, Devleesschauwer B, Kretzschmar M, Speybroeck N, Murray CJ, Vos T. Disability weights for the

Global Burden of Disease 2013 study. Lancet Glob Health. 2015 Nov;3(11):e712-23. doi: 10.1016/S2214-109X(15)00069-8.

10. Centers for Disease Control and Prevention. Foodborne Diseases Active Surveillance Network (FoodNet). FoodNet FAST, Pathogen by demographic, 2000 [internet].. U.S. Department of Health & Human Services; 2025. [cited 24 Apr 2025]. Available from: https://wwwn.cdc.gov/foodnetfast/
11. Centers for Disease Control and Prevention. Foodborne Diseases Active Surveillance Network (FoodNet). FoodNet FAST, Pathogen by demographic, 2010 [internet].. U.S. Department of Health & Human Services; 2025. [cited 24 Apr 2025]. Available from: https://wwwn.cdc.gov/foodnetfast/
12. Centers for Disease Control and Prevention. Foodborne Diseases Active Surveillance Network (FoodNet). FoodNet FAST, Pathogen by demographic, 2021 [internet].. U.S. Department of Health & Human Services; 2025. [cited 24 Apr 2025]. Available from: https://wwwn.cdc.gov/foodnetfast/
13. Lin FH, Chen BC, Chou YC, et al. The Epidemiology of *Entamoeba histolytica* Infection and Its Associated Risk Factors among Domestic and Imported Patients in Taiwan during the 2011-2020 Period. Medicina (Kaunas). 2022 Jun 17;58(6):820. doi: 10.3390/medicina58060820.
14. Tam CC, Rodrigues LC, Viviani L, et al; IID2 Study Executive Committee. Longitudinal study of infectious intestinal disease in the UK (IID2 study): incidence in the community and presenting to general practice. Gut. 2012 Jan;61(1):69-77. doi: 10.1136/gut.2011.238386.
15. Wheeler JG, Sethi D, Cowden JM, et al. Study of infectious intestinal disease in England: rates in the community, presenting to general practice, and reported to national surveillance. The Infectious Intestinal Disease Study Executive. BMJ. 1999 Apr 17;318(7190):1046-50. doi: 10.1136/bmj.318.7190.1046.
16. Groseclose SL, Brathwaite WS, Hall PA, et al; Centers for Disease Control and Prevention (CDC). Summary of notifiable diseases--United States, 2002. MMWR Morb Mortal Wkly Rep. 2004 Apr 30;51(53):1-84.
17. Centers for Disease Control and Prevention (CDC). Summary of notifiable diseases--United States, 2010. MMWR Morb Mortal Wkly Rep. 2012 Jun 1;59(53):1-111. Erratum in: MMWR Morb Mortal Wkly Rep. 2012 Jul 27;61:562.
18. Centers for Disease Control and Prevention. National Notifiable Diseases Surveillance System (NNDSS), NNDSS Annual Summary Data 2016-2022 Results [internet]. U.S. Department of Health & Human Services; 2025. [cited 29 Apr 2025]. Available from: https://wonder.cdc.gov/nndss-annual-summary.html
19. Harris JP, Iturriza-Gomara M, O'Brien SJ. Re-assessing the total burden of norovirus circulating in the United Kingdom population. Vaccine. 2017 Feb 7;35(6):853-855. doi: 10.1016/j.vaccine.2017.01.009.
20. Australian Centre for Disease Control. National Notifiable Disease Surveillance System. National Communicable Disease Surveillance Dashboard [internet]. Commonwealth of Australia; 2026 [cited 2026 Jan 17]. Available from: https://nindss.health.gov.au/pbi-dashboard/

21. Majowicz SE, Scallan E, Jones-Bitton A, et al. Global incidence of human Shiga toxin-producing *Escherichia coli* infections and deaths: a systematic review and knowledge synthesis. Foodborne Pathog Dis. 2014 Jun;11(6):447-55. doi: 10.1089/fpd.2013.1704.

22. Gould LH, Demma L, Jones TF, et al. Hemolytic uremic syndrome and death in persons with *Escherichia coli* O157:H7 infection, foodborne diseases active surveillance network sites, 2000-2006. Clin Infect Dis. 2009 Nov 15;49(10):1480-5. doi: 10.1086/644621.

23. Ylinen E, Salmenlinna S, Halkilahti J, et al. Hemolytic uremic syndrome caused by Shiga toxin-producing *Escherichia coli* in children: incidence, risk factors, and clinical outcome. Pediatr Nephrol. 2020 Sep;35(9):1749-1759. doi: 10.1007/s00467-020-04560-0.

24. Frank C, Werber D, Cramer JP, et al; HUS Investigation Team. Epidemic profile of Shiga-toxin-producing Escherichia coli O104:H4 outbreak in Germany. N Engl J Med. 2011 Nov 10;365(19):1771-80. doi: 10.1056/NEJMoa1106483.

25. Monet-Didailler C, Godron-Dubrasquet A, Madden I, Delmas Y, Llanas B, Harambat J. Long-term outcome of diarrhoea-associated haemolytic uremic syndrome is poorly related to markers of kidney injury at 1-year follow-up in a population-based cohort. Pediatr Nephrol. 2019 Apr;34(4):657-662. doi: 10.1007/s00467-018-4131-z.

26. Zagożdżon I, Szczepańska M, Rubik J, et al. Haemolytic uremic syndrome as a cause of chronic kidney disease stage 5 in children is in retreat: results from the Polish Registry of Kidney Replacement Therapy in children (2000-2023). Pediatr Nephrol. 2025 Apr;40(4):1069-1079. doi: 10.1007/s00467-024-06584-2.

27. Zagożdżon I, Szczepańska M, Leszczyńska B, et al. Changing Epidemiology and Outcomes of Hemolytic Uremic Syndrome in Children: A Prospective National Cohort Study from the Polish Pediatric HUS Registry and the Polish Registry of Renal Replacement Therapy in Children. J Clin Med. 2024 Oct 30;13(21):6499. doi: 10.3390/jcm13216499.

28. Myojin S, Michihata N, Shoji K, et al. Prognostic factors among patients with Shiga toxin-producing Escherichia coli haemolytic uremic syndrome: A retrospective cohort study using a nationwide inpatient database in Japan. J Infect Chemother. 2023 Jun;29(6):610-614. doi: 10.1016/j.jiac.2023.02.016.

29. Liyanage T, Ninomiya T, Jha V, et al. Worldwide access to treatment for end-stage kidney disease: a systematic review. Lancet. 2015 May 16;385(9981):1975-82. doi: 10.1016/S0140-6736(14)61601-9.

SUPPLEMENTARY TABLES AND FIGURES

Table S1. Mean rates of foodborne illnesses, deaths, and Disability-Adjusted Life Years (DALYs) per 100,000 persons globally, by age group, with 95% uncertainty intervals (UIs), 2021

PATHOGEN	Age group: <5 years of age			Age group: ≥5 years of age		
	ILLNESSES, Rate (95% UI)	DEATHS, Rate (95% UI)	DALYs, Rate (95% UI)	ILLNESSES, Rate (95% UI)	DEATHS, Rate (95% UI)	DALYs, Rate (95% UI)
*Campylobacter jejuni/coli**	2,748·1 (1,724·6; 4,040·4)	2·27 (1·18; 3·73)	212·4 (110·9; 346·9)	1,801·5 (979·8; 2,992·2)	0·279 (0·130; 0·501)	9·84 (4·85; 16·6)
Cryptosporidium spp.	337·8 (155·3; 614·5)	0·957 (0·398; 1·97)	87·8 (36·6; 180·7)	193·6 (78·5; 348·4)	0·068 (0·027; 0·139)	2·43 (1·08; 4·64)
Cyclospora cayetanensis	101·0 (-8·07; 309·5)	0·892 (0·176; 2·84)	81·6 (16·1; 259·8)	132·6 (-10·3; 423·1)	0·119 (0·026; 0·358)	4·24 (0·931; 12·9)
Entamoeba histolytica	26·5 (4·10; 80·5)	0·107 (0·025; 0·268)	9·80 (2·29; 24·6)	62·3 (8·15; 204·3)	0·060 (0·007; 0·197)	1·62 (0·224; 4·92)
Enteroaggregative *E. coli*	148·4 (-1,420·0; 1,714·6)	0·755 (-1·32; 3·13)	69·2 (-120·0; 286·5)	112·4 (-1,120·0; 1,221·9)	0·109 (-0·180; 0·454)	3·25 (-5·05; 13·4)
Enteropathogenic *E. coli*	429·0 (11·5; 953·1)	0·623 (-0·111; 1·58)	57·5 (-9·78; 145·5)	262·2 (7·09; 603·4)	0·056 (-0·009; 0·151)	2·09 (-0·137; 5·05)
Enterotoxigenic *E. coli*	1,876·0 (1,086·5; 2,912·1)	1·86 (0·904; 3·32)	172·2 (84·5; 306·0)	1,644·5 (883·5; 2,774·8)	0·226 (0·109; 0·418)	8·72 (4·57; 15·1)
Giardia duodenalis	436·4 (165·4; 906·7)	0 (0; 0)	0·561 (0·189; 1·27)	347·3 (98·6; 812·8)	0 (0; 0)	0·229 (0·058; 0·567)

Norovirus	1,128·5 (661·1; 1,848·8)	0·824 (0·349; 1·51)	77·1 (33·1; 140·2)	654·9 (322·3; 1,194·4)	0·118 (0·037; 0·263)	3·99 (1·58; 8·09)
Rotavirus	960·8 (393·8; 1,700·7)	2·48 (0·501; 4·94)	228·0 (46·1; 453·2)	262·8 (71·5; 552·0)	0·128 (0·031; 0·308)	4·14 (1·02; 9·13)
Salmonella enterica, non-typhoidal	373·5 (212·9; 601·2)	1·28 (0·686; 2·29)	117·1 (63·1; 209·5)	287·9 (136·0; 538·9)	0·208 (0·109; 0·368)	6·61 (3·79; 11·3)
Shiga toxin-producing *E. coli*	721·6 (334·4; 1,439·0)	1·07 (0·445; 2·20)	99·6 (41·4; 203·4)	652·8 (256·9; 1,432·2)	0·184 (0·069; 0·417)	7·92 (3·16; 17·5)
Shigella spp.	1,333·3 (698·8; 2,440·2)	2·11 (1·07; 3·62)	194·7 (99·2; 332·7)	1,511·6 (626·5; 2,937·9)	0·393 (0·137; 0·791)	13·0 (5·36; 24·8)
Vibrio cholerae	80·6 (23·8; 201·1)	0·371 (0·135; 0·769)	34·2 (12·4; 71·5)	312·3 (72·5; 912·3)	0·262 (0·061; 0·730)	8·20 (2·22; 20·8)
**TOTAL**	10,701·5 (7,820·3; 13,815·5)	15·6 (11·5; 20·2)	1,441·8 (1,061·8; 1,863·5)	8,238·5 (5,902·5; 11,110·6)	2·21 (1·53; 3·07)	76·3 (55·4; 100·4)

* and other thermotolerant species

Table S2. **Mean rates of foodborne illnesses, deaths, and Disability-Adjusted Life Years (DALYs) per 100,000 persons by 14 diarrhoeal enteric hazards, globally and by World Health Organization Region*, with 95% uncertainty intervals (UIs), 2021.**

(a) African Region, Region of the Americas, South-East Asia Region, European Region

PATHOGEN	African Region			Region of the Americas			South-East Asia Region			European Region		
	ILLNESSES (95% UI)	DEATHS (95% UI)	DALYs (95% UI)	ILLNESSES (95% UI)	DEATHS (95% UI)	DALYs (95% UI)	ILLNESSES (95% UI)	DEATHS (95% UI)	DALYs (95% UI)	ILLNESSES (95% UI)	DEATHS (95% UI)	DALYs (95% UI)
*Campylobacter jejuni/coli***	3,184·5 (1,503·5; 5,163·0)	1·13 (0·500; 2·02)	87·4 (38·5; 153·8)	661·8 (377·3; 1,027·1)	0·072 (0·036; 0·123)	4·65 (2·48; 7·55)	3,395·7 (1,462·8; 6,382·0)	0·715 (0·273; 1·41)	27·7 (11·5; 54·4)	401·9 (253·1; 599·4)	0·062 (0·029; 0·108)	3·79 (1·98; 6·37)
Cryptosporidium spp.	756·7 (286·5; 1,461·0)	0·684 (0·270; 1·41)	52·7 (210·0; 110·9)	42·4 (9·54; 104·0)	0·008 (0·002; 0·019)	0·464 (0·132; 1·03)	246·3 (29·6; 661·7)	0·122 (0·015; 0·335)	4·40 (0·534; 11·9)	26·4 (5·92; 67·0)	0·002 (<0.001; 0·006)	0·180 (0·046; 0·438)
Cyclospora cayetanensis	455·2 (-30·7; 1,741·5)	0·825 (0·168; 2·38)	57·6 (11·2; 168·5)	31·1 (0·141; 110·4)	0·018 (0·003; 0·059)	0·735 (0·128; 2·44)	136·0 (-10·0; 558·3)	0·173 (0·027; 0·663)	5·06 (0·878; 18·7)	24·5 (2·58; 85·5)	0·004 (<0.001; 0·012)	0·197 (0·033; 0·645)
Entamoeba histolytica	62·3 (6·11; 216·7)	0·078 (0·012; 0·250)	4·84 (0·687; 15·1)	14·2 (2·01; 46·3)	0·006 (<0.001; 0·021)	0·262 (0·035; 0·826)	161·3 (2·90; 605·5)	0·182 (0·005; 0·634)	5·18 (0·156; 17·9)	8·20 (0·827; 26·3)	<0.001 (<0.001; 0·003)	0·053 (0·008; 0·167)
Enteroaggregative *E. coli*	201·5 (-1,990·0; 2,396·6)	0·388 (-0·700; 1·67)	28·0 (-50·2; 122·6)	27·6 (-286·0; 330·7)	0·022 (-0·038; 0·099)	1·09 (-1·92; 4·88)	222·2 (-2,300·0; 2,506·7)	0·289 (-0·464; 1·21)	9·90 (-15·0; 41·3)	7·54 (-72·2; 96·6)	0·003 (-0·005; 0·014)	0·196 (-0·321; 0·903)
Enteropathogenic *E. coli*	889·4 (28·6; 2,054·5)	0·423 (-0·076; 1·06)	30·9 (-4·54; 78·6)	45·5 (1·01; 130·9)	0·008 (-0·001; 0·025)	0·439 (-0·022; 1·34)	269·9 (4·98; 804·4)	0·083 (-0·011; 0·260)	2·97 (-0·179; 8·76)	33·4 (0·717; 94·1)	0·002 (0·000; 0·007)	0·184 (-0·006; 0·524)

Enterotoxigenic *E. coli*	5,510·9 (3,062·3; 9,178·5)	1·46 (0·689; 2·60)	105·0 (50·1; 186·5)	262·9 (81·9; 550·5)	0·025 (0·006; 0·057)	1·30 (0·362; 2·78)	1,776·8 (739·7; 3,682·1)	0·348 (0·119; 0·864)	12·0 (4·17; 28·2)	178·6 (61·0; 340·8)	0·006 (0·002; 0·015)	0·504 (0·165; 1·11)
Giardia duodenalis	809·9 (115·0; 2,224·5)	0 (0; 0)	0·599 (0·094; 1·73)	83·4 (11·5; 298·9)	0 (0; 0)	0·062 (0·008; 0·228)	591·4 (21·7; 1,844·7)	0 (0; 0)	0·411 (0·016; 1·36)	51·6 (7·72; 145·7)	0 (0; 0)	0·038 (0·005; 0·105)
Norovirus	754·5 (228·5; 1,674·2)	0·412 (0·124; 0·933)	31·4 (9·46; 70·7)	662·2 (164·0; 1,753·3)	0·038 (0·013; 0·080)	2·64 (0·901; 5·54)	1,006·6 (95·0; 2,823·0)	0·321 (0·034; 0·889)	11·9 (1·22; 31·4)	827·7 (146·0; 1,799·7)	0·050 (0·008; 0·129)	3·57 (0·621; 8·64)
Rotavirus	929·0 (117·0; 1,943·0)	1·49 (0·194; 3·12)	122·5 (15·8; 256·9)	74·4 (13·3; 177·4)	0·015 (0·001; 0·043)	1·03 (0·113; 2·79)	520·4 (25·6; 1,591·5)	0·357 (0·017; 1·12)	15·2 (0·717; 46·3)	63·4 (15·4; 213·9)	0·006 (0·001; 0·020)	0·510 (0·129; 1·62)
Salmonella enterica, non-typhoidal	548·4 (231·3; 1,136·5)	0·871 (0·438; 1·67)	62·6 (30·7; 122·1)	235·2 (135·6; 360·6)	0·071 (0·044; 0·112)	3·77 (2·40; 5·73)	469·0 (145·7; 1,028·6)	0·484 (0·209; 0·979)	15·5 (6·86; 29·5)	155·2 (94·6; 250·3)	0·070 (0·041; 0·110)	4·12 (2·46; 6·38)
Shiga-toxin producing *E. coli*	1,572·8 (578·9; 3,562·0)	0·805 (0·298; 1·76)	58·7 (21·7; 128·7)	170·9 (58·0; 380·5)	0·048 (0·019; 0·106)	3·04 (1·27; 6·58)	1,112·3 (291·9; 3,196·8)	0·387 (0·101; 1·01)	16·3 (4·37; 41·5)	87·9 (18·1; 261·1)	0·022 (0·006; 0·054)	1·58 (0·428; 3·91)
Shigella spp.	3,475·6 (1,629·0; 6,289·3)	1·58 (0·683; 2·73)	111·4 (48·2; 197·6)	211·5 (49·8; 592·9)	0·038 (0·010; 0·093)	1·90 (0·507; 4·38)	2,901·7 (444·7; 7,167·8)	0·955 (0·124; 2·37)	31·8 (4·24; 75·7)	46·4 (11·8; 109·5)	0·004 (0·001; 0·009)	0·288 (0·077; 0·675)
Vibrio cholerae	315·0 (80·0; 793·9)	0·331 (0·090; 0·742)	19·9 (5·50; 43·6)	0·539 (0·052; 1·75)	0·002 (<0.001; 0·005)	0·087 (0·010; 0·280)	801·9 (93·2; 2,603·9)	0·750 (0·084; 2·33)	22·8 (2·59; 67·8)	0 (0; 0)	0 (0; 0)	0 (0; 0)
**TOTAL**	19,465·7 (13,686·9; 25,363·6)	10·5 (7·60; 14·0)	773·5 (559·7; 1,033·3)	2,523·6 (1,658·0; 3,835·4)	0·372 (0·258; 0·502)	21·5 (15·2; 28·8)	13,611·6 (8,028·9; 20,655·5)	5·17 (3·08; 7·87)	181·2 (111·1; 271·9)	1,912·6 (1,117·6; 2,941·9)	0·231 (0·153; 0·333)	15·2 (10·3; 21·5)

(b) Eastern Mediterranean Region, Western Pacific Region, Global

PATHOGEN	Eastern Mediterranean Region			Western Pacific Region			GLOBAL		
	**ILLNESSES (95% UI)**	**DEATHS (95% UI)**	**DALYs (95% UI)**	**ILLNESSES (95% UI)**	**DEATHS (95% UI)**	**DALYs (95% UI)**	**ILLNESSES (95% UI)**	**DEATHS (95% UI)**	**DALYs (95% UI)**
*Campylobacter jejuni/coli***	3,055·3 (1,309·3; 5,478·8)	0·717 (0·276; 1·44)	54·4 (22·4; 104·5)	382·1 (226·5; 624·4)	0·037 (0·018; 0·069)	2·95 (1·52; 5·46)	1,882·8 (1,059·7; 3,038·1)	0·450 (0·219; 0·748)	27·2 (14·0; 44·6)
Cryptosporidium spp.	173·0 (27·3; 505·7)	0·093 (0·017; 0·240)	6·89 (1·32; 17·6)	17·5 (4·17; 45·1)	0·004 (<0.001; 0·012)	0·292 (0·053; 0·873)	206·0 (86·4; 359·7)	0·144 (0·061; 0·272)	9·76 (4·22; 18·8)
Cyclospora cayetanensis	154·6 (-12·9; 580·7)	0·137 (0·030; 0·396)	8·31 (1·90; 25·7)	20·6 (-0·301; 89·2)	0·009 (<0.001; 0·037)	0·499 (0·022; 1·92)	129·9 (-10·7; 403·3)	0·186 (0·042; 0·558)	10·9 (2·43; 29·1)
Entamoeba histolytica	38·5 (2·54; 155·1)	0·032 (0·003; 0·132)	1·90 (0·214; 7·81)	5·10 (0·269; 21·7)	0·002 (<0.001; 0·009)	0·105 (0·003; 0·468)	59·2 (7·85; 195·0)	0·064 (0·009; 0·198)	2·32 (0·437; 6·78)
Enteroaggregative *E. coli*	212·1 (-2,100·0; 2,696·7)	0·272 (-0·469; 1·17)	19·2 (-33·5; 83·3)	10·3 (-126·0; 152·1)	0·009 (-0·013; 0·046)	0·602 (-0·890; 2·99)	115·5 (-1,130·0; 1,259·4)	0·165 (-0·273; 0·676)	8·92 (-14·8; 37·1)
Enteropathogenic *E. coli*	610·1 (15·4; 1,528·1)	0·190 (-0·031; 0·485)	14·0 (-1·86; 36·1)	21·3 (0·274; 63·7)	0·003 (-0·001; 0·012)	0·246 (-0·020; 0·806)	276·5 (7·52; 625·5)	0·105 (-0·018; 0·264)	6·85 (-0·947; 17·0)
Enterotoxigenic *E. coli*	3,174·0 (1,527·6; 5,532·0)	0·546 (0·191; 1·14)	38·6 (14·0; 76·6)	85·5 (11·1; 190·8)	0·008 (<0.001; 0·021)	0·522 (0·066; 1·41)	1,664·4 (917·4; 2,796·7)	0·366 (0·186; 0·657)	22·8 (12·0; 38·5)
Giardia duodenalis	581·0 (58·9; 2,343·4)	0 (0; 0)	0·444 (0·044; 1·68)	29·1 (3·57; 83·9)	0 (0; 0)	0·023 (0·003; 0·068)	354·9 (105·3; 812·4)	0 (0; 0)	0·257 (0·068; 0·610)
Norovirus	517·4 (194·5; 1,094·8)	0·151 (0·053; 0·324)	11·4 (4·15; 24·8)	352·3 (167·0; 604·2)	0·035 (0·013; 0·066)	2·60 (0·918; 4·97)	695·6 (355·8; 1,233·1)	0·179 (0·068; 0·363)	10·3 (4·52; 19·6)
Rotavirus	44·6 (0·007; 369·1)	0·040 (<0.001; 0·263)	3·26 (<0.001; 20·8)	114·0 (5·06; 332·1)	0·041 (0·002; 0·123)	3·18 (0·139; 9·37)	322·7 (101·5; 641·2)	0·331 (0·078; 0·656)	23·4 (4·98; 45·7)

Salmonella enterica, non-typhoidal	200·0 (73·6; 421·1)	0·183 (0·079; 0·358)	12·8 (5·54; 25·1)	95·0 (47·3; 163·1)	0·038 (0·021; 0·064)	2·34 (1·30; 3·96)	295·3 (143·5; 536·6)	0·300 (0·166; 0·501)	16·1 (9·16; 27·5)
Shiga-toxin producing *E. coli*	974·1 (338·4; 2,323·1)	0·308 (0·104; 0·772)	22·2 (7·74; 55·6)	32·9 (5·03; 107·4)	0·008 (0·001; 0·024)	0·522 (0·090; 1·67)	658·7 (262·2; 1,428·8)	0·261 (0·110; 0·533)	15·8 (6·93; 30·6)
Shigella spp.	1,673·1 (220·2; 3,760·0)	0·483 (0·087; 1·09)	33·3 (6·27; 75·2)	116·0 (30·2; 274·5)	0·018 (0·004; 0·046)	1·21 (0·255; 3·14)	1,496·3 (623·3; 2,871·6)	0·540 (0·243; 1·02)	28·6 (14·4; 48·6)
Vibrio cholerae	364·9 (61·0; 1,170·3)	0·264 (0·054; 0·778)	15·4 (3·21; 44·4)	2·70 (0·171; 9·03)	0·002 (<0.001; 0·005)	0·100 (0·006; 0·310)	292·4 (68·4; 851·3)	0·271 (0·074; 0·735)	10·4 (3·20; 25·0)
**TOTAL**	11,772·7 (7,726·8; 16,580·2)	3·42 (2·27; 4·95)	242·1 (159·9; 355·9)	1,284·5 (874·2; 1,791·7)	0·214 (0·134; 0·320)	15·2 (9·31; 23·2)	8,450·1 (6,132·4; 11,218·3)	3·36 (2·48; 4·46)	193·6 (147·2; 243·0)

** and other thermotolerant species

* Countries by WHO Region, as of September 2023:

African Region: Algeria, Angola, Botswana, Burundi, Cameroon, Cabo Verde, Central African Republic, Chad, Comoros, Congo, Democratic Republic of the Congo, Benin, Equatorial Guinea, Ethiopia, Eritrea, Gabon, Gambia, Ghana, Guinea, Côte d'Ivoire, Kenya, Lesotho, Liberia, Madagascar, Malawi, Mali, Mauritania, Mauritius, Mozambique, Namibia, Niger, Nigeria, Guinea-Bissau, Rwanda, Sao Tome and Principe, Senegal, Seychelles, Sierra Leone, South Africa, Zimbabwe, South Sudan, Eswatini, Togo, Uganda, United Republic of Tanzania, Burkina Faso, Zambia
Region of the Americas: Antigua and Barbuda, Argentina, Bahamas, Barbados, Bolivia (Plurinational State of), Brazil, Belize, Canada, Chile, Colombia, Costa Rica, Cuba, Dominica, Dominican Republic, Ecuador, El Salvador, Grenada, Guatemala, Guyana, Haiti, Honduras, Jamaica, Mexico, Nicaragua, Panama, Paraguay, Peru, Saint Kitts and Nevis, Saint Lucia, Saint Vincent and the Grenadines, Suriname, Trinidad and Tobago, United States of America, Uruguay, Venezuela (Bolivarian Republic of)
South-East Asia Region: Bangladesh, Bhutan, Myanmar, Sri Lanka, India, Democratic People's Republic of Korea, Indonesia, Maldives, Nepal, Timor-Leste, Thailand

European Region: Albania, Andorra, Azerbaijan, Austria, Armenia, Belgium, Bosnia and Herzegovina, Bulgaria, Belarus, Croatia, Cyprus, Czechia, Denmark, Estonia, Finland, France, Georgia, Germany, Greece, Hungary, Iceland, Ireland, Israel, Italy, Kazakhstan, Kyrgyzstan, Latvia, Lithuania, Luxembourg, Malta, Monaco, Republic of Moldova, Montenegro, Netherlands (Kingdom of the), Norway, Poland, Portugal, Romania, Russian Federation, San Marino, Serbia, Slovakia, Slovenia, Spain, Sweden, Switzerland, Tajikistan, Türkiye, Turkmenistan, Ukraine, North Macedonia, United Kingdom of Great Britain and Northern Ireland, Uzbekistan
Eastern Mediterranean Region: Afghanistan, Bahrain, Djibouti, Iran (Islamic Republic of), Iraq, Jordan, Kuwait, Lebanon, Libya, Morocco, Oman, Pakistan, Qatar, Saudi Arabia, Somalia, Sudan, Syrian Arab Republic, United Arab Emirates, Tunisia, Egypt, Yemen
Western Pacific Region: Australia, Brunei Darussalam, Cambodia, China, Cook Islands, Fiji, Japan, Kiribati, Lao People's Democratic Republic, Malaysia, Marshall Islands, Micronesia (Federated States of), Mongolia, Nauru, New Zealand, Niue, Palau, Papua New Guinea, Philippines, Republic of Korea, Samoa, Singapore, Solomon Islands, Tonga, Tuvalu, Vanuatu, Viet Nam

Figure S1. **Annual foodborne diarrhoeal incidence per 100,000 persons, by enteric hazard, globally and by World Health Organization Region*, 2000-2021**

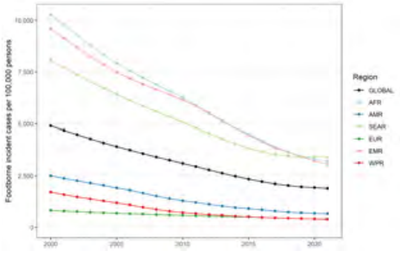

Campylobacter

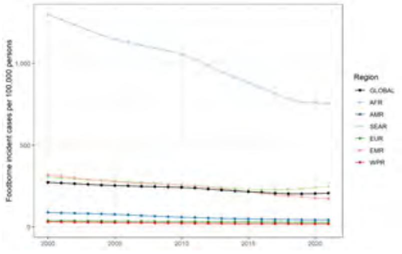

Cryptosporidium

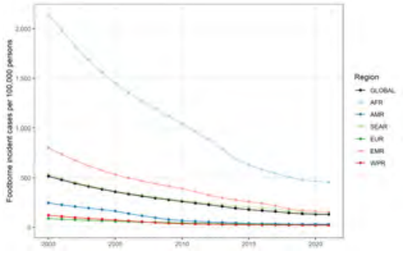

Cyclospora

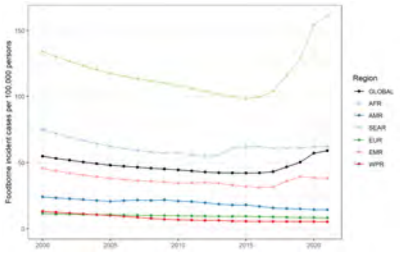

E. histolytica

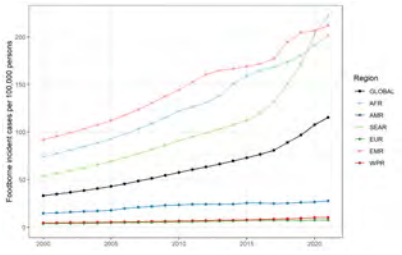

EAEC

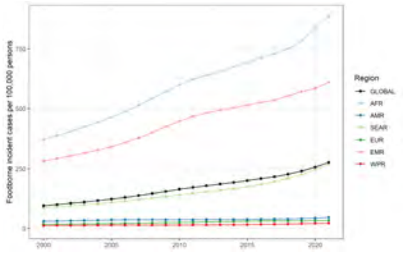

EPEC

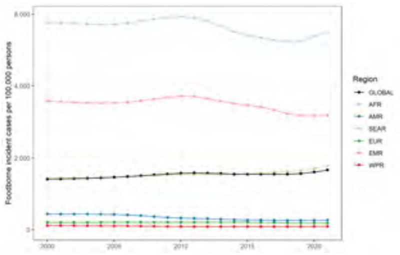

ETEC

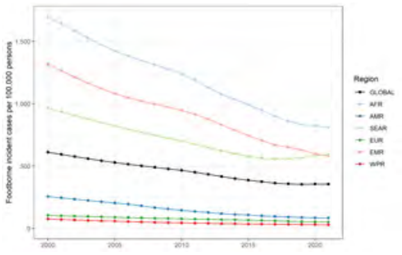

Giardia

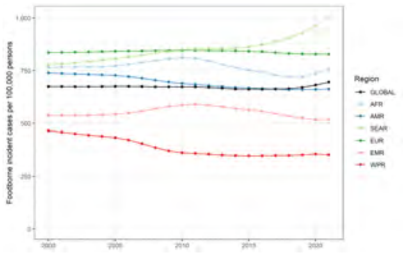

Norovirus

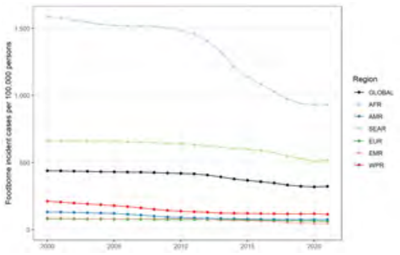

Rotavirus

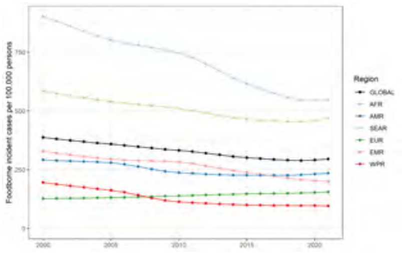

NTS, diarrhoeal

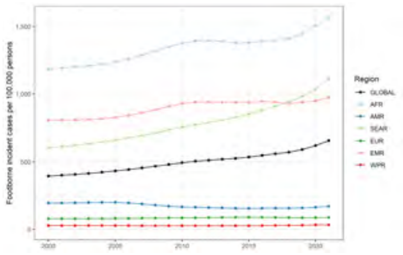

STEC

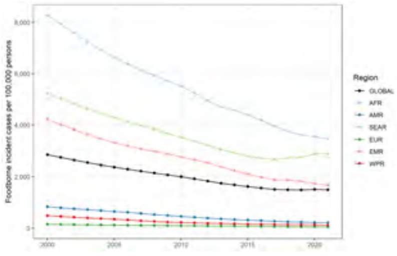

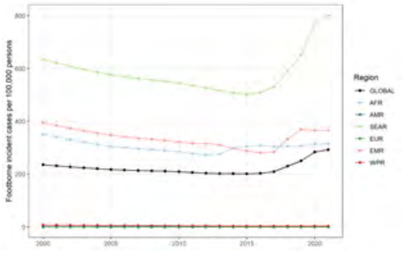

Shigella ***V. cholerae***

** Countries by WHO Region, as of September 2023:

African Region: Algeria, Angola, Botswana, Burundi, Cameroon, Cabo Verde, Central African Republic, Chad, Comoros, Congo, Democratic Republic of the Congo, Benin, Equatorial Guinea, Ethiopia, Eritrea, Gabon, Gambia, Ghana, Guinea, Côte d'Ivoire, Kenya, Lesotho, Liberia, Madagascar, Malawi, Mali, Mauritania, Mauritius, Mozambique, Namibia, Niger, Nigeria, Guinea-Bissau, Rwanda, Sao Tome and Principe, Senegal, Seychelles, Sierra Leone, South Africa, Zimbabwe, South Sudan, Eswatini, Togo, Uganda, United Republic of Tanzania, Burkina Faso, Zambia
Region of the Americas: Antigua and Barbuda, Argentina, Bahamas, Barbados, Bolivia (Plurinational State of), Brazil, Belize, Canada, Chile, Colombia, Costa Rica, Cuba, Dominica, Dominican Republic, Ecuador, El Salvador, Grenada, Guatemala, Guyana, Haiti, Honduras, Jamaica, Mexico, Nicaragua, Panama, Paraguay, Peru, Saint Kitts and Nevis, Saint Lucia, Saint Vincent and the Grenadines, Suriname, Trinidad and Tobago, United States of America, Uruguay, Venezuela (Bolivarian Republic of)
South-East Asia Region: Bangladesh, Bhutan, Myanmar, Sri Lanka, India, Democratic People's Republic of Korea, Indonesia, Maldives, Nepal, Timor-Leste, Thailand
European Region: Albania, Andorra, Azerbaijan, Austria, Armenia, Belgium, Bosnia and Herzegovina, Bulgaria, Belarus, Croatia, Cyprus, Czechia, Denmark, Estonia, Finland, France, Georgia, Germany, Greece, Hungary, Iceland, Ireland, Israel, Italy, Kazakhstan, Kyrgyzstan, Latvia, Lithuania, Luxembourg, Malta, Monaco, Republic of Moldova, Montenegro, Netherlands (Kingdom of the), Norway, Poland, Portugal, Romania, Russian Federation, San Marino, Serbia, Slovakia, Slovenia, Spain, Sweden, Switzerland, Tajikistan, Türkiye, Turkmenistan, Ukraine, North Macedonia, United Kingdom of Great Britain and Northern Ireland, Uzbekistan
Eastern Mediterranean Region: Afghanistan, Bahrain, Djibouti, Iran (Islamic Republic of), Iraq, Jordan, Kuwait, Lebanon, Libya, Morocco, Oman, Pakistan, Qatar, Saudi Arabia, Somalia, Sudan, Syrian Arab Republic, United Arab Emirates, Tunisia, Egypt, Yemen
Western Pacific Region: Australia, Brunei Darussalam, Cambodia, China, Cook Islands, Fiji, Japan, Kiribati, Lao People's Democratic Republic, Malaysia, Marshall Islands, Micronesia (Federated States of), Mongolia, Nauru, New Zealand, Niue, Palau, Papua New

Guinea, Philippines, Republic of Korea, Samoa, Singapore, Solomon Islands, Tonga, Tuvalu, Vanuatu, Viet Nam

Figure S2. Rank of 14 diarrhoeal disease hazards by mean total Disability-Adjusted Life Years caused globally, from (a) all routes of transmission and (b) foodborne transmission, in 2000 versus 2021

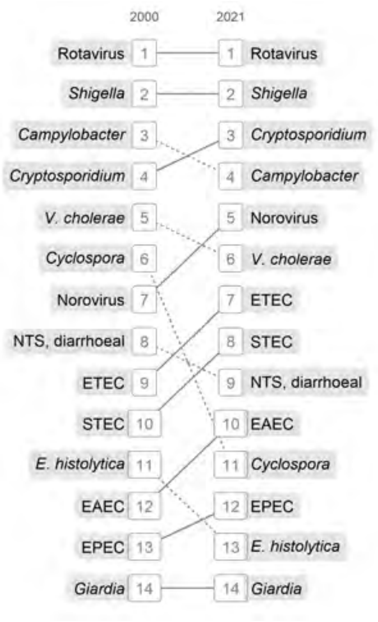

a. All routes of transmission

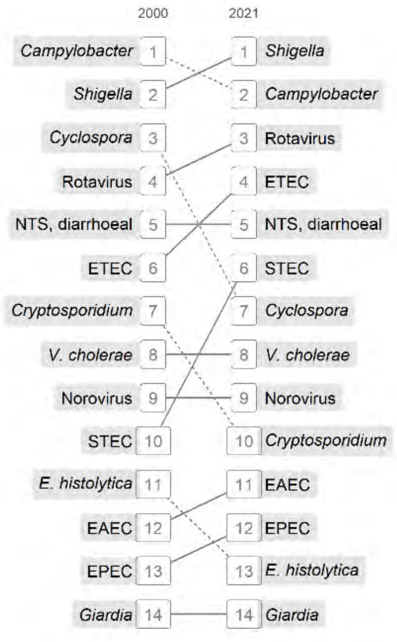

b. Foodborne transmission

Figure S3. Mean global Disability-Adjusted Life Years (DALYs) per 100,000 persons, caused by 14 diarrhoeal disease hazards, from (a) all routes of transmission and (b) foodborne transmission, for the period 2000 to 2021 (data in appendix 2)

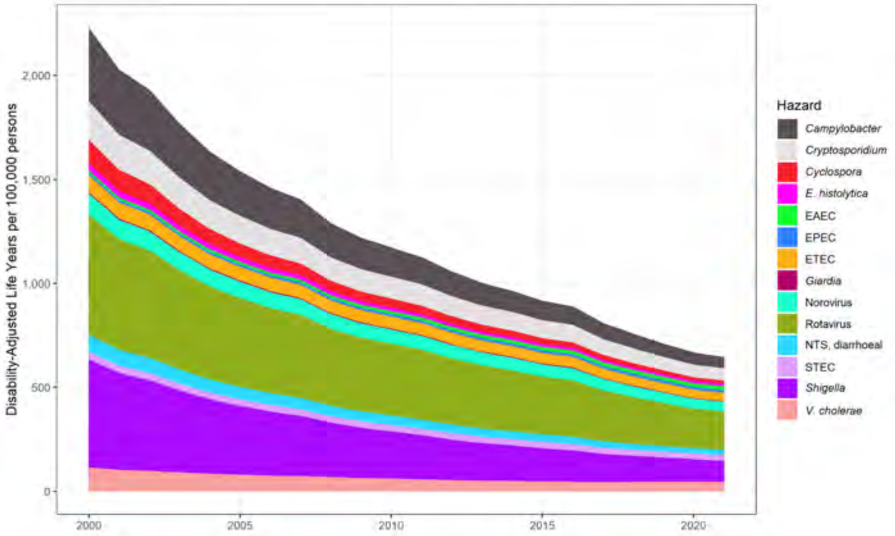

a. All routes of transmission

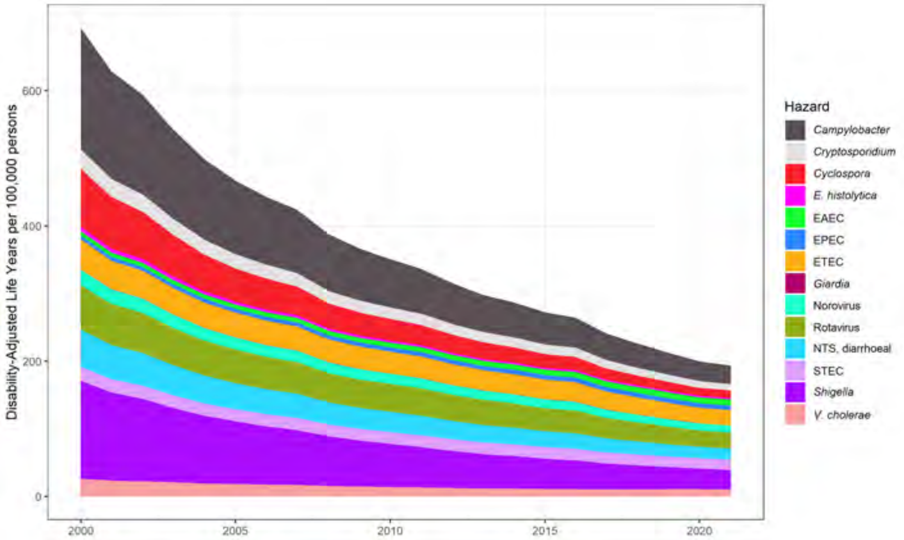

b. Foodborne transmission

Figure S4. **Rank of 14 diarrhoeal disease hazards by foodborne Disability-Adjusted Life Years caused, by World Health Organization Region*, 2021**

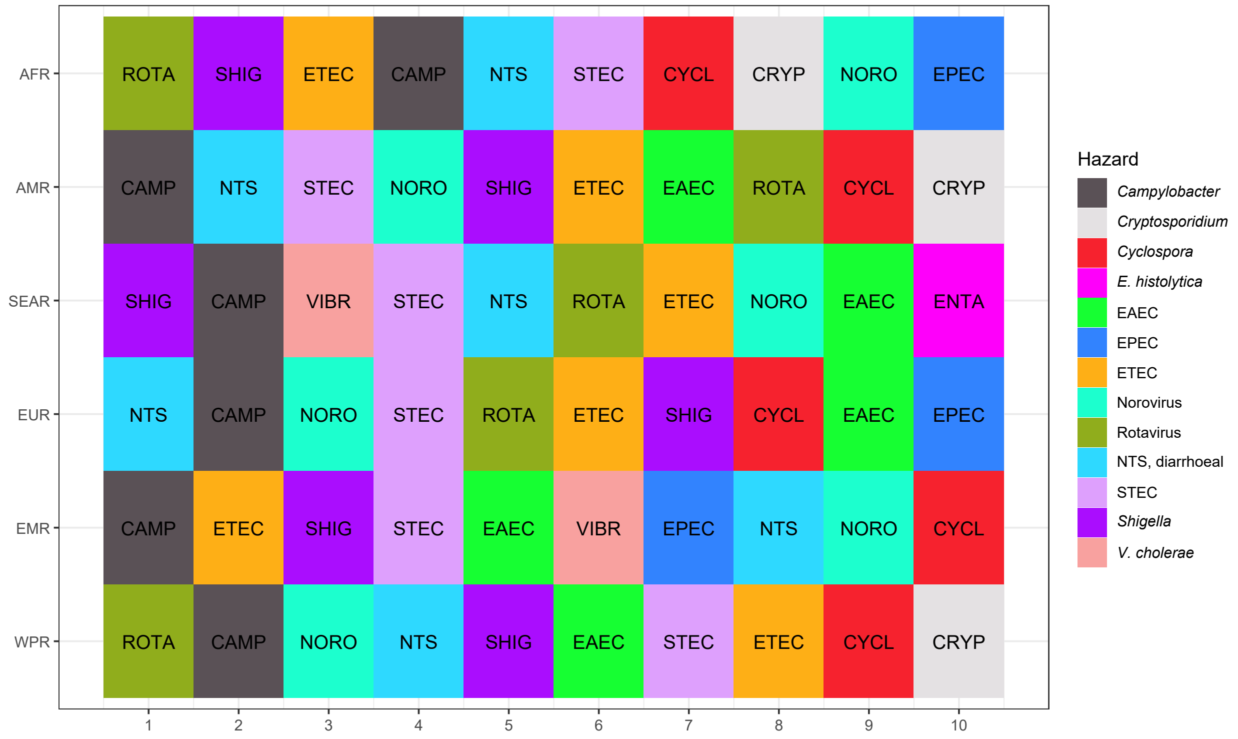

* Countries by WHO Region, as of September 2023:

African Region: Algeria, Angola, Botswana, Burundi, Cameroon, Cabo Verde, Central African Republic, Chad, Comoros, Congo, Democratic Republic of the Congo, Benin, Equatorial Guinea, Ethiopia, Eritrea, Gabon, Gambia, Ghana, Guinea, Côte d'Ivoire, Kenya, Lesotho, Liberia, Madagascar, Malawi, Mali, Mauritania, Mauritius, Mozambique, Namibia, Niger, Nigeria, Guinea-Bissau, Rwanda, Sao Tome and Principe, Senegal, Seychelles, Sierra Leone, South Africa, Zimbabwe, South Sudan, Eswatini, Togo, Uganda, United Republic of Tanzania, Burkina Faso, Zambia
Region of the Americas: Antigua and Barbuda, Argentina, Bahamas, Barbados, Bolivia (Plurinational State of), Brazil, Belize, Canada, Chile, Colombia, Costa Rica, Cuba, Dominica, Dominican Republic, Ecuador, El Salvador, Grenada, Guatemala, Guyana, Haiti, Honduras, Jamaica, Mexico, Nicaragua, Panama, Paraguay, Peru, Saint Kitts and Nevis, Saint Lucia, Saint Vincent and the Grenadines, Suriname, Trinidad and Tobago, United States of America, Uruguay, Venezuela (Bolivarian Republic of)
South-East Asia Region: Bangladesh, Bhutan, Myanmar, Sri Lanka, India, Democratic People's Republic of Korea, Indonesia, Maldives, Nepal, Timor-Leste, Thailand
European Region: Albania, Andorra, Azerbaijan, Austria, Armenia, Belgium, Bosnia and Herzegovina, Bulgaria, Belarus, Croatia, Cyprus, Czechia, Denmark, Estonia, Finland,

France, Georgia, Germany, Greece, Hungary, Iceland, Ireland, Israel, Italy, Kazakhstan, Kyrgyzstan, Latvia, Lithuania, Luxembourg, Malta, Monaco, Republic of Moldova, Montenegro, Netherlands (Kingdom of the), Norway, Poland, Portugal, Romania, Russian Federation, San Marino, Serbia, Slovakia, Slovenia, Spain, Sweden, Switzerland, Tajikistan, Türkiye, Turkmenistan, Ukraine, North Macedonia, United Kingdom of Great Britain and Northern Ireland, Uzbekistan
Eastern Mediterranean Region: Afghanistan, Bahrain, Djibouti, Iran (Islamic Republic of), Iraq, Jordan, Kuwait, Lebanon, Libya, Morocco, Oman, Pakistan, Qatar, Saudi Arabia, Somalia, Sudan, Syrian Arab Republic, United Arab Emirates, Tunisia, Egypt, Yemen
Western Pacific Region: Australia, Brunei Darussalam, Cambodia, China, Cook Islands, Fiji, Japan, Kiribati, Lao People's Democratic Republic, Malaysia, Marshall Islands, Micronesia (Federated States of), Mongolia, Nauru, New Zealand, Niue, Palau, Papua New Guinea, Philippines, Republic of Korea, Samoa, Singapore, Solomon Islands, Tonga, Tuvalu, Vanuatu, Viet Nam

Figure S5. **Mean national rates of foodborne Disability-Adjusted Life Years (DALYs) per 100,000 persons due to *Campylobacter jejuni/coli* and other thermotolerant species, 2021 (data in appendix 2)**

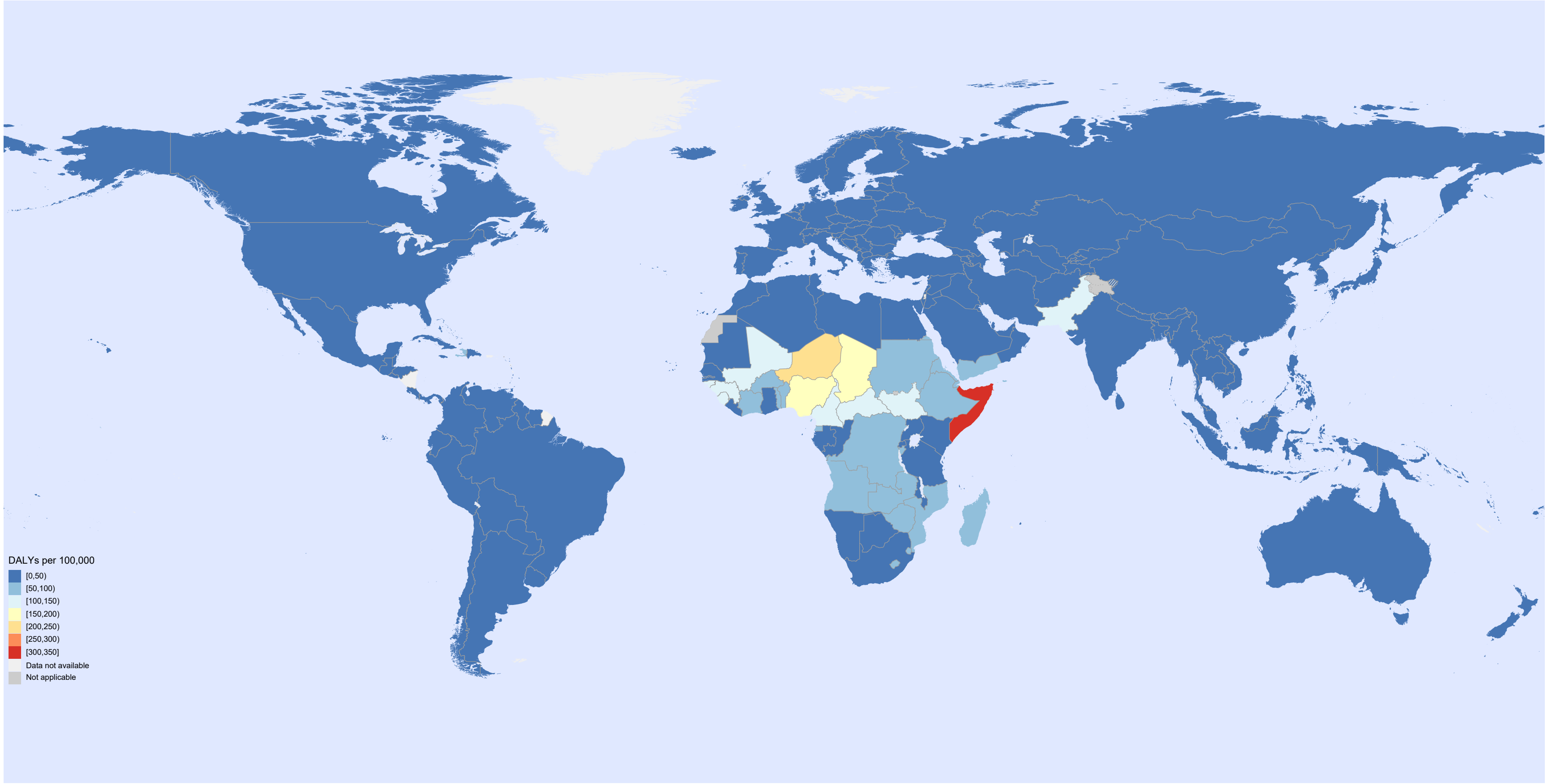

Figure S6. **Mean national rates of foodborne Disability-Adjusted Life Years (DALYs) per 100,000 persons due to *Cryptosporidium spp.*, 2021 (data in appendix 2)**

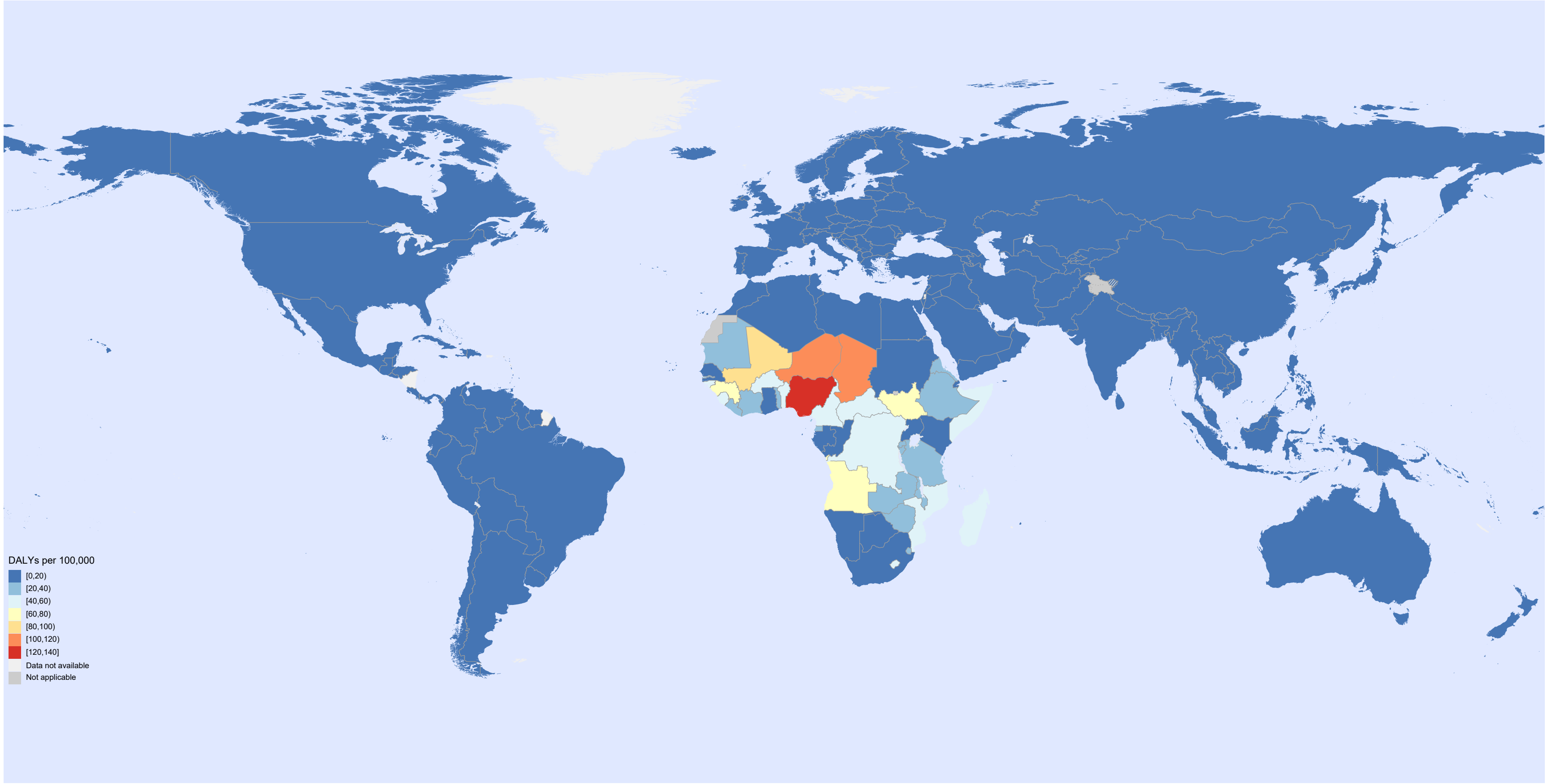

Figure S7. **Mean national rates of foodborne Disability-Adjusted Life Years (DALYs) per 100,000 persons due to *Cyclospora cayetanensis*, 2021 (data in appendix 2)**

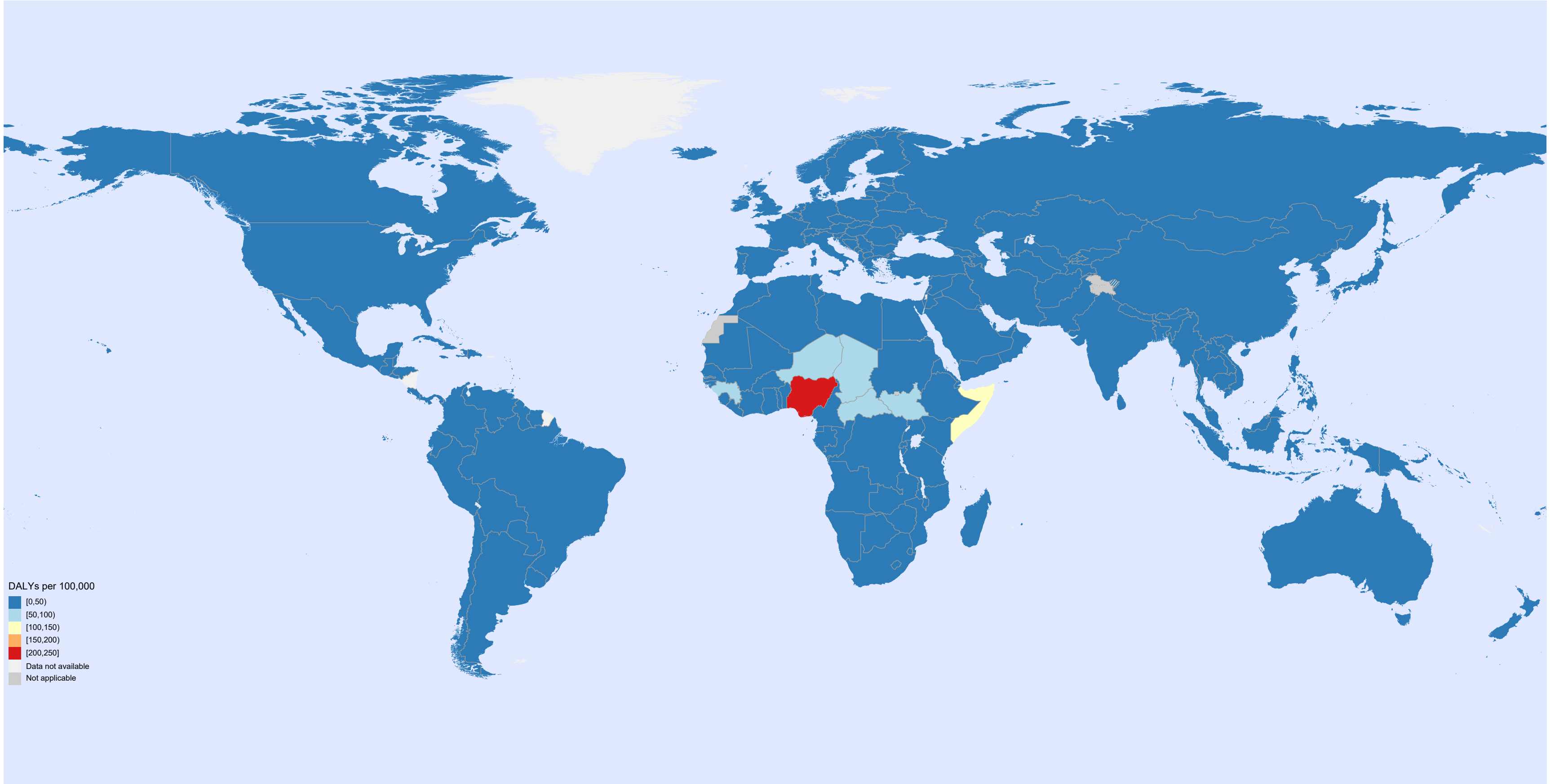

Figure S8. **Mean national rates of foodborne Disability-Adjusted Life Years (DALYs) per 100,000 persons due to *Entamoeba histolytica*, 2021 (data in appendix 2)**

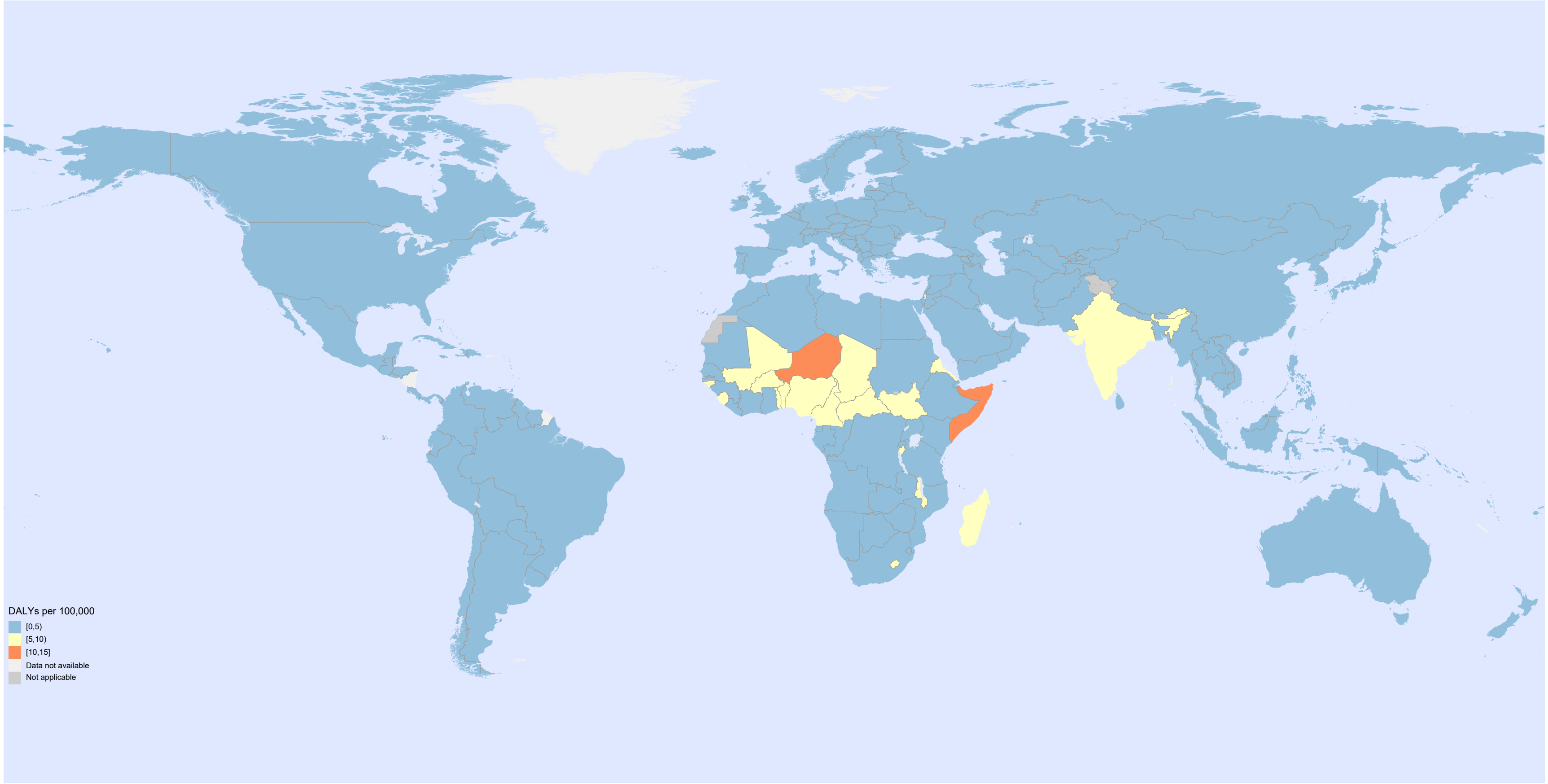

Figure S9. Mean national rates of foodborne Disability-Adjusted Life Years (DALYs) per 100,000 persons due to enteroaggregative *E. coli*, 2021 (data in appendix 2)

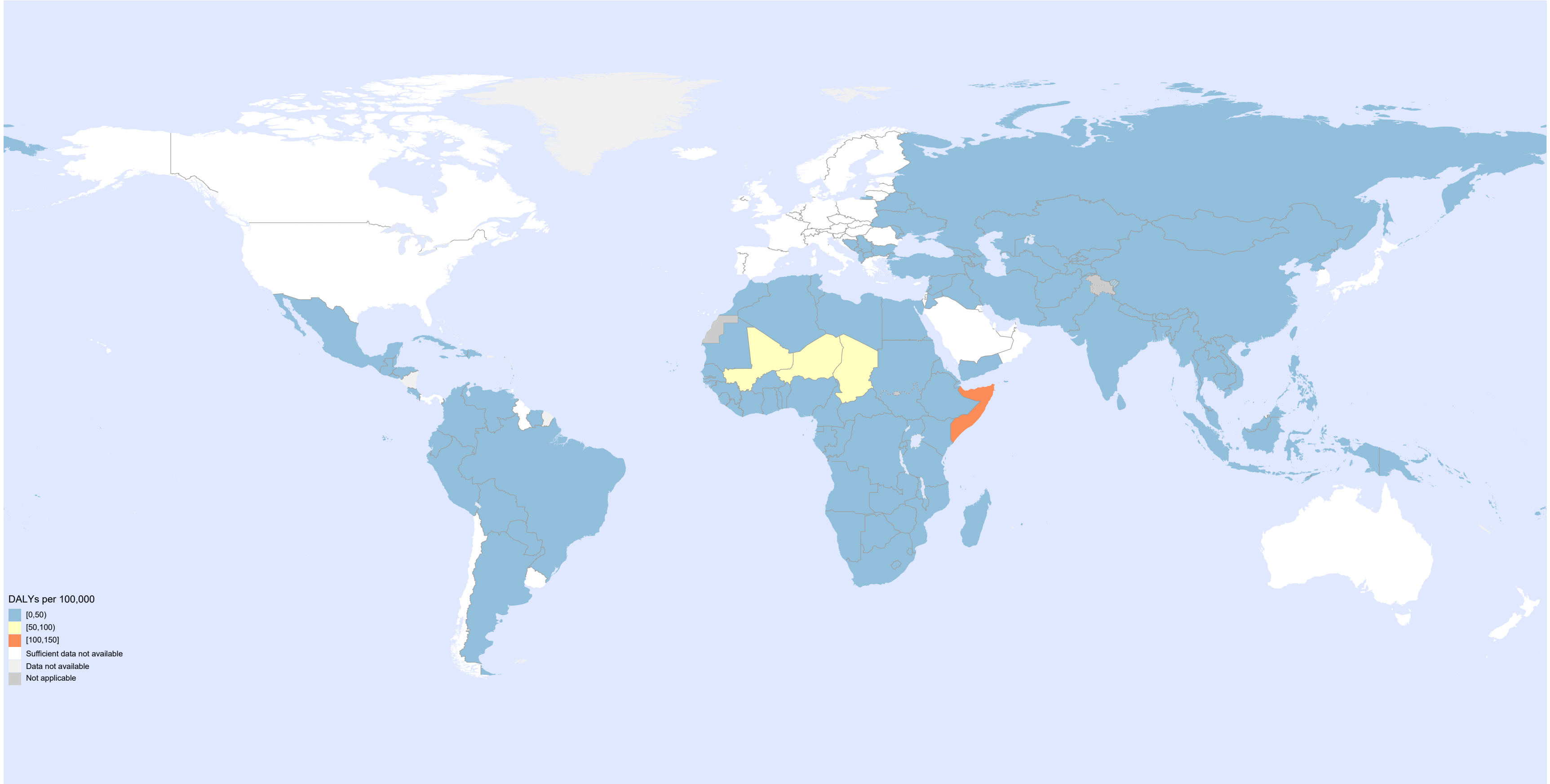

Figure S10. **Mean national rates of foodborne Disability-Adjusted Life Years (DALYs) per 100,000 persons due to enteropathogenic *E. coli*, 2021 (data in appendix 2)**

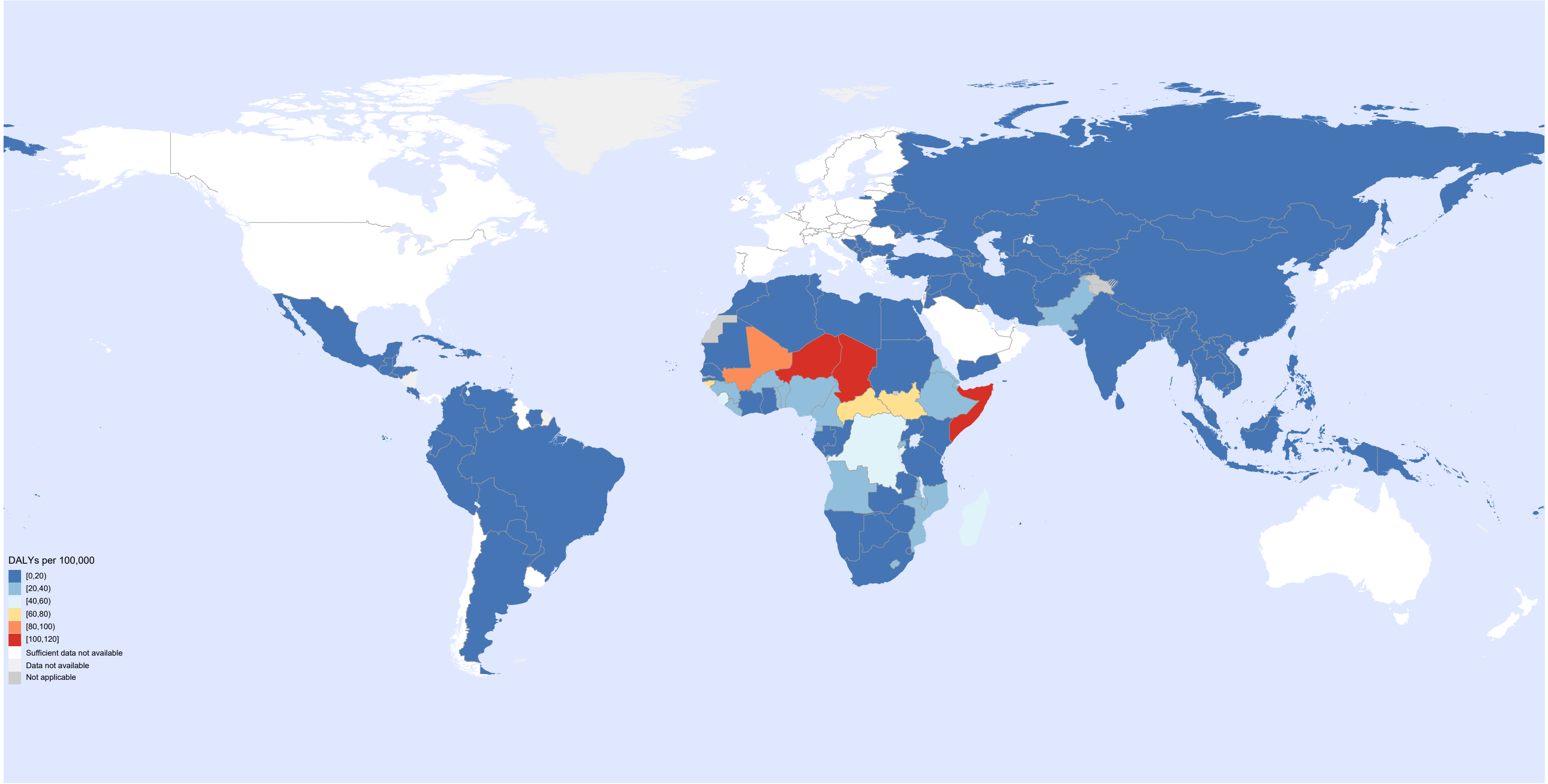

Figure S11. Mean national rates of foodborne Disability-Adjusted Life Years (DALYs) per 100,000 persons due to enterotoxigenic *E. coli*, 2021 (data in appendix 2)

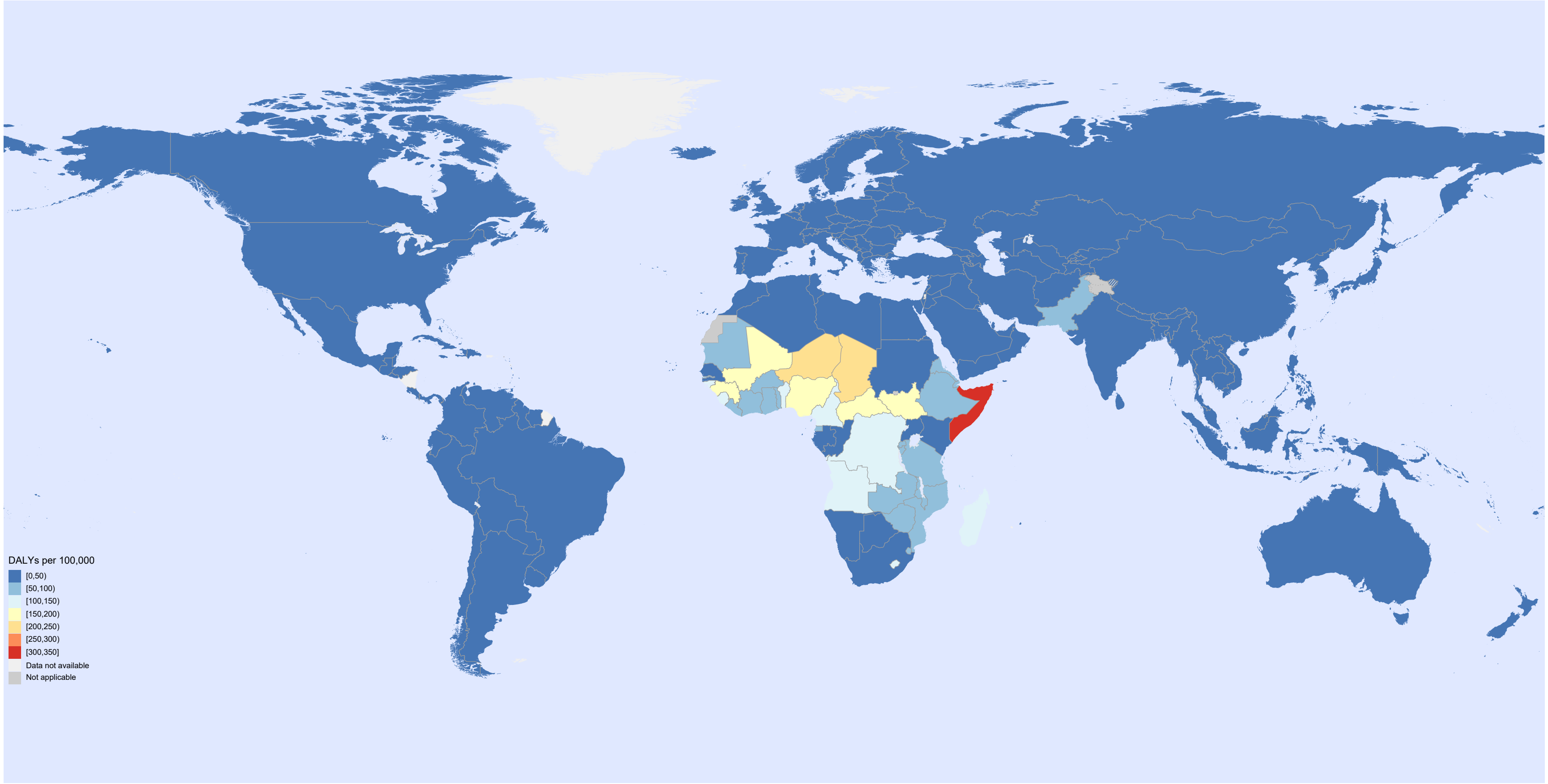

Figure S12. Mean national rates of foodborne Disability-Adjusted Life Years (DALYs) per 100,000 persons due to *Giardia duodenalis*, 2021 (data in appendix 2)

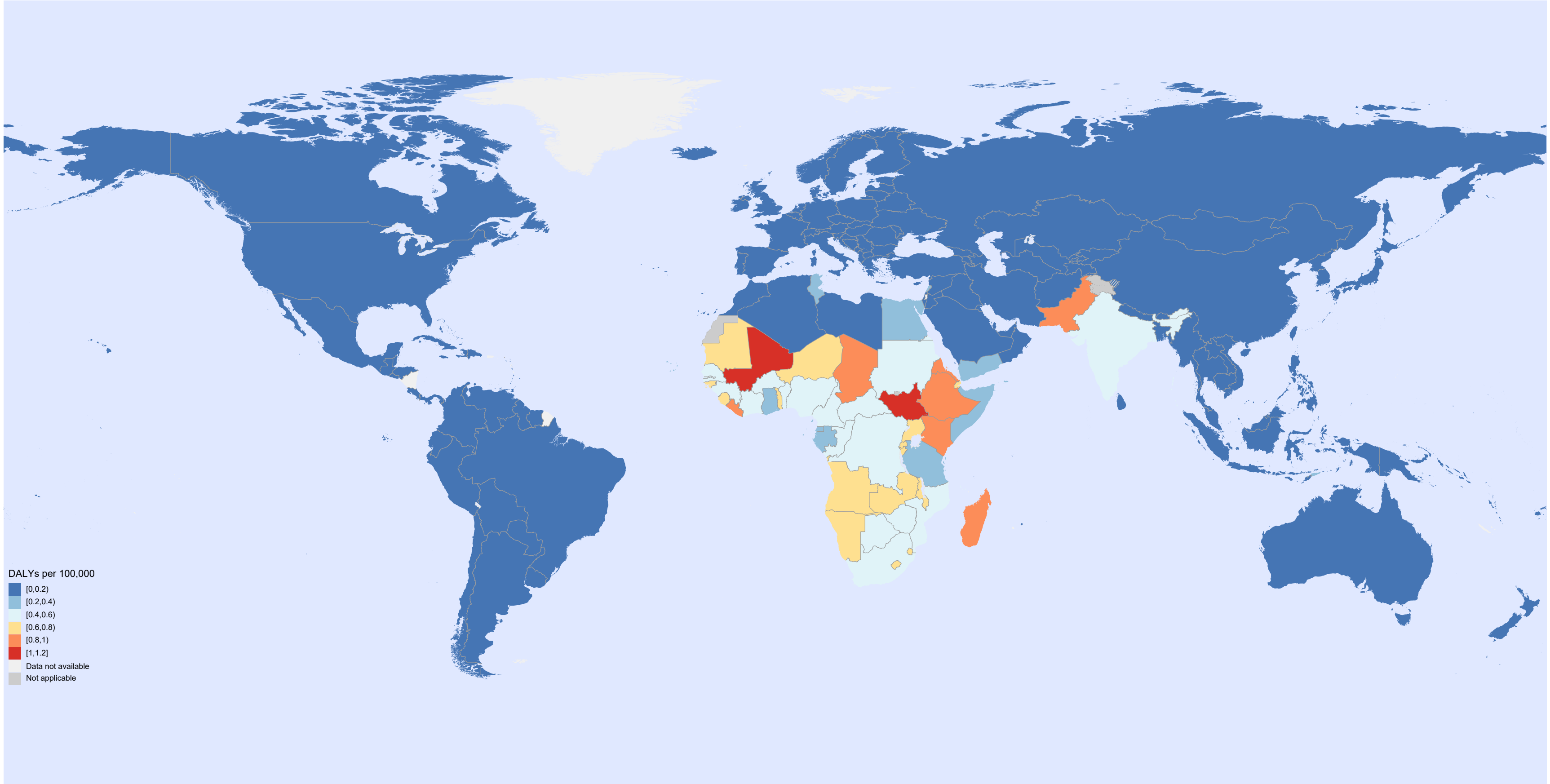

Figure S13. Mean national rates of foodborne Disability-Adjusted Life Years (DALYs) per 100,000 persons due to norovirus, 2021 (data in appendix 2)

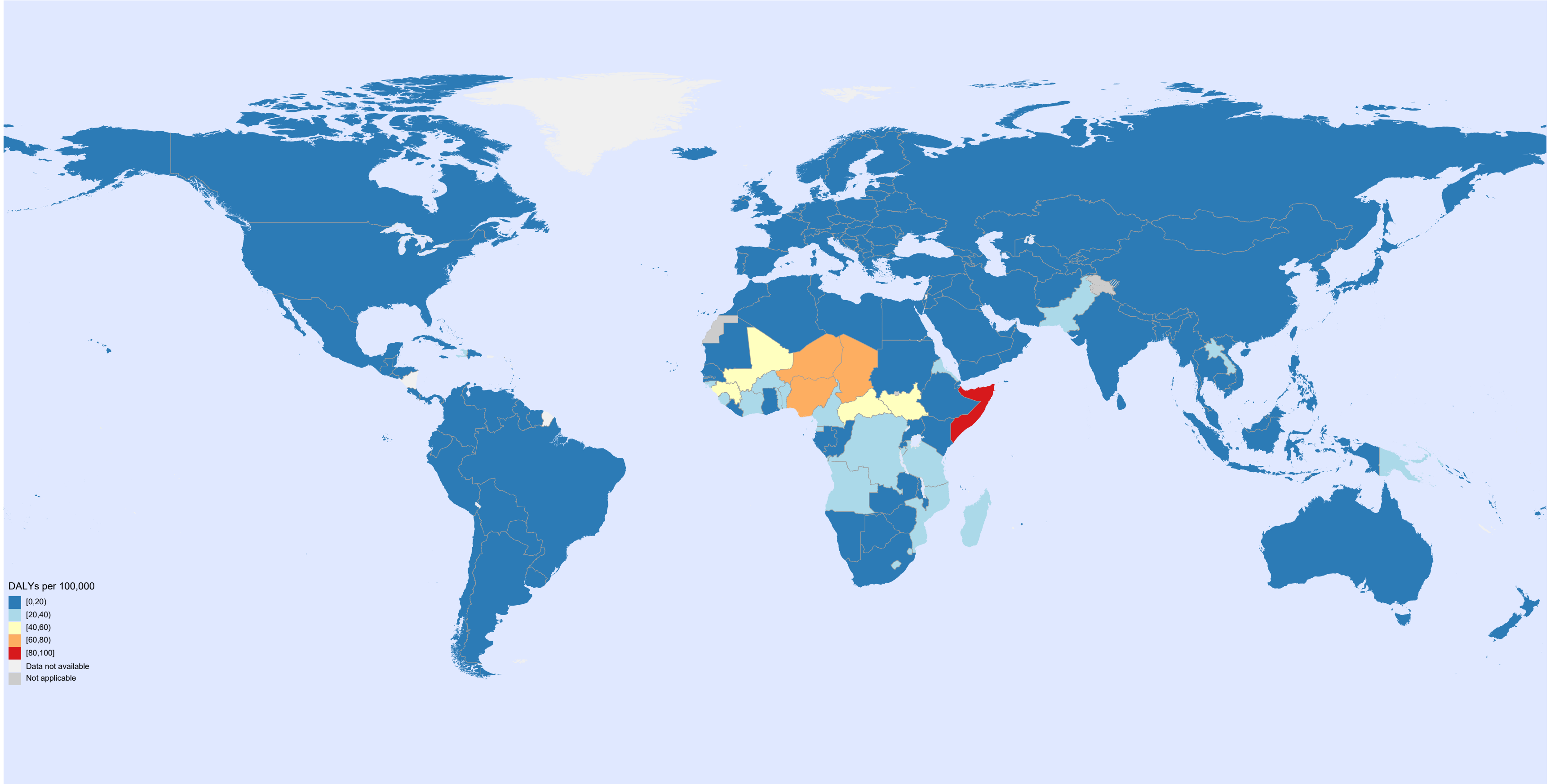

Figure S14. Mean national rates of foodborne Disability-Adjusted Life Years (DALYs) per 100,000 persons due to rotavirus, 2021 (data in appendix 2)

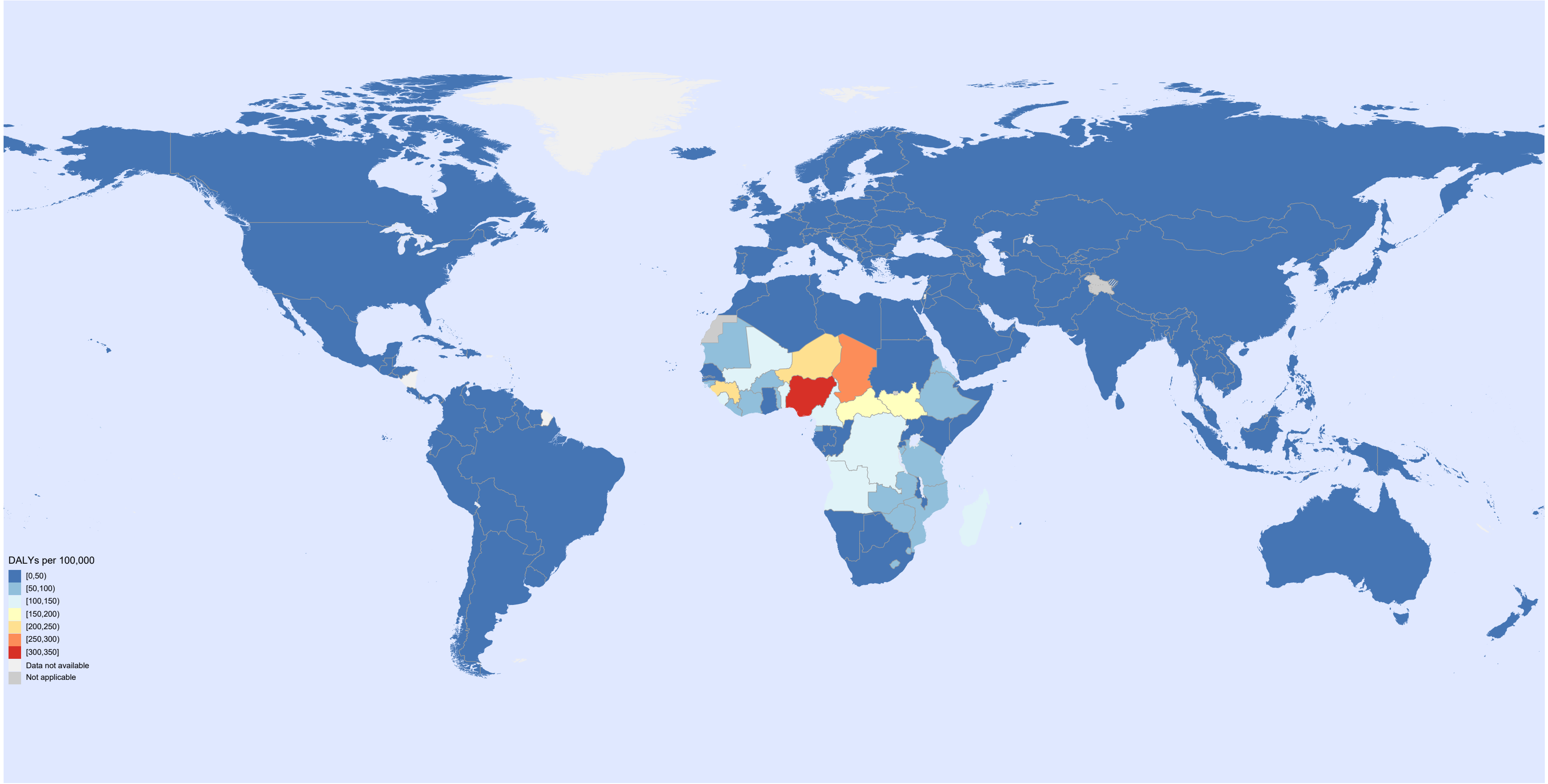

Figure S15. Mean national rates of foodborne Disability-Adjusted Life Years (DALYs) per 100,000 persons due to non-typhoidal *Salmonella enterica* diarrhoea, 2021 (data in appendix 2)

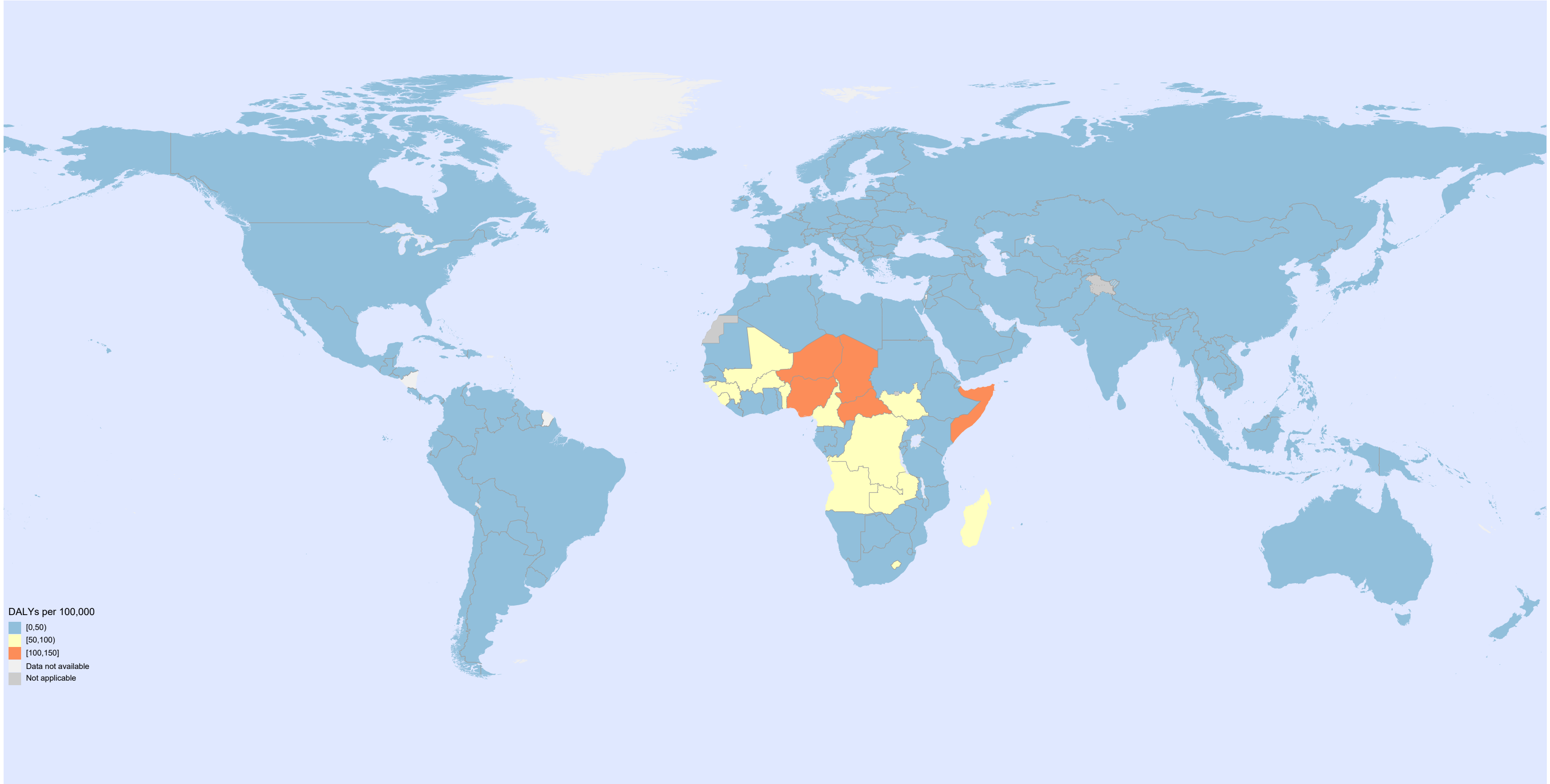

Figure S16. Mean national rates of foodborne Disability-Adjusted Life Years (DALYs) per 100,000 persons due to Shiga toxin- producing *E. coli*, 2021 (data in appendix 2)

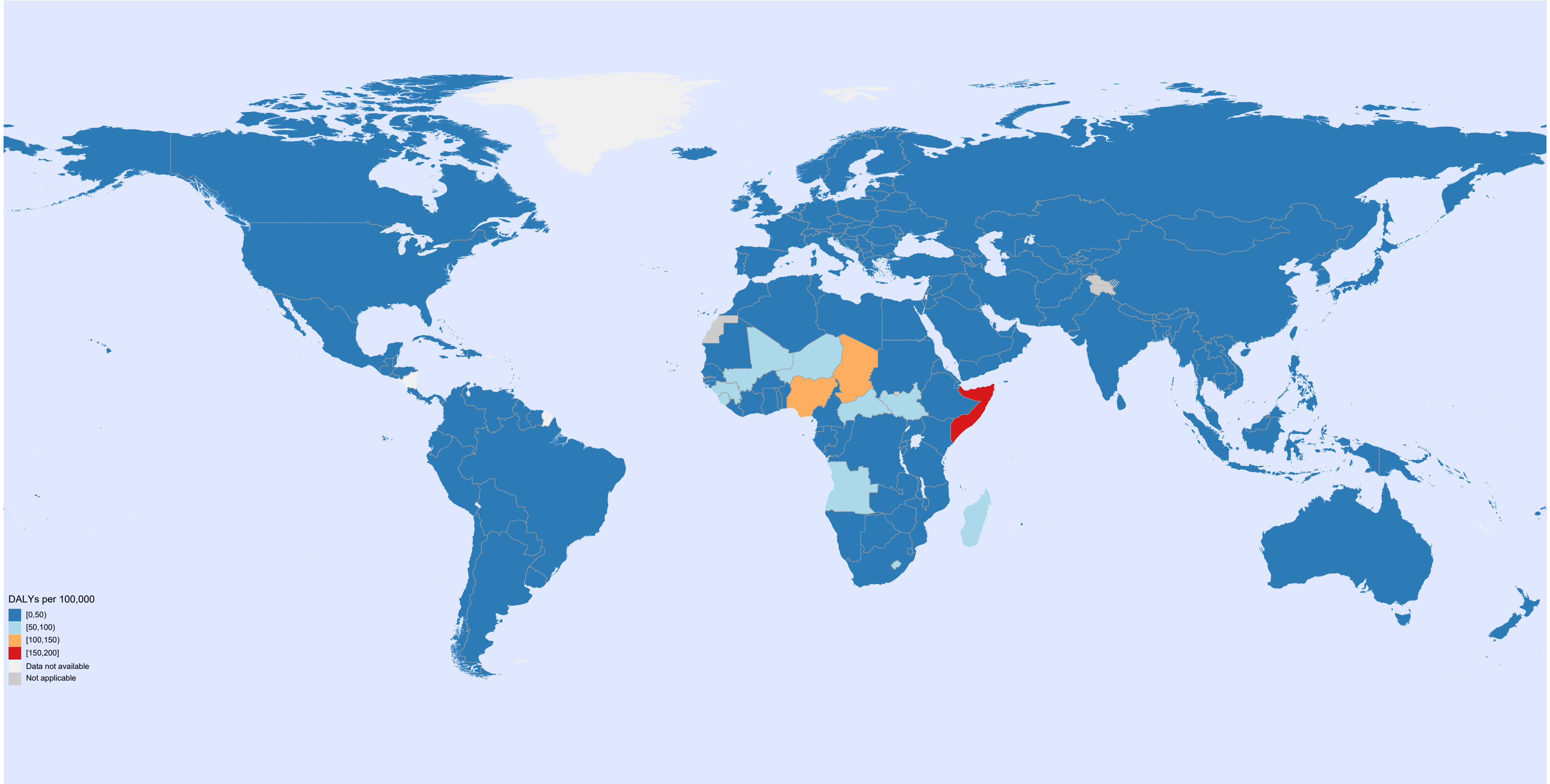

Figure S17. **Mean national rates of foodborne Disability-Adjusted Life Years (DALYs) per 100,000 persons due to *Shigella spp.*, 2021 (data in appendix 2)**

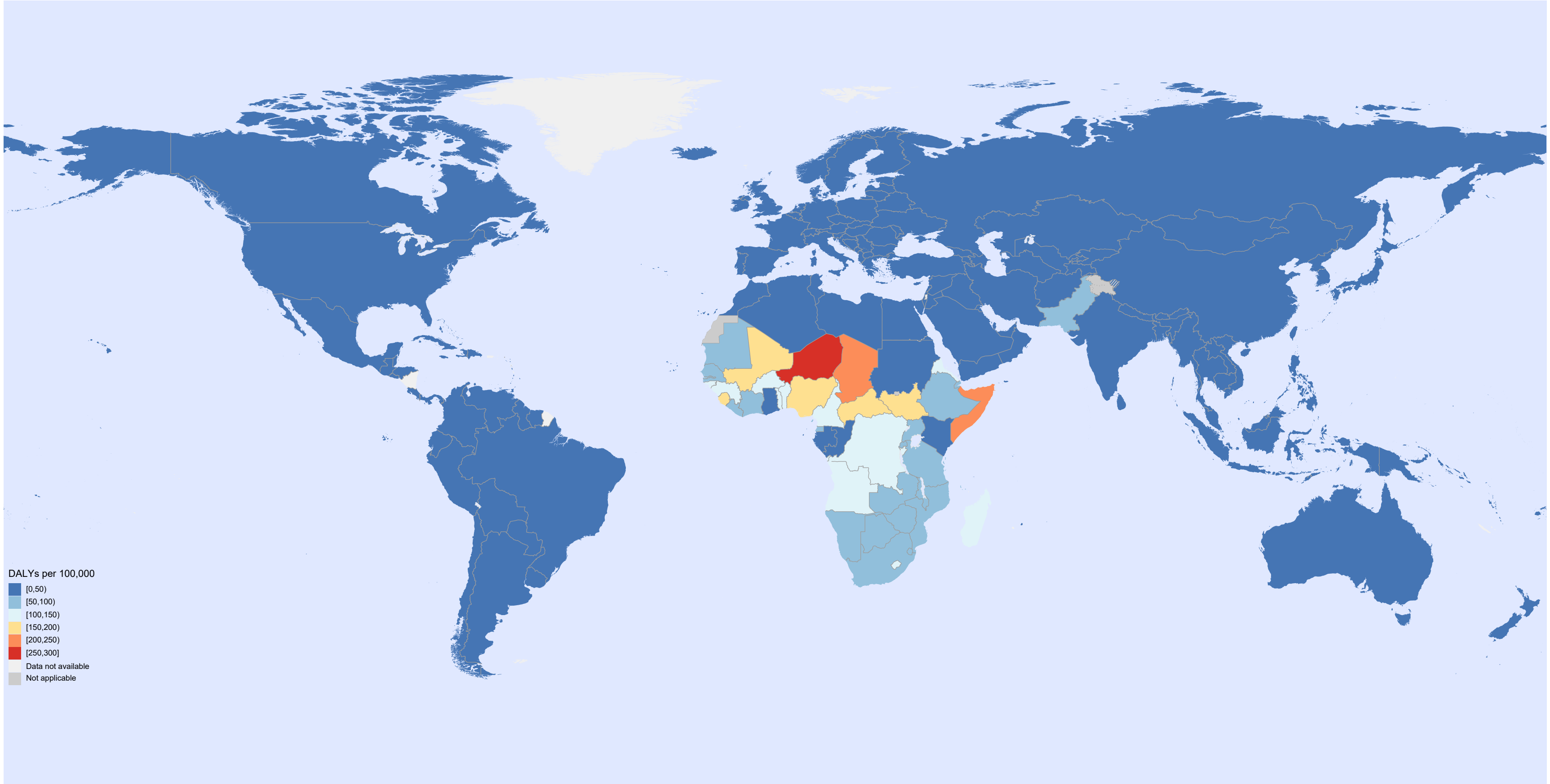

Figure S18. Mean national rates of foodborne Disability-Adjusted Life Years (DALYs) per 100,000 persons due to *Vibrio cholerae*, 2021 (data in appendix 2)

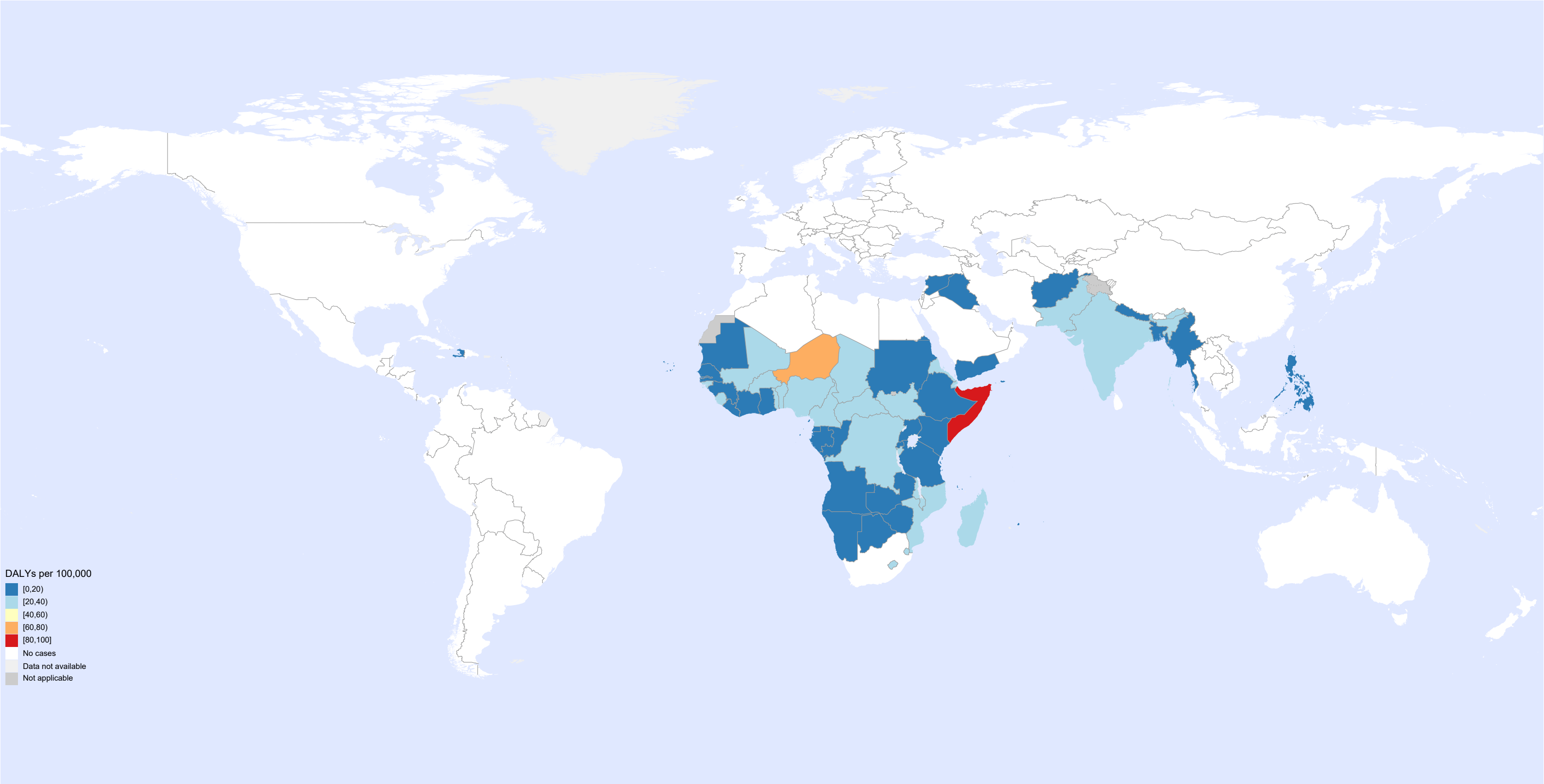

GATHER AND STROBOD CHECKLISTS

Checklist of information that should be included in new reports of global health estimates

Item #	Checklist item	Reported on page #
**Objectives and funding**		
**1**	Define the indicator(s), populations (including age, sex, and geographic entities), and time period(s) for which estimates were made.	Introduction paragraph 2<br>Methods paragraphs 3 and 5
**2**	List the funding sources for the work.	Methods, role of the funding source paragraph<br>Back matter, funding statement
**Data Inputs**		
For all data inputs from multiple sources that are synthesized as part of the study:		
**3**	Describe how the data were identified and how the data were accessed.	Methods paragraphs<br>Supplementary Methods Appendix<br>References 2 and 9
**4**	Specify the inclusion and exclusion criteria. Identify all ad-hoc exclusions.	Supplementary Methods Appendix<br>References 2 and 9
**5**	Provide information on all included data sources and their main characteristics. For each data source used, report reference information or contact name/institution, population represented, data collection method, year(s) of data collection, sex and age range, diagnostic criteria or measurement method, and sample size, as relevant.	Supplementary Methods Appendix<br>Reference 2 and 9<br>Acknowledgements
**6**	Identify and describe any categories of input data that have potentially important biases (e.g., based on characteristics listed in item 5).	n/a
For data inputs that contribute to the analysis but were not synthesized as part of the study:		
**7**	Describe and give sources for any other data inputs.	Supplementary Methods Appendix<br>References 2, 9, and 15
For all data inputs:		
**8**	Provide all data inputs in a file format from which data can be efficiently extracted (e.g., a spreadsheet rather than a PDF), including all relevant meta-data listed in item 5. For any data inputs that cannot be shared because of ethical or legal reasons, such as third-party ownership, provide a contact name or the name of the institution that retains the right to the data.	Back matter, data sharing statement<br>Acknowledgements
**Data analysis**		
**9**	Provide a conceptual overview of the data analysis method. A diagram may be helpful.	Methods paragraphs<br>Reference 9
**10**	Provide a detailed description of all steps of the analysis, including mathematical formulae. This description should cover, as relevant, data cleaning, data pre-processing, data adjustments and weighting of data sources, and mathematical or statistical model(s).	Supplementary Methods Appendix<br>Reference 9
**11**	Describe how candidate models were evaluated and how the final model(s) were selected.	Reference 9
**12**	Provide the results of an evaluation of model performance, if done, as well as the results of any relevant sensitivity analysis.	Reference 9
**13**	Describe methods for calculating uncertainty of the estimates. State which sources of uncertainty were, and were not, accounted for in the uncertainty	Reference 9

**14**	State how analytic or statistical source code used to generate estimates can be accessed.	Back matter, data sharing statement
**Results and Discussion**		
**15**	Provide published estimates in a file format from which data can be efficiently extracted.	Supplementary Results Files
**16**	Report a quantitative measure of the uncertainty of the estimates (e.g. uncertainty intervals).	Throughout Results, Tables, and Figures (and their data tables)
**17**	Interpret results in light of existing evidence. If updating a previous set of estimates, describe the reasons for changes in estimates.	Discussion
**18**	Discuss limitations of the estimates. Include a discussion of any modelling assumptions or data limitations that affect interpretation of the estimates.	Discussion Reference 9

This checklist should be used in conjunction with the GATHER statement and Explanation and Elaboration document, found on gather-statement.org

Table 1 STROBOD checklist of items that should be included in reports of Disability-adjusted life year calculations

From: Standardised reporting of burden of disease studies: the STROBOD statement

Item number	Domains and description of the recommended items	Reported on page number
Title		
1	Identify the study as a burden of disease assessment by including keywords (e.g., Years of Life Lost, Years Lost due to Disability, Disability-Adjusted Life Years, burden of disease etc.), and describe the study setting	Title
Abstract		
2	Provide a summary of objectives, study setting, methods (including data sources and key methodological design choices used), results (including point estimates and, if applicable, uncertainty intervals), and conclusions	Abstract
Introduction		
3	Present background information to the study, its study aim(s), and its relevance for health policy or practice	Introduction
Methods		

Item number	Domains and description of the recommended items	Reported on page number
Study setting		
4	Report for which cause(s) the burden was calculated. Provide a case definition, e.g., in terms of an internationally recognized classification system such as the International Classification of Diseases and Related Health Problems 10th Revision	Introduction paragraph 2
5	Report the reference population and any stratification of the reference population for the burden of disease assessment, i.e., the population for which the burden was calculated. This may include the geographical location (e.g., country or province/state), and whether the general population or a specific subset of the population (e.g., females, adolescents aged 10–19 years, etc.) was considered	Methods paragraphs 3 and 5<br>Suppl. Methods Appendix
6	Report the reference time period (e.g., year(s), month(s)) of the study. This refers to the time period to which the burden of disease estimates refer	Introduction paragraph 2
Epidemiological and demographic input data		
7	Report the sources, values, ranges, and, if used, probability distributions for all epidemiological input parameters. Report reasons or sources for distributions used to represent uncertainty where appropriate. Providing a (supplementary) table to show all epidemiological input parameters and respective sources and assumptions is strongly recommended	Suppl. Methods Appendix<br>References 2 and 9

Item number	Domains and description of the recommended items	Reported on page number
8	Describe all possible data manipulations, such as bias corrections, data integration steps, or methods to ensure internal consistency of the data inputs	References 2 and 9
9	Report the sources and values of any population data used. If applicable, report the standard population used to calculate age-standardized rates	Reference 2 and 9
Daly methods		
10	Report the age-conditional life expectancy used for calculating Years of Life Lost (i.e., national, regional, or aspirational life tables) or other methods (e.g., potential years of life lost, proportion of premature deaths under a selected age threshold etc.)	Reference 9
11	Report the perspective taken for calculating Years Lost due to Disability, i.e., incidence or prevalence perspective	Reference 9
Disease model		
12	Describe the disease model. Present and justify the included health outcomes and health states. Providing a (supplementary) figure visualizing the disease model is strongly recommended	Suppl. Methods Appendix<br><br>Reference 9

Item number	Domains and description of the recommended items	Reported on page number
13	Report the source(s) and values of the used disability weights. Providing a (supplementary) table depicting the health states, brief lay descriptions, and the numerical values followed by its uncertainty intervals is strongly recommended	Suppl. Methods Appendix
14	If new disability weights were elicited, provide information on how the health states were described and the elicitation procedures. As a minimum to the latter, describe which valuation technique was used and which reference group and size of the group (also known as panel of judges) evaluated the health states. Providing a supplementary table with a description of the valuation technique and brief lay descriptions used is strongly recommended	n/a
15	Report the source(s) and values of the used durations (if applicable). Providing a (supplementary) table depicting the health states and the numerical values followed by its uncertainty intervals is strongly recommended	Suppl. Methods Appendix
16	Report the source(s) and values of the used conditional probabilities, severity distribution, and/or transition rates. Providing a (supplementary) table depicting the parent/child health outcomes and health states and the numerical values followed by its uncertainty intervals is strongly recommended	Suppl. Methods Appendix<br>Reference 9
Multimorbidity adjustments		

Item number	Domains and description of the recommended items	Reported on page number
17	Report whether or not multimorbidity adjustments were applied to any of the input variables in the estimation of Years Lost due to Disability. If applied, describe which multimorbidity adjustment method was used	Reference 9
Social weighting factors		
18	Report whether or not age weighting was applied. If applied, describe which parameters were used	Reference 9
19	Report whether or not time discounting was applied. If applied, describe which discount rate was used	Reference 9
Uncertainty and scenario analysis		
20	Describe any methods used to perform uncertainty and variable importance (sensitivity) analyses. If, for example, Monte Carlo simulations were used, report the number of iterations	Reference 9
21	Describe any scenario analyses that were performed. Present the rationale and the alternative data inputs defining the alternative scenarios	Reference 9
Results		

Item number	Domains and description of the recommended items	Reported on page number
22	Report the point estimates and, if applicable, the uncertainty interval of the burden of disease estimates. Provide both absolute values, crude rates (optional), and age-standardized rates per 100,000 in a table or figure	Results and Tables
23	If applicable, report the results of the scenario analyses. Tables and/or figures illustrating findings on the scenario analyses are strongly recommended	n/a
Discussion		
24	Summarise the key study findings and describe how they support the conclusions reached	Discussion
25	Discuss how the findings fit within current knowledge. Discuss potential implications for public health practice. Compare the results with those of other studies, and discuss methodological design differences, if relevant	Discussion
26	Discuss strengths and limitations, and the generalisability of the study findings. If applicable, discuss the results of the uncertainty and scenario analyses	Discussion
Open science		
27	Make the source code or computational model(s) available as supporting information or via a dedicated open access repository (e.g., GitHub)	Back matter, data sharing statement

Item number	Domains and description of the recommended items	Reported on page number
28	Describe how the study was funded and the role of the funder in the identification, design, conduct, and reporting of the analysis. Describe other non-monetary sources of support or any potential conflict(s) of interest of the study contributor(s) in accordance with the journal policy	Methods, role of the funding source paragraph<br>Back matter, funding statement
